# Spatiotemporal Analysis Reveals Overlap of Key Proepicardial Markers in the Developing Murine Heart

**DOI:** 10.1016/j.stemcr.2020.04.002

**Published:** 2020-04-30

**Authors:** Irina-Elena Lupu, Andia N. Redpath, Nicola Smart

**Affiliations:** 1 Department of Physiology, Anatomy & Genetics, University of Oxford, Oxford OX1 3PT, UK

**Keywords:** epicardium, EPDCs, sub-compartment, heterogeneity, proepicardium, Wt1, Tcf21, Tbx18, Sema3d, Scx

## Abstract

The embryonic epicardium, originating from the proepicardial organ (PEO), provides a source of multipotent progenitors for cardiac lineages, including pericytes, fibroblasts, and vascular smooth muscle cells. Maximizing the regenerative capacity of the adult epicardium depends on recapitulating embryonic cell fates. The potential of the epicardium to contribute coronary endothelium is unclear, due to conflicting Cre-based lineage trace data. Controversy also surrounds when epicardial cell fate becomes restricted. Here, we systematically investigate expression of five widely used epicardial markers, *Wt1*, *Tcf21*, *Tbx18*, *Sema3d*, and *Scx*, over the course of development. We show overlap of markers in all PEO and epicardial cells until E13.5, and find no evidence for discrete proepicardial sub-compartments that might contribute coronary endothelium via the epicardial layer. Our findings clarify a number of prevailing discrepancies and support the notion that epicardium-derived cell fate, to form fibroblasts or mural cells, is specified after epithelial-mesenchymal transition, not pre-determined within the PEO.

## Highlights

• PEO and epicardial cells co-express *Wt1*, *Sema3d*, *Tbx18*, *Scx*, and *Tcf21*
• Markers are not confined to (pro)epicardium and cannot individually define the lineage
• Distinctive marker changes accompany epithelial-mesenchymal transition
• *Tcf21* is upregulated in epicardium-derived progenitors, other markers are downregulated

## Graphical Abstract

**Figure:**
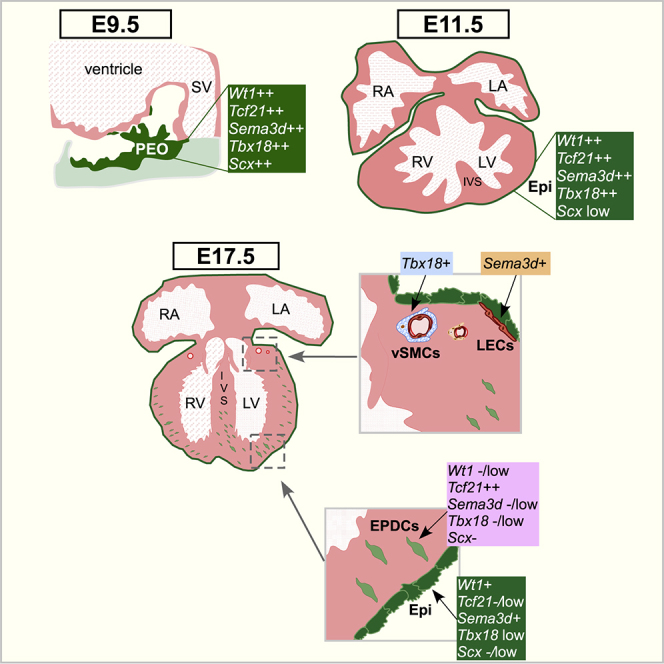

## Teaser

Lupu, Redpath, and Smart systematically profile expression of canonical markers *Wt1*, *Sema3d*, *Tbx18*, *Scx*, and *Tcf21* throughout murine cardiac development. They demonstrate overlapping expression in all proepicardial organ (PEO) and epicardial cells and describe how this marker signature changes upon epithelial-mesenchymal transition. Their findings refute the existence of discrete PEO sub-compartments with pre-determined fates.

## Introduction

Recent research has led to a detailed understanding of how the coronary vasculature forms during development, with Cre-*loxP* genetic lineage tracing identifying the sinus venosus and ventricular endocardium as the main contributors of coronary endothelial cells (CECs) (Chen et al., 2014, Red-Horse et al., 2010, Tian et al., 2013, Tian et al., 2014, Zhang et al., 2016). However, a distinct sub-compartment of the proepicardium (PEO) expressing *Sema3d* and *Scx*, largely non-overlapping with the previously described PEO markers *Wt1* and *Tbx18*, was also proposed to give rise to CECs (Katz et al., 2012), raising questions about the vasculogenic potential of epicardial cells. The PEO is a transient embryonic structure that contains epicardial progenitor cells and, in mammals, arises near the septum transversum (STM) from posterior second heart field progenitors (Kruithof et al., 2006, Lie-Venema et al., 2007). PEO cells migrate onto the murine heart surface from embryonic day 9.5 (E9.5) to form the epicardium. Intriguingly, several studies reported ubiquitous expression of *Wt1*, *Tcf21*, and *Tbx18* in the early epicardium (Acharya et al., 2012, Wei et al., 2015), suggesting that these non-overlapping PEO sub-populations may not translate to the epicardium proper. Cre-based lineage tracing, driven by promoters of epicardial genes *Wt1*, *Tbx18*, *Tcf21*, or an enhancer of *Gata5*, reported minimal CEC contribution (Acharya et al., 2012, Cai et al., 2008, Merki et al., 2005, Zhou and Pu, 2012), whereas the discrete PEO sub-compartments, expressing *Sema3d* and/or *Scx*, were reported to contribute: 7% of CECs at E16.5 from the *Sema3d* lineage and 25% of CECs postnatally from the *Scx* lineage (Katz et al., 2012).

Another matter under scrutiny is whether epicardial fate is pre-specified within the PEO or if these cells are multipotent. Epicardial cells undergo epithelial-mesenchymal transition (EMT) from E12.5, giving rise to epicardium-derived cells (EPDCs). Although EPDCs are accepted to differentiate into pericytes, progenitors for coronary vascular smooth muscle cells (vSMCs) (Volz et al., 2015), and cardiac fibroblasts (CFs), it remains unclear what guides their cell fate choice, but *Tcf21* is thought to be a pre-determinant of CF fate (Acharya et al., 2012, Braitsch et al., 2012).

Here, we reveal co-expression of all previously reported markers in the PEO and the entire epicardial layer early in development, finding no support for the putative sub-compartments that might contribute coronary endothelium via the epicardial layer. We also provide evidence to suggest that epicardium-derived cell fate is specified only after EMT, seemingly in response to environmental cues, and importantly marker expression profile—in the PEO or epicardium—does not restrict cell fate choice. Thus, our findings challenge previous concepts around the existence of discrete epicardial sub-populations with pre-determined cell fates.

## Results

### Wt1, Sema3d, Tbx18, Scx, and Tcf21 Overlap in the PEO, but Their Expression Domains Are Not Confined to This Tissue

**Figure 1:**
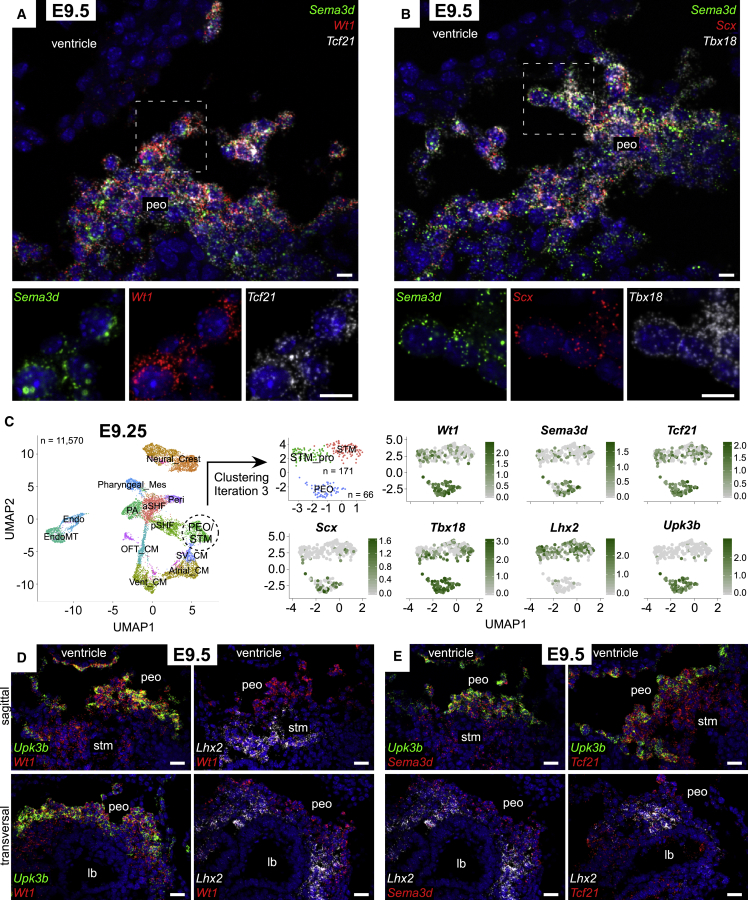
*Sema3d*, *Wt1*, *Tcf21*, *Tbx18*, and *Scx* Are Co-expressed in Proepicardial Cells (A and B) *In situ* hybridization (ISH) of E9.5 embryos for *Sema3d*, *Wt1*, *Tcf21* (A) and *Sema3d*, *Scx*, *Tbx1*8 (B) mRNA (n = 5). (C) UMAP plot showing the major clusters in E9.25 scRNA-seq data (total 11,570 cells, n = 2 embryos). UMAP plot showing the PEO and STM cells after three clustering iterations of the PEO/STM cluster. Feature plots representing range of expression of *Wt1*, *Sema3d*, *Tcf21*, *Scx*, *Tbx18*, *Lhx2*, and *Upk3b* in individual cells of the PEO and STM subclusters. (D) ISH of E9.5 embryos, sagittal and transversal sections, for *Upk3b*, *Wt1*, and *Lhx2*, showing expression of *Wt1* in the PEO and STM (n = 3). (E) ISH of E9.5 sagittal sections for *Upk3b*, *Sema3d*, and *Tcf21*, and transversal sections for *Lhx2*, *Sema3d*, and *Tcf21*, demonstrating expression of *Sema3d* and *Tcf21* in the PEO and STM, respectively (n = 3). EndoMT, endocardial-to-mesenchymal transition; Endo, endocardial cells; Mes, mesenchyme; PA, pharyngeal arch; aSHF, anterior second heart field; Peri, pericardium; pSHF, posterior second heart field; OFT_CM, outflow tract cardiomyocytes; Vent_CM, ventricular cardiomyocytes; SV_CM, sinus venosus cardiomyocytes; PEO/STM, proepicardium/septum transversum; lb, liver bud. Scale bars, 10 μm (A and B) and 20 μm (D and E). See also Figure S1.

First, we used multiplexed single-molecule RNA *in situ* hybridization (RNAscope) on E9.5 sagittal mouse sections to simultaneously detect expression of the PEO markers: *Wt1*, *Sema3d*, *Tcf21*, *Scx*, and *Tbx18*, having established that signals were highly reproducible, regardless of whether probes were used individually or in combination. We detected complete overlap of marker expression in both the PEO and in cells actively migrating out and onto the heart (Figures 1A and 1B). Morphologically, proepicardial cells are defined as protrusions/villi that extend from the STM region (Maya-Ramos et al., 2013). Our data show that the bona fide PEO ubiquitously co-expresses all tested markers. In contrast, partial overlap was clearly evident below the PEO, in the STM, where distinct expression domains were present (Figure S1A).

To further investigate marker expression in PEO cells, we analyzed published single-cell RNA sequencing (scRNA-seq) data from whole heart and surrounding tissue at E9.25 (de Soysa et al., 2019). Principal component analysis revealed 14 clusters, largely corresponding to neural crest cells, endothelial cells, cardiac progenitor cells, and cardiomyocyte (CM) subsets (Figures 1C and S1B). Initially, PEO and STM cells clustered together due to their similar transcriptomic profiles. Three subsequent clustering iterations separated PEO cells from STM cells, which expressed markers, such as *Lhx2* and *Foxf1* (Kalinichenko et al., 2002, Kolterud et al., 2004, Ren et al., 2014), and included cardiac progenitors positive for *Hand1* (Barnes et al., 2011). The PEO cluster identity was confirmed based on known markers, such as *Lhx9* (Tandon et al., 2016) and mesothelial gene *Upk3b* (Rudat et al., 2014) (Figures 1C and S1C). *Wt1* and *Tbx18* expression was found in 100% of PEO cells, and *Tcf21* was present in 97%. Detection of *Sema3d* and *Scx* was lower, at 70% and 55%, respectively, likely due to limited sensitivity of 10× Chromium technology which only detects highly expressed genes (Baran-Gale et al., 2017). RNAscope, which offers the sensitivity to detect single-molecule RNA, demonstrated expression of all markers throughout the PEO, as shown in our data. The canonical proepicardial genes *Wt1*, *Tcf21*, *Tbx18*, *Sema3d*, and *Scx* were also detected in some cells of the STM cluster, indicating that these genes are not restricted to the PEO (Figure 1C); however, all were enriched in PEO relative to STM. To validate the scRNA-seq data, RNAscope probes against *Upk3b* (PEO marker) and *Lhx2* (STM marker) were used to refine the expression domains of *Wt1*, *Sema3d*, *Tcf21* (Figures 1D, 1E, S1D, and S1E), *Tbx18*, and *Scx* (Figures S1D and S1E) in both sagittal and transversal sections throughout the PEO. The markers completely overlapped with one another in *Upk3b*-expressing cells, demarcating the PEO, but their expression was heterogeneous within the underlying STM, delineated by *Lhx2* expression, consistent with known expression of *Wt1* and *Tcf21* in the hepatic primordium (Lu et al., 2000, Perez-Pomares et al., 2004) and *Tbx18* in CM precursors, located in the STM region (Christoffels et al., 2009).

### Epicardial Founder Cells Co-express *Wt1*, *Sema3d*, *Tbx18*, *Scx*, and *Tcf21*

**Figure 2:**
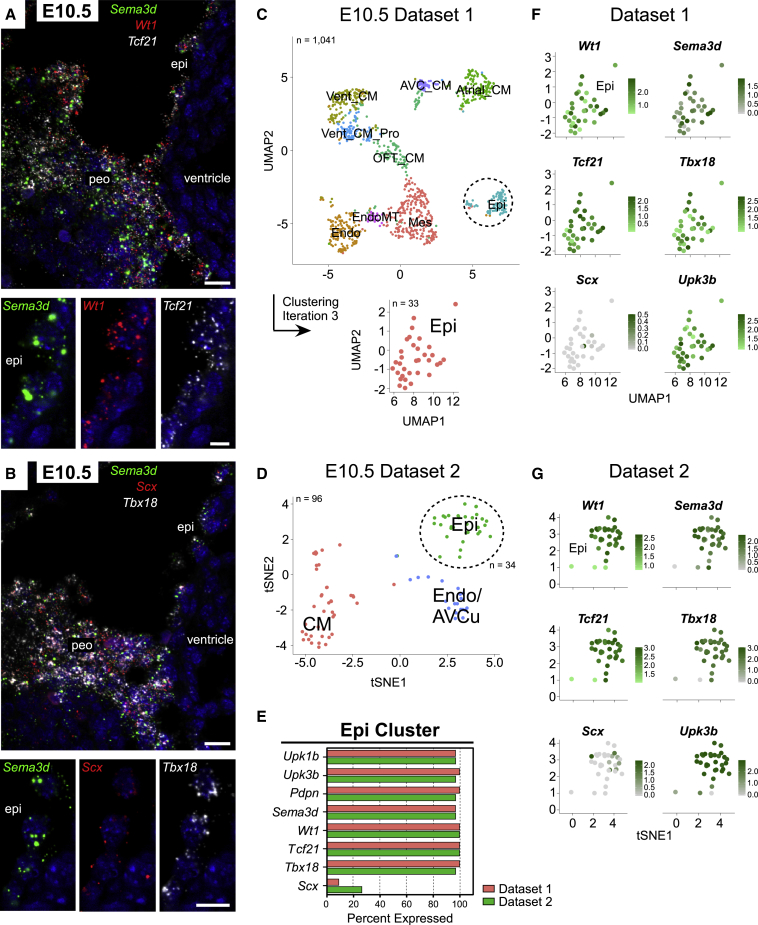
Complete Marker Overlap in Founder Epicardial Cells (A and B) ISH of E10.5 embryos for *Sema3d*, *Wt1*, *Tcf21* (A) and *Sema3d*, *Scx*, *Tbx1*8 (B) mRNA (n = 2 embryos). (C) UMAP plot demonstrating the different major clusters in E10.5 heart scRNA-seq dataset 1 (total 1,041 cells, n = 2 batches). UMAP plot showing the epicardial (Epi) cluster after three clustering iterations to remove doublets. (D) tSNE plot clustering of cardiomyocytes (CM), endocardium/atrioventricular cushion (Endo/AVCu), and epicardial cells (Epi) in E10.5 heart scRNA-seq dataset 2 (total 96 cells; n = 2 hearts). (E) Percentage of epicardial cluster expressing selected gene. (F) Feature plots representing range of expression of *Wt1*, *Sema3d*, *Tcf21*, *Tbx18*, *Scx*, and *Upk3b* in individual cells of dataset 1 epicardial cluster. For genes expressed in 100% of the cluster, expression ranges from light green to dark green, for genes expressed in less than 100%, expression ranges from light gray to dark green. (G) Feature plots representing range of expression of *Wt1*, *Sema3d*, *Tcf21*, *Tbx18*, *Scx*, and *Upk3b* in individual cells of dataset 2 epicardial cluster. peo, proepicardial organ; Vent_CM, ventricular cardiomyocytes, Pro, proliferating; OFT_CM, outflow tract cardiomyocytes; Mes, mesenchyme; EndoMT, endocardial-to-mesenchymal transition; Endo, endocardium; AVC_CM, atrioventricular canal cardiomyocytes; Epi, epicardium. Scale bars, 20 μm (A and B low power) and 10 μm (A and B high power). See also Figure S2.

To profile PEO cells that reach the heart and form the definitive epicardium, we multiplexed RNAscope probes on E10.5 sagittal sections. Cells in contact with the heart surface co-expressed all markers, albeit *Scx* was decreased in all cells compared with E9.5 (Figures 2A, 2B, and S2A), as reported previously (Katz et al., 2012). No cells with partially overlapping expression, such as from the underlying STM, were visualized to transition onto the heart. To independently confirm RNAscope findings, we analyzed two published E10.5 mouse heart scRNA-seq datasets (Dong et al., 2018, Li et al., 2016). E10.5 dataset 1 (Li et al., 2016) comprised ten populations, largely corresponding to discrete CM types, endocardial, mesenchymal, and epicardial cells (Figures 2C and S2B). Doublet abundance was evident during our analysis of this dataset, and reported in the original study (Li et al., 2016). Two-thirds of the Epi cluster constituted doublets that were successfully removed after three subsequent clustering iterations (Figures S2C and S2D). E10.5 dataset 2 (Dong et al., 2018) consisted of three populations: epicardium, endocardium/atrioventricular cushion (AVCu), and CMs (Figures 2D and S2E). Across both independent datasets, *Wt1*, *Sema3d*, *Tcf21*, and *Tbx18* were detected in 97%–100% of the epicardial cluster, alongside mesothelial genes, such as *Upk3b* and *Upk1b* (Figure 2E). *Scx* was only detected in a small percentage of cells (Figure 2E), but this likely reflects the limited capture rate of scRNA-seq technology, since *Scx* is expressed at very low levels, with few transcripts per cell evident by RNAscope (Figure 2B). The level of gene expression within individual cells of the epicardial clusters is shown as feature plots (Figures 2F and 2G).

### Canonical Epicardial Markers Remain Co-expressed in the E11.5 Epicardium

**Figure 3:**
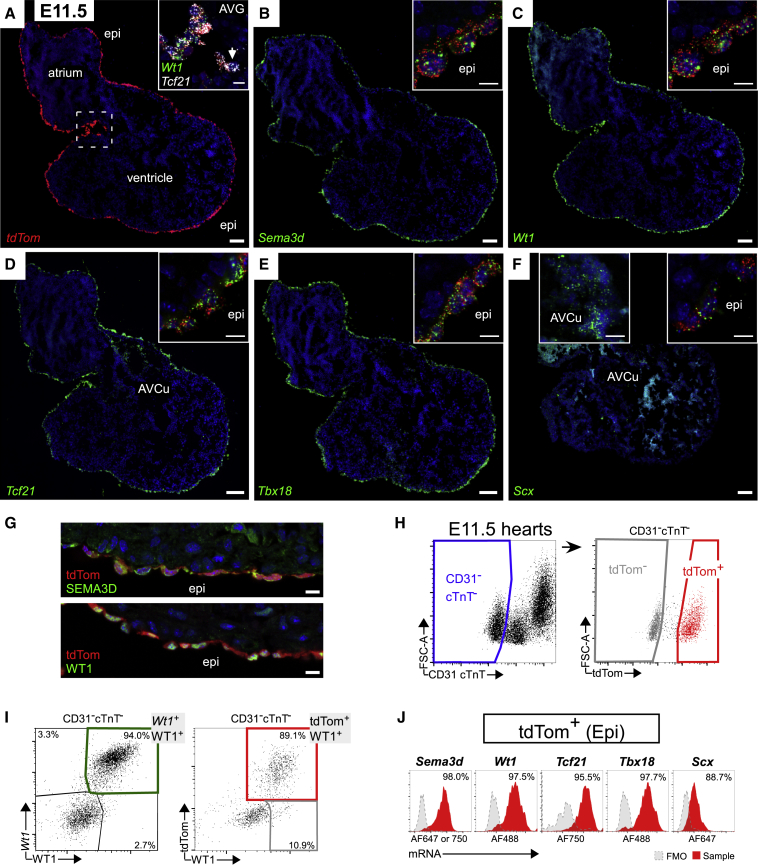
*Sema3d*, *Wt1*, *Tcf21*, *Tbx18*, and *Scx* Are Co-expressed Throughout the Epicardial Layer (A) ISH of E11.5 hearts shows tdTom labeling of the epicardium (epi), induced at E9.5 in Wt1*CreERT2* embryos (n = 4 hearts). Inset shows cells in atrioventricular groove (AVG) co-expressing *Tcf21* and *tdTom*, and downregulated *Wt1* (arrow). (B–F) ISH of E11.5 hearts for (B) *Sema3d*, (C) *Wt1*, (D) *Tcf21*, (E) *Tbx18*, and (F) *Scx* with *tdTom*-labeled epicardium (insets) (n = 3 hearts). (G) Immunostaining of E11.5 heart cryosections for SEMA3D and WT1 reveals expression of SEMA3D and WT1 throughout the epicardial layer, marked by tdTom (n = 3). (H) Strategy used to gate tdTom+ cells in flow cytometric analysis of E11.5 hearts. Cardiomyocytes and endothelial cells were excluded based on expression of cardiac troponin T (cTnT) and CD31, respectively. (I) Correlation between *Wt1* transcript and WT1 protein levels (94% overlap) (n = 21 hearts; 3 independent experiments). Correlation between WT1 expression and tdTomato labeling efficiency (89%) (n = 8 hearts; 2 independent experiments). (J) Flow cytometric analysis of E11.5 hearts. Epicardial cells, selected by gating CD31-cTnT-tdTom+, show co-expression of *Sema3d*, *Wt1*, *Tcf21*, *Tbx18*, and *Scx* (n = 31 hearts; 3 independent experiments). Fluorescence minus one (FMO) control and percentage positive shown. AVG, atrioventricular groove; AVCu, atrioventricular cushion. Scale bars, 100 μm (A–F); 10 μm (inset A); and 10 μm (inset B–F and G). See also Figure S3.

A key question is how epicardial marker expression changes throughout development, both within the epicardial layer and in the derivative EPDCs as they differentiate. We used the inducible Wt1CreERT2, crossed with R26R-tdTomato (tdTom) reporter, to enable tracing of the epicardial lineage even after epicardial genes are downregulated. As maximal Cre recombination occurs 24–48 h after tamoxifen delivery (Hayashi and McMahon, 2002, Zhou and Pu, 2012), we administered tamoxifen at E9.5 to label the epicardium at E10.5–E11.5 before EMT is initiated (Hayashi and McMahon, 2002, Zhou and Pu, 2012). RNAscope was performed at E11.5, when epicardial formation is complete, multiplexing probes against epicardial markers and *tdTom.* TdTom efficiently labeled the epicardial layer, and inspection of high-power images indicated overlap with all tested markers throughout the epicardial layer (high/low power, Figures 3A–3F; medium power, Figure S3A). As the epicardial layer is non-contiguous in places, even at E11.5, *TdTom* probe was additionally multiplexed with *Upk3b*, an accepted ubiquitous epicardial marker (Rudat et al., 2014, Xiao et al., 2018), to demonstrate efficient labeling of cells in the epicardial layer (Figure S3B). The only domain that did not present overlapping expression of *Upk3b* and *tdTom* was a distinct population of *tdTom*+ cells in the atrioventricular groove (AVG), which were highly positive for *Tcf21* (Figures S3B inset and 3A inset). These cells derived from the *Wt1* lineage, as indicated by *tdTom* expression, but downregulated *Wt1*, *Tbx18*, *Sema3d*, *Scx*, and *Upk3b*, and upregulated *Postn*, indicating mesenchymal state (Figure S3B inset), consistent with the earliest transition to EPDCs in this region (Krainock et al., 2016). Some markers were detected in non-epicardial domains, *Tbx18* in CMs (Figure 3E), *Scx* and *Tcf21* in AVCu (Figures 3D–3F), confirming previous reports (Acharya et al., 2011, Barnette et al., 2014, Christoffels et al., 2009).

A caveat of our study, and others, is the over-reliance on mRNA and reporter readouts due to the inadequacies of available antibodies. To confirm our findings at the protein level, antibodies against WT1 and SEMA3D were used (Figure 3G), while all TBX18, TCF21, and SCX antibodies tested produced non-specific or no staining (not shown). WT1 and SEMA3D proteins were detected uniformly throughout the epicardial layer (Figures 3G and S3C). To confirm correlation between *Wt1* mRNA and WT1 protein levels, and to quantify overlap at the single (whole)-cell level, a flow cytometry-based RNA *in situ* hybridization method was utilized (PrimeFlow RNA Assay) on enzymatically dissociated E11.5 hearts (Figure 3H). Of all cells labeled by *Wt1* probe and/or WT1 antibody, 94% were strongly positive for both (Figure 3I). To assess tdTom labeling efficiency and to exclude the possibility that slight variations in *Wt1* expression might influence recombination, we analyzed overlap of both *Wt1* and WT1 expression with tdTom, which revealed 88.5% of *Wt1*+ cells (Figure S3D) and 89.1% of WT1+ cells to be tdTom labeled (Figure 3I); considered highly efficient for an inducible Cre line (Hayashi and McMahon, 2002). It should be noted that even the non-labeled cells expressed WT1 and other epicardial markers at similar levels to the tdTom-labeled cells, indicating that recombination is stochastic and not correlated with expression level of the endogenous driver (e.g., 97% and 92% of TdTom- *Wt1*+ cells expressed *Sema3d* and *Tcf21*, respectively; Figure S3D). Co-expression of *Wt1*, *Tcf21*, *Sema3d*, and *Tbx18* was detected in >95% of tdTom+ cells (Figure 3J), confirming the RNAscope results. Scx expression was very low and detected only in 88.7% (Figure 3J).

### Markers Are Sequentially Downregulated in the Epicardium as Development Progresses

**Figure 4:**
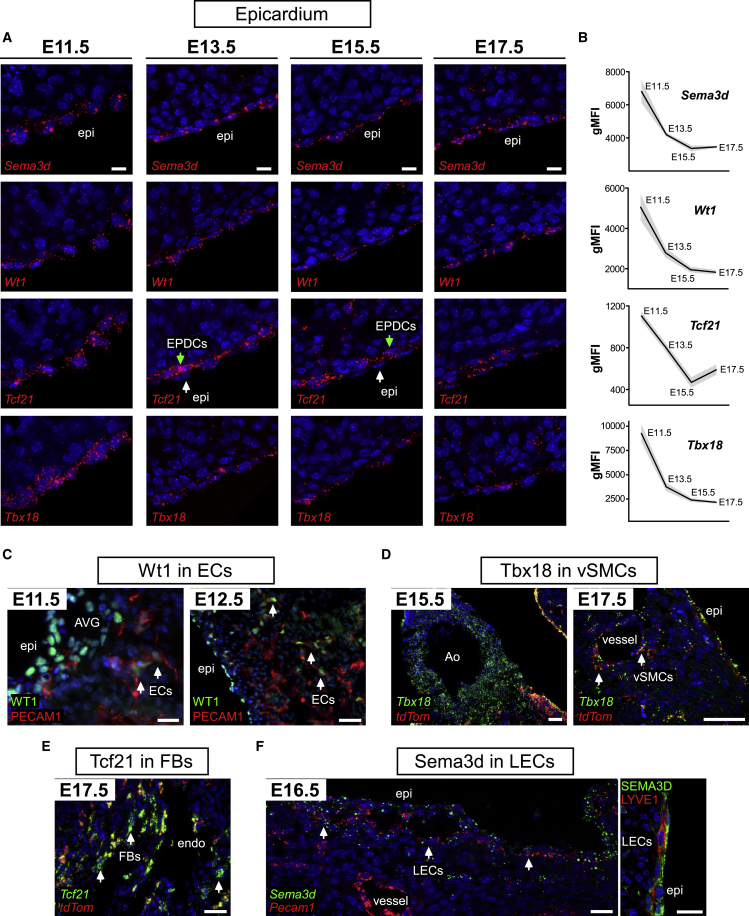
Epicardial Genes Are Downregulated in the Epicardium as Development Progresses and Become Expressed in Non-epicardial Domains (A) ISH of E11.5, E13.5, E15.5, and E17.5 hearts show expression of *Sema3d*, *Wt1*, *Tcf21*, *Tbx18*, and *Scx* in the epicardium (n = 3). (B) Flow cytometric analysis of *Sema3d*, *Wt1*, *Tcf21*, *Tbx18*, and *Scx* in the epicardial population (mean gMFI ± SEM; four independent experiments with exception of Tbx18, n = 2). gMFI, geometric mean fluorescence intensity. (C) Immunostaining for WT1 and PECAM1 on E11.5 and E12.5 heart sagittal sections reveals expression of WT1 in endothelial cells (ECs). (D) ISH for *Tbx18* and *tdTom* on E15.5 and E17.5 heart sections reveals expression of *Tbx18* in vSMCs; both in non-epicardium-derived cells in the aorta (Ao) and in epicardium-derived cells in coronary vessels. (E) ISH for *Tcf21* and *tdTom*o on E17.5 heart sections reveals high *Tcf21* expression in fibroblasts of non-epicardial origin, near the endocardial surface. (F) ISH for *Sema3d* and *Pecam1* and immunostaining for SEMA3D and LYVE1 reveals SEMA3D expression in cardiac lymphatic endothelial cells (LECs) in E16.5 heart sections. Scale bars, 10 μm (A and C) E11.5; 50 μm (C) E12.5 and (D); and 20 μm (E and F). See also Figure S4.

RNAscope and PrimeFlow were used to assess expression of selected markers in the epicardial layer throughout development (Figures 4A and S4A–S4G). In addition to E11.5, three other key developmental stages were chosen for analysis; E13.5, when most EPDCs emerge; E15.5, when EMT is complete; and E17.5, when EPDCs have differentiated. Within the epicardial layer, expression of *Sema3d*, *Wt1*, *Tcf21*, and *Tbx18* peaked at E11.5 (Figures 4A and 4B). *Tcf21* expression was dramatically reduced beyond E13.5, followed by a reduction in *Tbx18* expression, to coincide with completion of epicardial EMT by E15.5 and onset of epicardial quiescence (Liu et al., 2016). *Wt1* and *Sema3d* continued to be co-expressed in the entire epicardium throughout development, albeit their levels decreased (Figures 4A, 4B, S4A, and S4B). Although markers were downregulated, their expression remained discernibly homogeneous between individual epicardial cells (Figure 4A) and quantitatively shown to be within a limited dynamic range (Figure 4B).

The specificity of selected markers to the tdTom-labeled epicardial lineage was assessed across the developmental time course (Figures 4C–4F and S4A–S4E). Marker expression increasingly extended to non-epicardial domains by later stages. Surprisingly, WT1 expression was detected in the coronary endothelium as early as E11.5, as revealed by co-staining of WT1 and PECAM1 on sagittal sections (Figure 4C). This was further accentuated by E12.5 (Figure 4C), as previously reported (Duim et al., 2015). *Tbx18* was expressed in vSMCs, both of non-epicardial (in aorta) and epicardial origin (in coronary vessels) (Figure 4D), validating published reports of *de novo Tbx18* expression in most mural cells in the mouse (Guimaraes-Camboa et al., 2017). We also confirmed expression of *Tbx18* in CMs of the left ventricle and IVS (Figure S4E) (Christoffels et al., 2009). *Tcf21* was found in AVG (Acharya et al., 2011, Zhou and Pu, 2012) and in a substantial number of non-epicardium-derived fibroblasts near the endocardial surface (Figures 4E and S4C). Intriguingly, from E16.5, *Sema3d* was expressed in a subset of endothelial cells within the subepicardial space (Figure 4F). Co-staining with LYVE1 showed that SEMA3D is expressed in cardiac lymphatics (not epicardial derived), but not in coronary blood vessel endothelium (Figure 4F). *Sema3d* and *Scx* were also expressed in the AVCu (Figures S4A and S4D), as reported (Katz et al., 2012).

### Epicardial Cells Lose Their Marker Signature upon EMT

**Figure 5:**
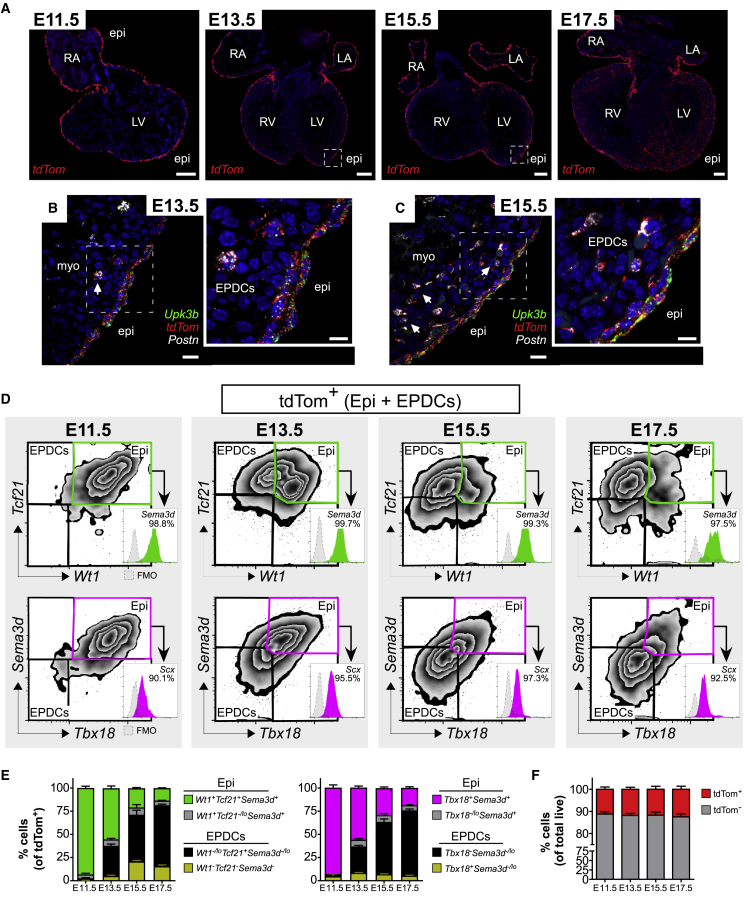
Epicardium-Derived Progenitors Lose Epicardial Signature (A) ISH of E11.5, E13.5, E15.5, and E17.5 hearts shows tdTom labeling of the epicardium and lineage-traced EPDCs (n = 3 hearts/stage). Boxed regions indicate magnified regions in (B and C). (B) ISH of E13.5 heart sections for *Upk3b*, *tdTom*, and *Postn* reveal the appearance of *Upk3b*-*Postn*+ subepicardial mesenchyme and EPDCs (white arrow). (C) ISH of E15.5 heart sections for *Upk3b*, *tdTom*, and *Postn* reveal the expansion of *Postn*+ EPDCs (white arrow). (D) Flow cytometric analysis of E11.5 (n = 31 hearts), E13.5 (n = 25 hearts; 3 independent experiments), E15.5 (n = 10 hearts), and E17.5 hearts (n = 3 hearts; ≥2 independent experiments). The epicardial lineage selected by gating CD31-cTnT-tdTom+, and subsequent analysis of *Sema3d*, *Wt1*, *Tcf21*, *Tbx18*, and *Scx.* TdTom+ cells co-expressing these markers (green and purple) represent the epicardium. Fluorescence minus one (FMO) control and percentage positive shown. (E) Populations expressing *Sema3d*, *Wt1*, *Tcf21*, *Tbx18*, and *Scx*, as a proportion of tdTom+ cells, demonstrate gradual expansion of the EPDC fraction (mean ± SEM; ≥2 independent experiments). (F) Flow cytometric analysis of the proportion of tdTom+ cells during development (E11.5 n = 31; E13.5 n = 25; E15.5 n = 19; E17.5 n = 9; mean ± SEM; 3 independent experiments). RA/LA, right/left atrium; RV/LV, right/left ventricle; myo, myocardium. Scale bars, 200 μm (A); 20 μm (B and C); and 10 μm (inset B and C). See also Figure S5.

Expression of certain epicardial markers, such as *Tcf21*, has been linked to cell fate restriction in EPDCs (Acharya et al., 2012), represented by the tdTom+ cells invading the heart (Figure 5A). To demonstrate the transition to mesenchymal EPDCs, *Upk3b* (epithelial), *Postn* (mesenchymal), and *tdTom* RNAscope probes were multiplexed. At E11.5, *Postn* was largely absent from *tdTom*+ cells, with the exception of the previously described region in the AVG. At E13.5, the appearance of subepicardial mesenchyme and EPDCs was highlighted by the loss of *Upk3b* and acquisition of *Postn* in these cells (Figure 5B). By E15.5, the number of EPDCs present within the heart had significantly increased (Figure 5C).

To determine the marker expression profile of EPDCs, PrimeFlow was performed on tdTom+ hearts at the selected developmental stages. Two different probe combinations were used: *Sema3d/Wt1/Tcf21* and *Sema3d/Scx/Tbx18*, with *Sema3d* serving to ensure comparability between probe sets (Figures 5D and S5A, gating only on tdTom+ cells), and the proportion of tdTom+ cells expressing each marker quantified at every stage (Figure 5E). At E11.5, tdTom+ cells almost exclusively (>95%) represented the epicardium and continued to co-express all marker genes (Figures 5D and 5E). We found no evidence of discrete epicardial sub-populations, based on the degree of overlap or on the absolute levels of marker expression. Any variability in the level of marker expression was similar to that observed for the housekeeping gene *Actb* (Figure S5B), and may reflect fluctuations associated with cell cycle or transcriptional bursts (Corrigan et al., 2016, Padovan-Merhar et al., 2015, Weinreb et al., 2018).

From E13.5, we detected the emergence of EPDCs, as a distinct, additional tdTom+ population in the flow cytometry scatterplots, with decreased expression of *Sema3d*, *Wt1*, *Tbx18*, and *Scx* (Figures 5D and S5A). EPDC number increased as development progressed, to exceed the number of cells within the epicardial layer (Figure 5E); however, the combined number of epicardial cells and EPDCs, as a proportion of total heart cells, remained around 11% (Figure 5F). Although expression of most epicardial markers was downregulated in EPDCs, *Tcf21* expression conversely increased, as revealed by both PrimeFlow and RNAscope (Figures 5D and S5C–S5E). *Tcf21* was maintained in all EPDCs at E13.5 and, from E15.5, was downregulated in a subpopulation, to coincide with the emergence of mural cells (Figures 5D and 5E) (Volz et al., 2015).

### Expression of *Wt1*, *Sema3d*, *Tbx18*, *Scx*, and *Tcf21* Does Not Restrict EPDC Fate

**Figure 6:**
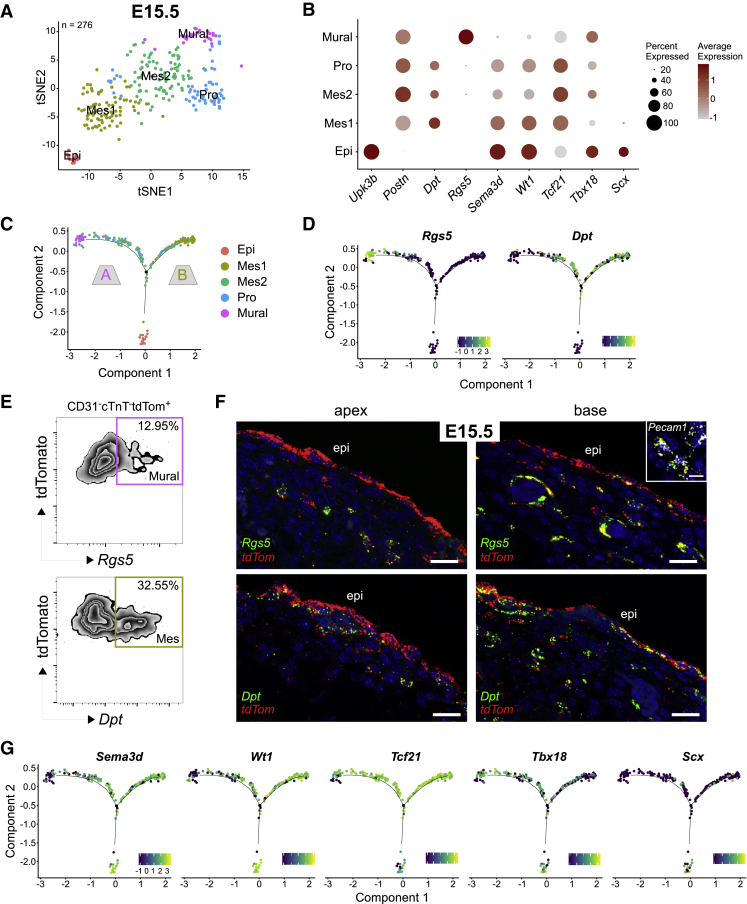
scRNA-Seq Demonstrates Epicardial Contribution of Fibroblasts and Mural Cells but Suggests that Cell Fate Is Not Pre-determined by a Single Epicardial Marker (A) tSNE clustering of epicardial (Epi), mesenchymal (Mes1 and Mes2), mural and proliferating (Pro) cells derived from scRNA-seq tdTom+ cells at E15.5 (total 276 cells; n = 6 pooled hearts). (B) Dot plot showing proportion of cells in each cluster expressing selected genes. Dot size represents percentage of cells expressing, and color scale indicates average expression level. (C) Pseudotime trajectory of tdTom+ cells at E15.5, showing bifurcation to branch A (mural) or branch B (mesenchymal 1). Cells colored by cluster identity. (D) Pseudotime trajectory colored by the expression level of *Rgs5*, representing mural fate, and *Dpt*, indicating mature mesenchymal state. (E) Flow cytometric analysis of E15.5 hearts showing percentage of tdTom+ cells expressing either *Rgs5* or *Dpt*. (F) ISH for *Rgs5*, *Pecam1*, *tdTomato*, and *Dpt* on E15.5 reveals tdTom+ cells expressing *Rgs5*+ neighboring vessels labeled with *Pecam1*. *TdTom*+ *Dpt*+ cells are present near the epicardium, and deeper within the heart. (G) Pseudotime trajectory colored by expression level of selected epicardial genes. Scale bars, 20 μm (F).

To further assess marker expression during EPDC differentiation, and to investigate a link between certain markers and cell fate choice, scRNA-seq was performed on the tdTom+ epicardial lineage FACS sorted from E15.5 hearts, after labeling from the PEO stage, using the SMART-seq2 protocol (Picelli et al., 2014). E15.5 is the embryonic stage when epicardial EMT ceases and EPDCs start differentiating (Liu et al., 2016, Volz et al., 2015). Five transcriptionally distinct populations were identified (Figures 6A and S6A). The first cluster represented the epicardium (Epi), expressing mesothelial genes, such as *Upk3b*. Two mesenchymal clusters were identified: Mes1 possessed a transcriptional profile consistent with the subepicardial mesenchyme, as reported (Xiao et al., 2018), whereas Mes2 was more mature, expressing genes such as *Postn* at higher levels. A proliferating cluster was also identified (Pro), bearing a transcriptional signature similar to Mes2. The fifth cluster represented epicardium-derived mural cells and expressed pericyte-associated genes, such as *Kcnj8* and *Rgs5* (Figure S6A). A dot plot was used to visualize expression of the canonical markers in the different cell populations. *Upk3b* was used to define the epicardial cluster, *Postn* to indicate EPDC state, and *Rgs5* to indicate mural state (Figure 6B). *Sema3d* and *Wt1* were expressed in the entire epicardial cluster (100%; Figures 6B and S6B), whereas their expression was lower in mesenchymal populations and almost undetectable in the mural cluster*. Tcf21* levels were very low in the epicardium at this stage, but high in both mesenchymal clusters. *Tbx18* was mainly localized to the epicardium and the mural cell cluster, while *Scx* was largely confined to the epicardial cluster (Figures 6B and S6B). These data accurately replicate the findings of the RNAscope and PrimeFlow experiments above.

We used Monocle2 to reconstruct the differentiation trajectory of the epicardial lineage in pseudotime. Similar to the epicardial trajectory described previously (Xiao et al., 2018), branch A was associated with expression of mural genes, such as *Pdgfrβ* and *Rgs5*, whereas branch B had higher expression of fibroblast-related genes, such as *Pdgfrα* and *Dpt* (Figures 6C, 6D, and S6C). The branchpoint occurred after mesenchymal cells first emerged, suggesting that EPDCs are initially multipotent. To confirm the scRNA-seq data, PrimeFlow and RNAscope probes were used against *Rgs5* and *Dpt* to investigate mural versus fibroblast fate. At E13.5 these markers were largely absent from the heart (data not shown), suggesting that EPDCs first undergo EMT, then differentiate. At E15.5, *Rgs5* was found in 12.95% of tdTom+ cells, whereas *Dpt* was present in 32.55% of tdTom+ cells (Figure 6E). By E17.5, both *Rgs5* and *Dpt* were much more abundant, showing that EPDCs continue to differentiate as development progresses (Figures S6D and S6E). Intriguingly, whereas *Dpt* was present throughout the myocardial wall, both near the epicardium and deeper inside the heart (Figures 6F and S6E), *Rgs5*+ cells were only found surrounding vessels, suggesting that mural fate may only be specified upon inductive signaling from endothelium and not pre-determined (Figures 6F and S6E). Of significance, none of the epicardial signature markers showed branch-dependent expression, suggesting their expression is insufficient to restrict cell fate (Figure 6G).

### Wt1CreERT2 Labels Coronary Endothelium by E11.5

**Figure 7:**
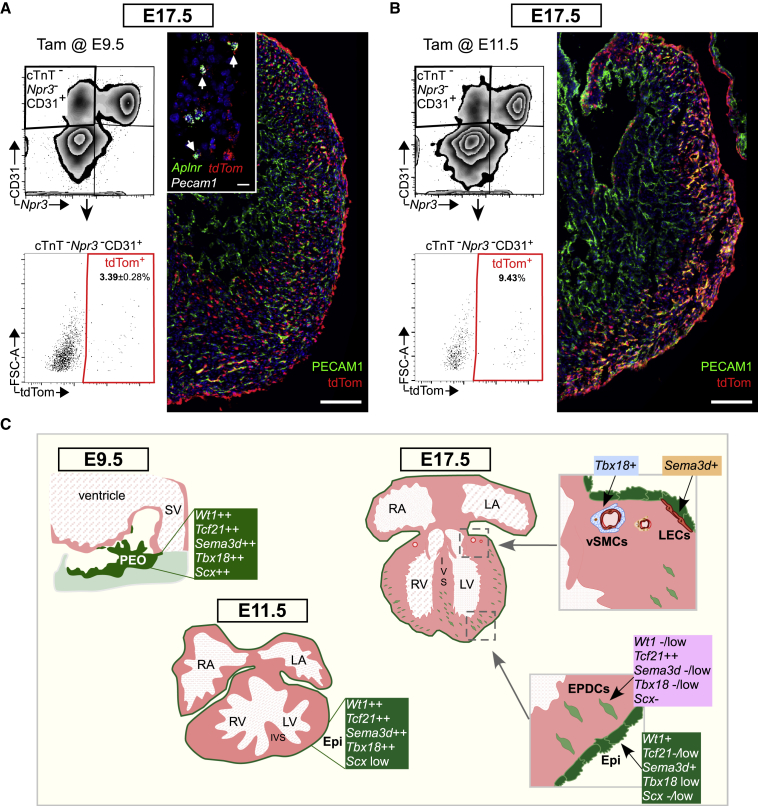
Wt1CreERT2 Targets Coronary Endothelial Cells (A) Flow cytometric analysis of E17.5 hearts induced with tamoxifen (80 mg/kg) at E9.5. Endothelial cells were selected by gating cTnT-Npr3-CD31+ and downstream tdTom+ gating to determine epicardial contribution. Percentage tdTom+ endothelial cells expressed as mean ± SEM (n = 6 hearts). Immunostaining for PECAM1 on E17.5 heart sections reveals tdTom+ PECAM1+ endothelial cells. (inset) ISH for *Aplnr*, *tdTomato*, and *Pecam1* reveals that *tdTom*+ *Pecam1*+ cells are *Aplnr*+, indicating sinus venosus origin. (B) Flow cytometric analysis of E17.5 hearts induced with tamoxifen (80 mg/kg) at E11.5. Endothelial cells were selected by gating cTnT-Npr3-CD31+ and downstream tdTom+ gating to determine epicardial contribution (n = 2 hearts). Immunostaining for PECAM1 on E17.5 heart sections reveals tdTom+ PECAM1+ endothelial cells. (C) Schematic summarizing marker profile of epicardium and its derivatives across the time course of development. Scale bars, 200 μm (A and B) and 20 μm (A inset).

Although scRNA-seq on the epicardial lineage at E15.5 detected no emerging CECs (Figure 6), we sought to more comprehensively evaluate the putative epicardial contribution to the endothelial lineage by assessing later embryonic stages. We performed flow cytometric analysis of the tdTom+ lineage at E17.5, excluding endocardial cells by *Npr3* expression (Zhang et al., 2016). Of the CD31+ *Npr3-*population, tdTom+ cells averaged 3.39% (Figure 7A). However, our previous observation of WT1 expression in CECs as early as E11.5 led us to check for Cre activity 48 h after E9.5 tamoxifen administration. Estrogen receptor α staining revealed nuclear localization of this protein at E11.5 (Figure S7A), indicating that Cre recombinase is still active 48 h after tamoxifen administration. To determine if the 3.39% CEC labeling might result from direct labeling of coronary endothelium, we performed RNAscope against *Aplnr* and *tdTom* at E17.5, since only SV-derived CECs express *Aplnr* (Su et al., 2018). Indeed, the tdTom+ CECs were also positive for *Aplnr*, indicating that they are not epicardial derived (Figures 7A inset and S7B), further supported by our early detection of *Wt1* endogenously in *Aplnr* positive cells at E11.5 (Figure S7C). Since most epicardial studies administer tamoxifen at E11.5 to activate Wt1CreERT2 in the epicardium, we compared the percentage of CECs labeled using this strategy. We found 9.43% of CECs to be labeled with an E11.5 induction, significantly more than administration at E9.5 (Figures 7B and S7D). These percentages may even underestimate the extent of direct CEC labeling since our gating would also include some endothelium of the great vessels and might exclude arterial endothelial cells, which are reported to express *Npr3* (Su et al., 2018). Based on the high level of direct CEC labeling that results from endogenous *Wt1* expression, we conclude that *Wt1*-driven Cre lines cannot be used to assess epicardial contribution to this lineage. However, the complete overlap of five epicardial markers that we demonstrate suggests that the findings of the Tbx18Cre (Cai et al., 2008) and Tcf21CreERT2 (Acharya et al., 2011) studies, indicating no endothelial contribution, may apply to the entire (pro)epicardium.

## Discussion

Collectively, our findings show that PEO cells that transition onto the heart to form the epicardium co-express *Wt1*, *Sema3d*, *Tcf21*, *Scx*, and *Tbx18*, contrasting with a previous report (Figure 7C) (Katz et al., 2012). The disparity may reflect a failure to distinguish STM mesenchyme from PEO and the use of constitutive Cre lines, driven by genes that are not entirely PEO specific, as we and others have highlighted. STM mesenchyme is highly heterogeneous, containing precursors for a multitude of lineages, ranging from CMs (Christoffels et al., 2006) to hematopoietic cells (Cañete et al., 2017). Separating the PEO from the rest of the STM has been challenging, given the lack of molecular boundaries, with markers, such as *Wt1*, *Tbx18*, and *Tcf21* also expressed heterogeneously in some cells of the STM mesenchyme (Cai et al., 2008, Carmona et al., 2016). We found *Upk3b* to be a reliable PEO marker that could be used to define the border between PEO and other progenitors in the STM region. Our findings appear to explain the differential potential of PEO/STM versus epicardial explants, with the former able to give rise to CMs and CECs, which are not present in epicardial outgrowths (Greulich and Kispert, 2013, Red-Horse et al., 2010, Ruiz-Villalba et al., 2013). We hypothesize that endothelial precursors exist in the STM region, and contribute to the heart, but transition via a non-epicardial route, such as circulating endothelial progenitors expressing the STM Cre Tg(G2-*Gata4*), which were recently proposed to contribute via the endocardium (Carmona et al., 2020). Indeed, the PEO/STM markers used to infer CEC contribution, namely *Scx*, *Sema3d*, and *Gata4*, also lineage-label cells in the SV/endocardium (Cano et al., 2016, Katz et al., 2012), suggesting that these precursors may contribute via the main CEC sources.

Assessment of marker expression throughout the heart revealed widespread presence in non-epicardial domains. Of significance, *Tbx18* expression was found in mural cells of both epicardial and non-epicardial origin (aorta and coronary), showing *de novo* expression of this gene in mural cells and supporting results obtained with an inducible Tbx18Cre (Guimaraes-Camboa et al., 2017). We detected SEMA3D expression in cardiac lymphatics, in agreement with reports of SEMA3D expression in lymphatic endothelial cells of the mouse intestine (Jurisic et al., 2012). We were surprised to find expression of WT1 in coronary endothelium as early as E11.5, which means that endothelial labeling cannot be avoided when using Wt1CreERT2, but it can be minimized by delivery of tamoxifen at E9.5, as opposed to E11.5, the latter resulting in significantly greater CEC labeling. Direct endothelial labeling prevents the use of *Wt1*-based lineage tracing to determine epicardial CEC contribution, just as partial overlap of *Scx*, *Sema3d*, and *Gata4* with STM-derived endothelial precursors that traverse via the SV and endocardium precludes their use for this purpose. More selective tools, based, on markers such as *Upk3b,* or another approach, such as intersectional genetics (Pu et al., 2018), will be required to definitively determine epicardial contribution. However, given the ubiquitous expression of *Tbx18* and *Tcf21* throughout the PEO and E10.5 epicardium, and the lack of CEC contribution concluded using Tbx18Cre (Cai et al., 2008) and Tcf21CreERT2 (Acharya et al., 2011), the (pro)epicardium is an unlikely source.

Our scRNA-seq analyses suggest that divergence of epicardial fate occurs after mesenchymal cells first emerge, and that fate may, therefore, be specified in EPDCs after EMT. This model contradicts a previous proposal that fate determination occurred within the epicardial layer (Acharya et al., 2012). Tcf21 was concluded to be required only for EMT of cells destined to become fibroblasts, as Tcf21 null embryos lacked PDGFRα+, but not PDGFRβ+, cells. However, another study reported that Tcf21 null mice fail to form a mature epicardium, preventing EMT altogether (Tandon et al., 2013). The endocardium, an alternative source of coronary mural cells (Chen et al., 2016), may account for the PDGFRβ+ cells present in Tcf21 mutants, and this discrepancy could be resolved by conditional deletion of Tcf21 with Wt1CreERT2 lineage tracing to assess mural cell origin.

Our observation that *Rgs5*+ EPDCs are found only near endothelial cells suggests that mural cell fate may be proximally induced by signaling from the coronary endothelium, with factors, such as PDGF-B, shown to promote mural cell fate of EPDCs (Volz et al., 2015). CECs are the only cell type in the heart that express PDGF-B (Dubé et al., 2017); and, as such, close apposition of EPDCs to CECs may be required for paracrine induction of mural cell maturation.

In conclusion, our data show overlap in expression of canonical markers in the PEO and epicardium early in development, with minimal variation in expression levels between cells. We find no evidence for the existence of distinct sub-compartments in the PEO or epicardial layer. In contrast, other studies have reported heterogeneous Tcf21/Wt1 expression in epicardial cells (Cao et al., 2016, Gambardella et al., 2019, Weinberger et al., 2020). The disparity likely reflects the developmental stages studied and the need to distinguish bona fide epicardial cells, located on the surface of the heart, from EPDCs, which are found below the surface (subepicardial mesenchyme) or within the myocardium (differentiated cells). The studies reporting heterogeneity did not investigate the PEO or the earliest forming epicardium, rather marker expression was evaluated only after initiation of EMT through combined dissociation of cells in both their epithelial and mesenchymal state. With a systematic evaluation over a time course, we demonstrated the conversion of early epicardial Wt1 high/Tcf21 high cells to Wt1 low/Tcf21 high mesenchymal cells (EPDCs) upon EMT and conclude that these distinct cell states reflect a developmental transition, rather than heterogeneity of the starting population. Further expression changes accompany the progression toward quiescence, and downregulation of certain markers, such as Tcf21, appears to reflect quiescent versus active epicardial cell state, rather than pre-determined heterogeneity (Acharya et al., 2012, Braitsch et al., 2012). Studies of epicardial cell cultures are further confounded, since epithelial-mesenchymal status and extent of differentiation are strongly influenced by culture conditions, and spatial information is lacking to corroborate EPDC state. Future studies should incorporate the use of specific epicardial markers, such as *Upk3b*, to distinguish between epicardial state versus epicardium-derived populations, in which the transcriptional signature is altered. We acknowledge that genes outside the tested “epicardial signature” may be heterogeneously expressed; however, we did not observe sub-populations in scRNA-seq datasets. Moreover, based on pseudotime inferred developmental trajectories, we suggest that the fate of EPDCs is specified after EMT, potentially in response to extrinsic cues. Understanding the mechanisms of embryonic fate determination will inform therapeutic strategies to exploit the regenerative potential of epicardial cells.

## Experimental Procedures

### Mouse Strains

Males homozygous for *Rosa26tdTom* (Madisen et al., 2010) and heterozygous for *Wt1CreERT2* (Zhou et al., 2008) were crossed with C57BL/6 females. Pregnant females were oral gavaged with 80 mg/kg tamoxifen at E9.5 or E11.5 (where stated otherwise). All procedures were approved by the University of Oxford Animal Welfare and Ethical Review Board, in accordance with Animals (Scientific Procedures) Act 1986 (Home Office, UK).

### RNAscope

RNAscope Multiplex Fluorescent v.2 assay (ACD) was performed on cryosections according to the manufacturer's instructions, with minor modifications stated in the supplemental material . Probes, including negative control, are detailed in the supplemental material. Probes were optimized for hybridization at 40°C, which permits multiplexing without compromising signal. TSA plus fluorophores was used: fluorescein (1:500), Cy3 (1:1,000), Cy5 (1:1,500).

### PrimeFlow RNA Assay

Enzymatically dissociated hearts were processed using a PrimeFlow RNA Assay Kit (Thermo Fisher Scientific), with staining for cell viability, CD31, WT1, and cardiac troponin T. Probes, detailed in the supplemental material , were used against epicardial markers.

### scRNA-Seq and Analysis

E7.75_E8.25_E9.25 10× Chromium data (GSE126128) was downloaded from UCSC Cell Browser as raw UMI count matrix (de Soysa et al., 2019). Only E9.25 were selected for further analysis. E10.5 heart STRT-seq data were downloaded as TPM from GEO (GSM3027035) (Dong et al., 2018) and E10.5 heart SMART-seq2 data were downloaded as raw counts from GSE76118 (Li et al., 2016). All scRNA-seq datasets were analyzed using Seurat (Butler et al., 2018, Stuart et al., 2018) in R as follows: principal component analysis was used to cluster cells, which were visualized with the tSNE and UMAP method (Becht et al., 2019, Butler et al., 2018). Up to three rounds of clustering iterations were required for larger datasets—E9.25 and E10.5 dataset 1—to separate epicardial cells from: cell types showing similar transcriptomic profile (CM progenitors and STM) and doublets (Epi-CM; Epi-Mes), respectively. For E15.5 scRNA-seq, tamoxifen was administered at E9.5 and E11.5 (40 mg/kg at each stage), ventricles were dissociated and tdTom+ cells sorted for SMART-seq2 on Illumina NextSeq 500 platform. Reads were processed as described in the Supplemental Information and clustering performed as above. Accession Numbers: The accession number for the E15.5 sequencing data reported in this paper (FASTQ files and scaled count matrix available) is GEO: GSE145832. Normalized Seurat data were imported into Monocle2 for pseudotime analysis (Qiu et al., 2017).

Detailed protocols provided in Supplemental Experimental Procedures.

## Author Contributions

Conceptualization, I.-E.L. and N.S.; Experimental Design, I.-E.L., A.N.R., and N.S.; Data Acquisition and Analysis, I.-E.L. and A.N.R.; Writing – Original Draft, I.-E.L.; Writing – Review & Editing, A.N.R. and N.S.; Writing – Figure Preparation, A.N.R.; Bioinformatic Analysis, I.E.-L. and A.N.R.; Supervision, A.N.R. and N.S.; Funding Acquisition, N.S.

## Accession Numbers

E15.5 epicardial lineage scRNA-seq data have been deposited in the GEO data repository under accession number GSE145832.

## Supplemental Information

Document S1. Supplemental Experimental Procedures, Figures S1–S7, and Tables S1–S3

Document S2. Article plus Supplemental Information
