# Supplementary material for: Spatiotemporal Analysis Reveals Overlap of Key Proepicardial Markers in the Developing Murine Heart
Source: Stem Cell Reports. 2020 Apr 30;14(5):770–87. doi: 10.1016/j.stemcr.2020.04.002 (PMC7221110; doi:10.1016/j.stemcr.2020.04.002)
Supplement: Document S1. Supplemental Experimental Procedures, Figures S1–S7, and Tables S1–S3 [file mmc1.pdf]

Stem Cell Reports, Volume *14*

Supplemental Information

Spatiotemporal Analysis Reveals Overlap of Key Proepicardial Markers in the Developing Murine Heart

Irina-Elena Lupu, Andia N. Redpath, and Nicola Smart

Supplemental Information

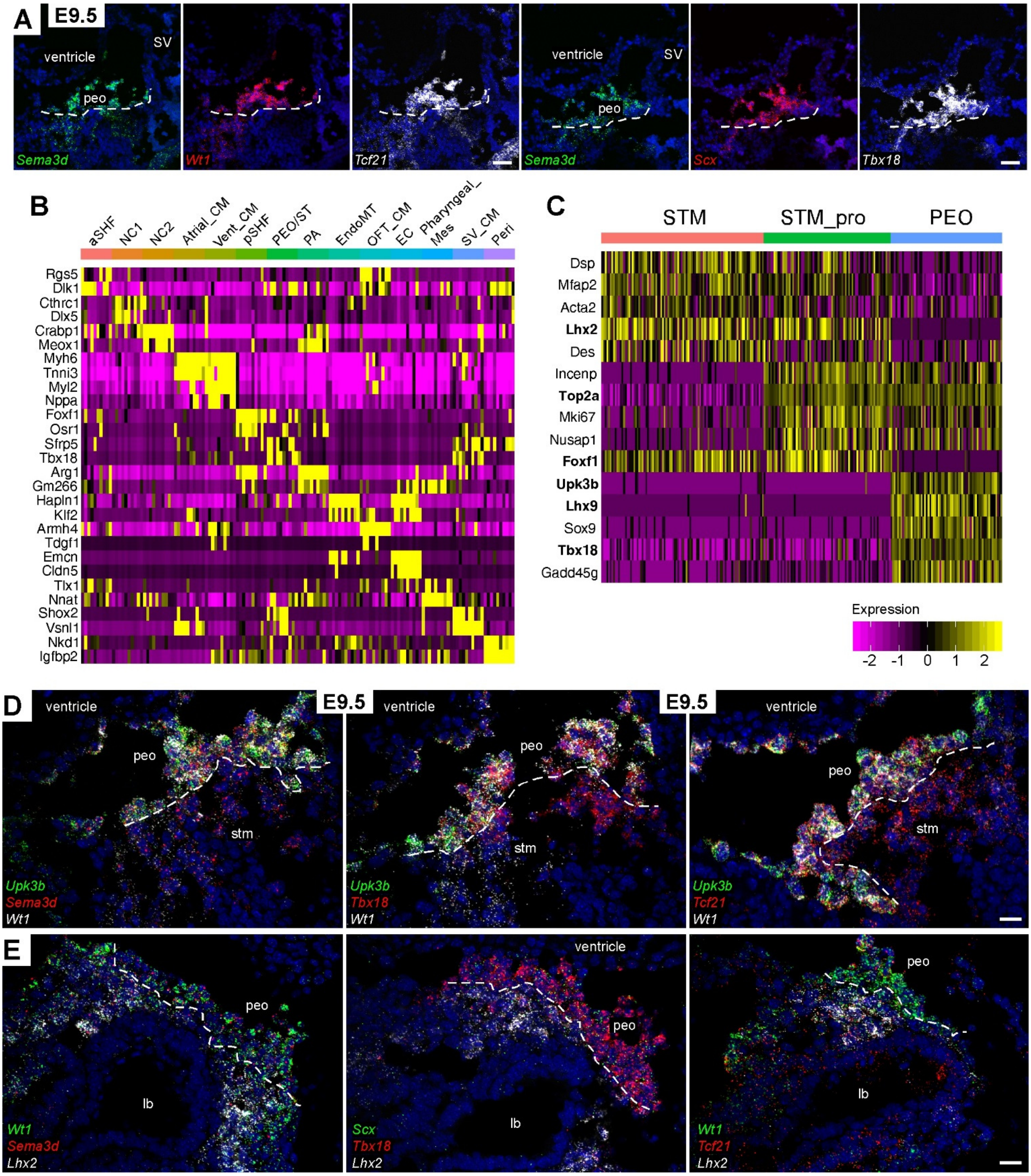

Figure S1. Gene expression of epicardial markers in the septum transversum region and confirmation of E9.25 scRNA-seq cluster identity. Related to Figure 1.

(A) RNA ISH of E9.5 embryo cryosections shows overlapping expression of *Sema3d*, *Wt1*, *Tcf21*, *Scx*, and *Tbx18* mRNA only in the proepicardial organ (peo; dashed line) (n=5 embryos). (B) Heatmap of top 2 differentially expressed genes for each cluster in the E9.25 scRNA-Seq data. High expression is indicated in yellow. (C) Heatmap of top 5 differentially expressed genes for each cluster in the PEO/STM subset. (D) RNA ISH of *Sema3d, Wt1, Tbx18, Tcf21* and *Upk3b* on E9.5 sagittal sections reveals expression of these genes in the proepicardium, demarcated by *Upk3b* (n=3 embryos). (E) RNA ISH of *Sema3d, Wt1, Tbx18, Tcf21, Scx* and *Lhx2*, shows expression of these genes in the septum transversum, demarcated by *Lhx2* (n=3 embryos). *EndoMT, endocardial-to-mesenchymal transition; Endo, endocardial cell; Mes, mesenchyme; PA-pharyngeal arch; aSHF, anterior second heart field; Peri, pericardium; pSHF, posterior second heart field; OFT_CM, outflow tract cardiomyocyte; Vent_CM, ventricular cardiomyocytes; SV_CM, sinus venosus cardiomyocytes; PEO/STM, proepicardium/septum transversum; lb, liver bud.* Scale bars: 50µm A; 20µm D-E.

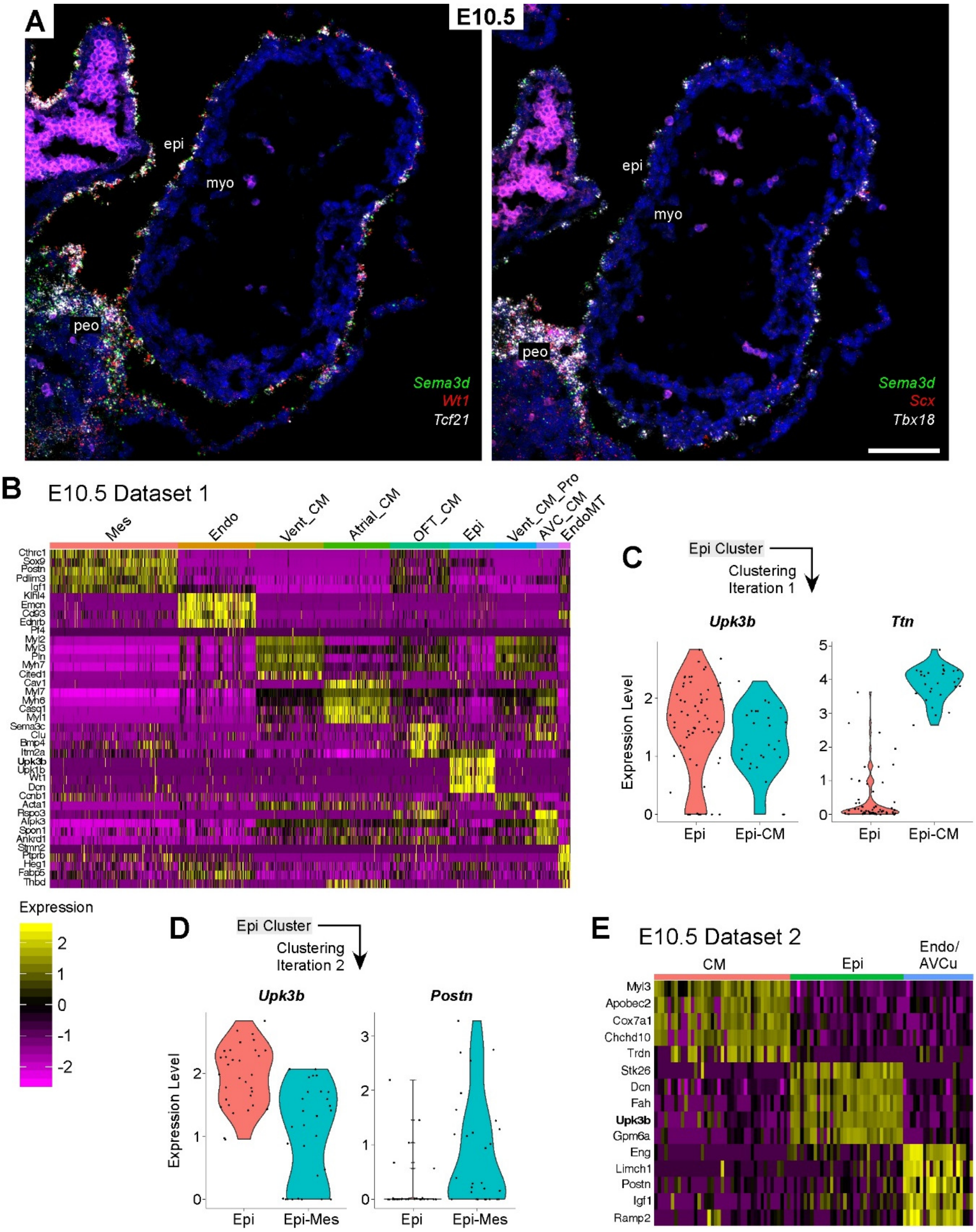

Figure S2. Expression of epicardial markers at E10.5 and confirmation of E10.5 heart scRNA-seq cluster identity. Related to Figure 2.

(A) RNA ISH of E10.5 embryo cryosections shows overlapping expression of *Sema3d*, *Wt1*, *Tcf21*, *Scx*, and *Tbx18* mRNA in the proepicardial organ (peo) and in the epicardium (epi) (n=2 embryos). (B) Heatmap of the top 5 differentially expressed genes for each cluster in E10.5 scRNA-Seq dataset 1. High expression is indicated in yellow. (C) Violin plots showing epicardial-cardiomyocyte (Epi-CM) doublets expressing *Upk3b* and *Ttn* in the first clustering iteration of Epi cluster in B. (D) Violin plots showing epicardial-mesenchymal (Epi-Mes) doublets expressing *Upk3b* and *Postn* in the next clustering iteration of Epi cluster in C. (E) Heatmap of top 5 differentially expressed genes for each cluster in E10.5 scRNA-Seq dataset 2. *peo, proepicardial organ; Vent_CM, ventricular cardiomyocytes, Pro, proliferating; OFT_CM, outflow tract cardiomyocytes; Mes, mesenchyme; EndoMT, endocardial-to-mesenchymal transition; Endo, endocardium; AVC_CM, atrioventricular canal cardiomyocytes; Epi, epicardium, AVCu-atrioventricular cushion.* Scale bars: 100μm A.

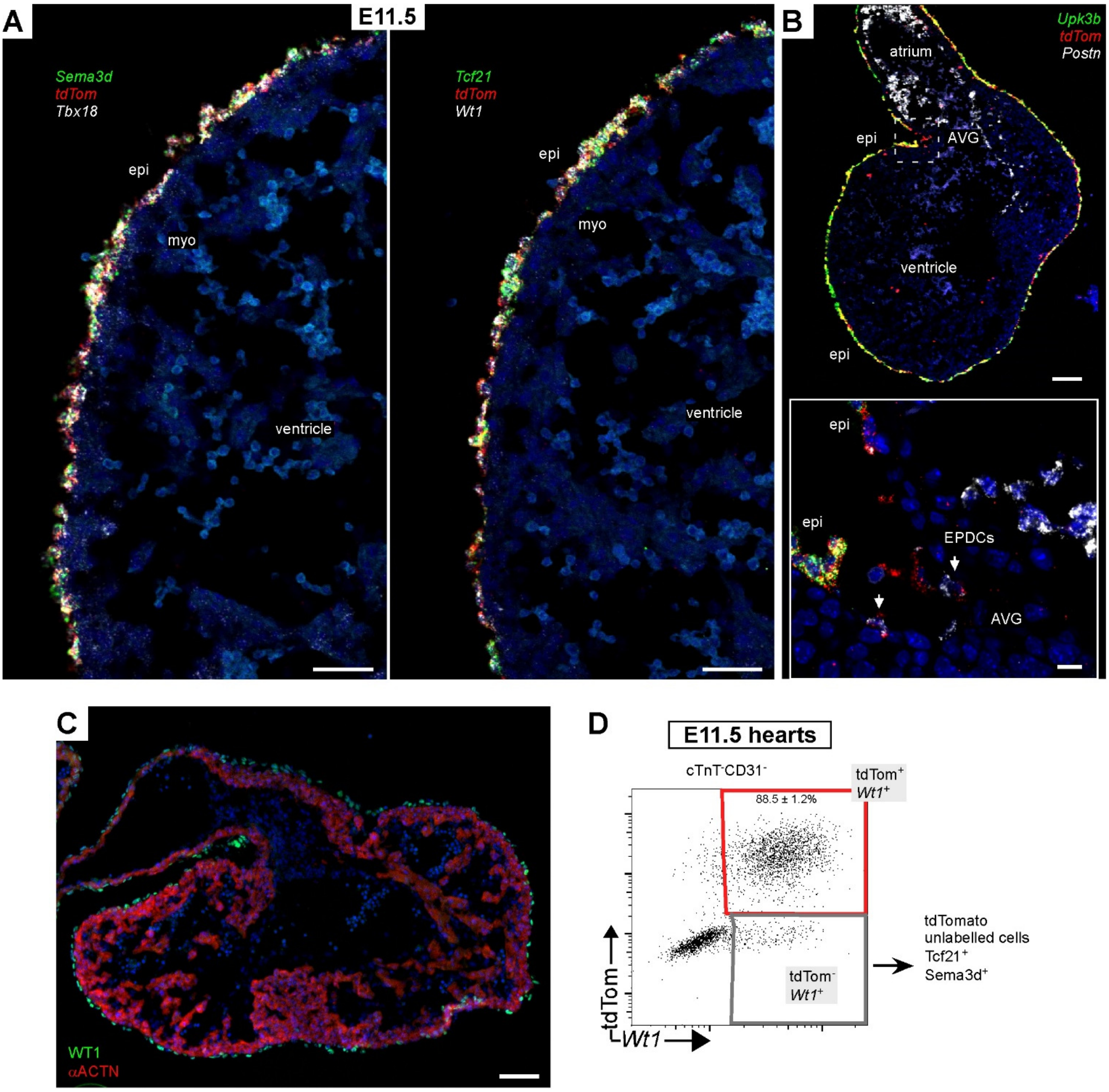

Figure S3. ***Wt1, Sema3d, Tcf21, Tbx18, Scx*** **and** ***Upk3b*** **are expressed throughout the epicardial layer at E11.5. Related to Figure 3.**

(A) RNA ISH on E11.5 heart sections for *Wt1, Sema3d, Tcf21, Tbx18* and *tdTom* reveals overlap of marker genes throughout the epicardial layer (n=4 hearts). (B) RNA ISH for *Upk3b, tdTom* and *Postn* reveals overlap of *Upk3b* and *tdTom* throughout the epicardial layer, with the exception of cells in the atrioventricular groove (AVG) that express *Postn*, indicating mesenchymal state (EPDCs, white arrow; inset) (n=2 hearts). (C) Immunostaining for WT1 and α-sarcomeric-actinin on E11.5 heart sections reveals WT1 expression in all epicardial cells (n=5 hearts). (D) Flow cytometric analysis of *Wt1* transcript and tdTom at E11.5 reveals efficient labelling of epicardial cells by tdTom (mean ± s.e.m; n=29 hearts; ≥3 independent experiments). Scale bars: 50µm A; 100µm B-C; 10µm inset B.

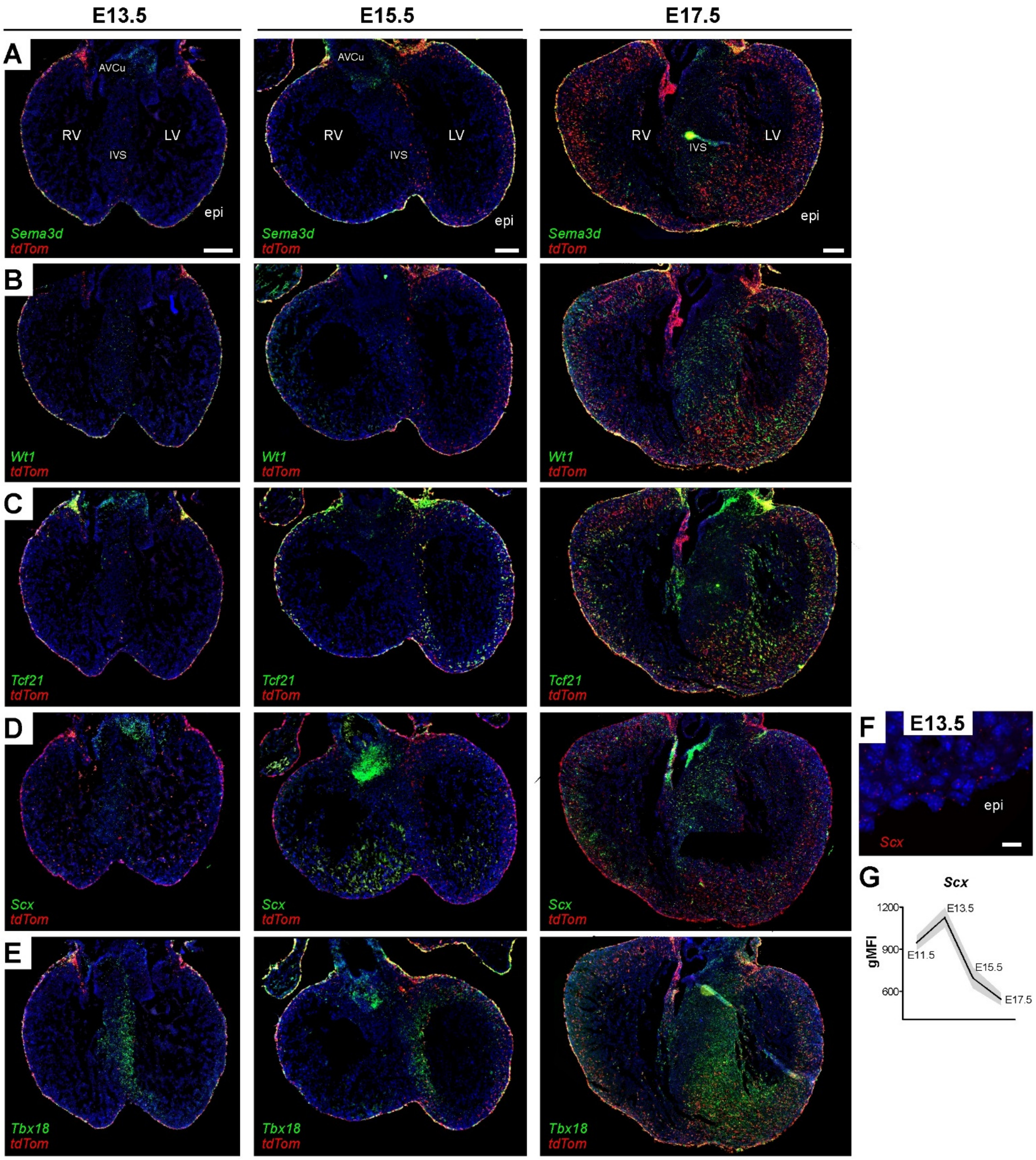

Figure S4. Non-epicardial expression of *Sema3d*, *Wt1*, *Tcf21*, *Scx*, and *Tbx18* at later stages of development. Related to Figure 4.

(A) RNA ISH co-staining of *Sema3d* (green) and *tdTom* (red) at E13.5, E15.5, E17.5 shows overlap in the epicardium. *Sema3d* is also expressed in the atrioventricular cushion (AVCu) and in lymphatic endothelium. (B) RNA ISH co-staining of *Wt1* (green) and *tdTom* (red) at E13.5, E15.5, E17.5 shows overlap in the epicardium. *Wt1* is also expressed in coronary endothelial cells before E13.5. (C) RNA ISH co-staining of *Tcf21* (green) and *tdTom* (red) at E13.5, E15.5, E17.5 shows overlap in the epicardium and in EPDCs. *Tcf21* is also expressed in AVCu and in interstitial fibroblasts. (D) RNA ISH co-staining of *Scx* (green) and *tdTom* (red) at E13.5, E15.5, and E17.5 shows very low expression of Scx in the epicardium. *Scx* is highly expressed in the atrioventricular cushion (AVCu). (E) RNA ISH co-staining of Tbx18 (green) and tdTomato (red) at E13.5, E15.5, E17.5 shows overlap in the epicardium. *Tbx18* is also expressed in cardiomyocytes in the septum and left ventricle and in vascular smooth muscle cells in the aorta (n=3 hearts). (F) RNA ISH of *Scx* at E13.5, showing very low detection of transcripts in the epicardium. (G) Flow cytometric analysis of *Scx* in the epicardial population, demonstrating downregulated expression over development (mean gMFI ± s.e.m; 4 independent experiments). gMFI, geometric Mean Fluorescence

Intensity. AVCu, atrioventricular cushion; RV, right ventricle; IVS, intraventricular septum; LV, left ventricle; epi, epicardium. Scale bars: 200µm A; 10µm F.

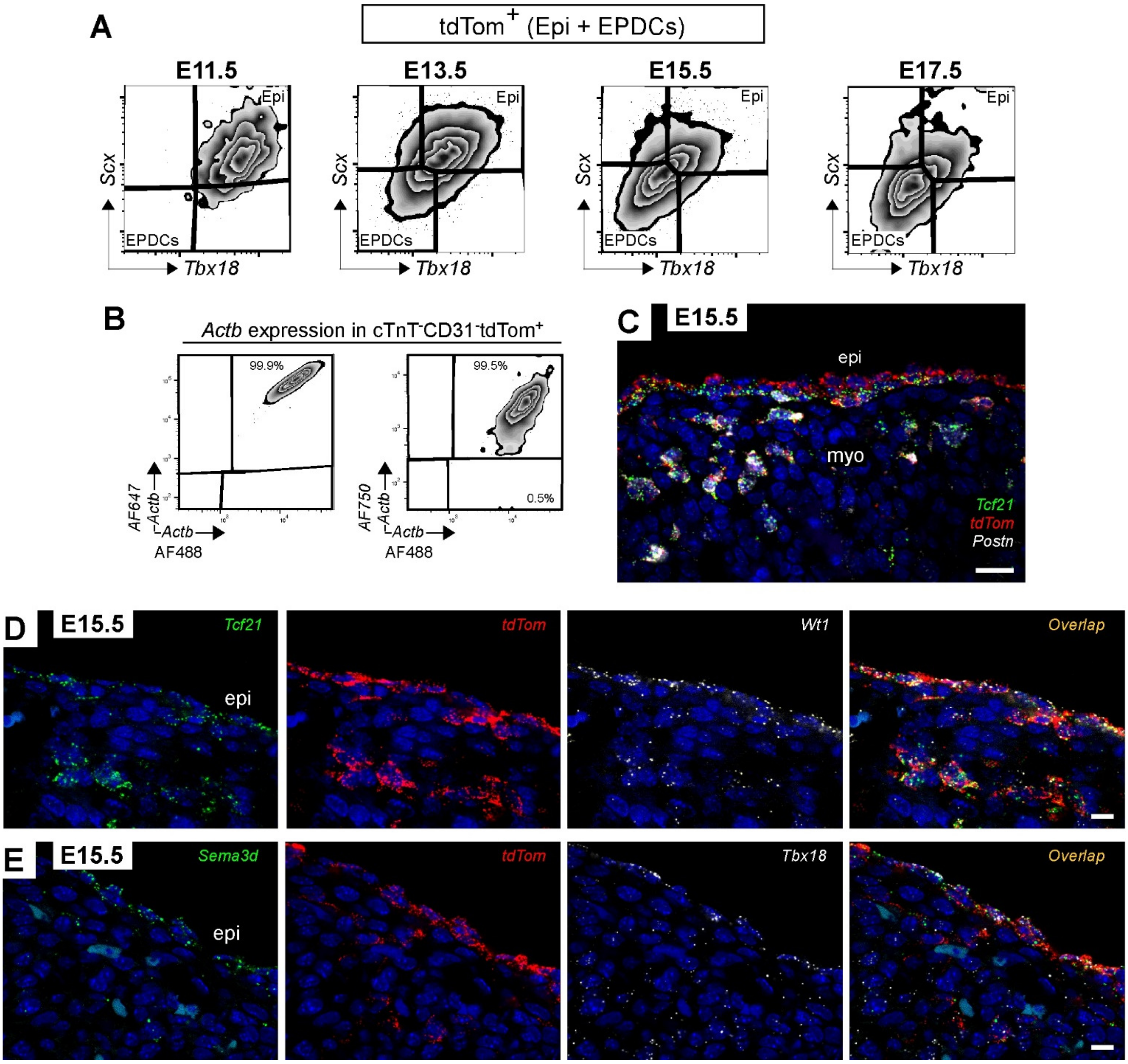

Figure S5. Epicardial marker expression decreases as epicardial cells transition to EPDCs. Related to Figure 5.
(A) Flow cytometric analysis of *Scx* and *Tbx18* expression in tdTom+ cells, representing the epicardial lineage, shows downregulation of these markers in epicardial cells over development (n= as indicated in Figure 5D). (B) Multiplex analysis of *Actb* expression in tdTom+ cells reveals range of expression. (C) RNA ISH for *Tcf21, tdTom* and *Postn* on E15.5 sections reveals increased *Tcf21* expression in mesenchymal cells, demarcated by *Postn* (n=2 hearts). (D) RNA ISH for *Tcf21, tdTom, Wt1,* (E) *Sema3d* and *Tbx18* shows marker expression in the epicardium-derived cells (EPDCs) and epicardium at E15.5. Expression is downregulated in epicardium and EPDCs, with the exception of *Tcf21*, which is downregulated in epicardium but upregulated in EPDCs (n= 3 hearts). Scale bars: 20µm C; 10µm D-E.

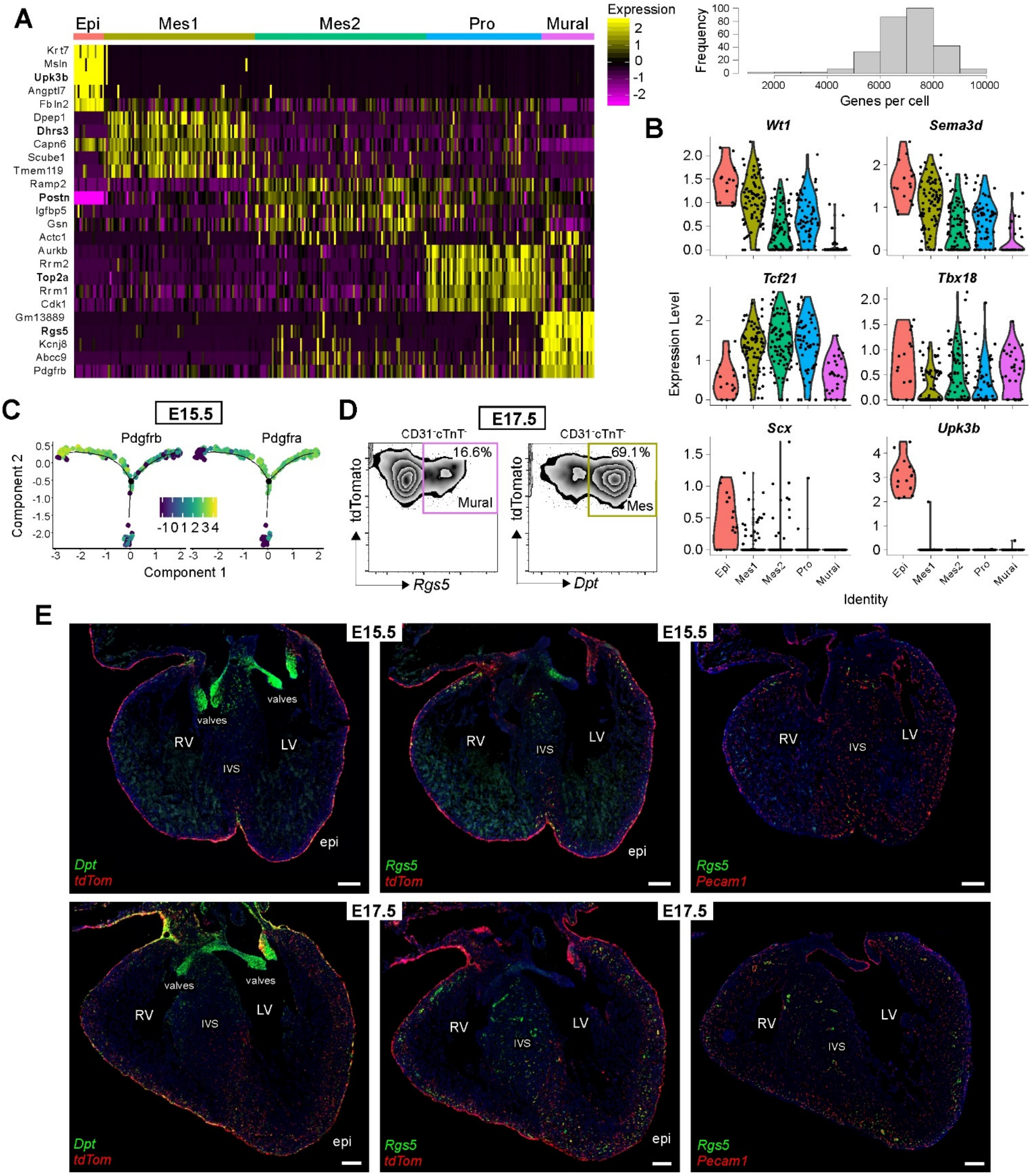

Figure S6. E15.5 scRNA-seq cluster identity, pseudotime representation of cell fate-associated genes and validation of EPDC differentiation. Related to Figure 6.

(A) Heatmap of top 5 differentially expressed genes per cluster in the E15.5 scRNA-Seq. High expression is indicated in yellow. The number of genes per cell captured is shown in the histogram. (B) Violin plots demonstrating expression levels in individuals cells for *Wt1, Sema3d, Tcf21, Tbx18, Scx, and Upk3b,* per cluster in the E15.5 scRNA-Seq. (C) Visualisation of Pdgfr genes in pseudotime reveals two potential cell fates, one associated with mural fate (*Pdgfrβ*) and one associated with mesenchymal/fibroblast fate (*Pdgfrα*). (D) Flow cytometry analysis of E17.5 dissociated hearts reveals percentage of tdTom+ cells, representing EPDCs, positive for *Rgs5* or *Dpt* (mean ± s.e.m; n=6 hearts). (E) RNA ISH for *Dpt, tdTom, Rgs5* and *Pecam1* on E15.5 and E17.5 heart sections reveals appearance of differentiated cells at E15.5 and widespread expression at E17.5 (n=2-3 hearts). Scale bars: 200µm.

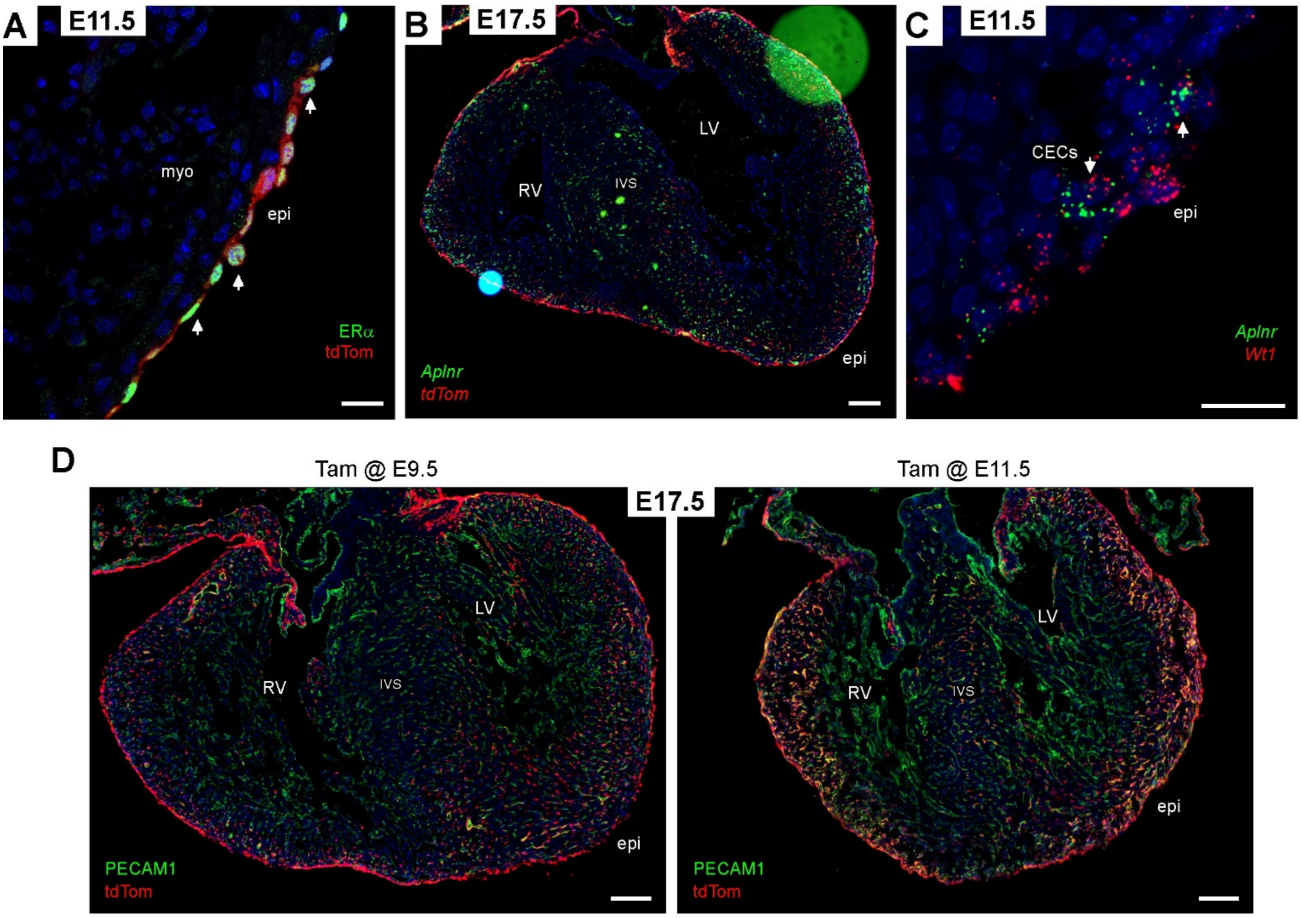

Figure S7. Wt1CreERT2 labels sinus-venosus derived endothelial cells. Related to Figure 7.
(A) Immunostaining for estrogen receptor α (ERα) reveals nuclear localisation at E11.5, indicating that Cre recombinase is active 48h post-tamoxifen administration at E9.5 (white arrows; n=2 hearts). (B) RNA ISH of *Aplnr* and *tdTom* at E17.5, showing expansion of tdTom-labelled SV-derived endothelial cells (n=2 hearts). (C) RNA ISH of *Aplnr* and *Wt1* at E11.5, showing expression of *Wt1* in SV-derived endothelial cells, demarcated by *Aplnr* (n= 1 heart). (D) Immunostaining for PECAM1 on E17.5 heart sections reveals overlap with tdTom+ cells after E9.5 and E11.5 tamoxifen administration (n= 2 hearts). Scale bars: 20µm A,C; 200µm B,D.

Table. S1: % Positive expression (SED) in tdTom+ epicardial population at E11.5

	Sema3d	*Wt1*	*Tcf21*	*Tbx18*	*Scx*
Number of values	3	3	3	3	3
Minimum	97.30	96.00	94.20	94.90	81.60
25% Percentile	97.30	96.00	94.20	94.90	81.60
Median	97.70	98.20	94.90	98.60	90.10
75% Percentile	98.90	98.20	97.40	99.50	94.40
Maximum	98.90	98.20	97.40	99.50	94.40
**Mean**	**97.97**	**97.47**	**95.50**	**97.67**	**88.70**
Std. Deviation	0.8327	1.270	1.682	2.438	6.514
Std. Error	*0.4807*	*0.7333*	*0.9713*	*1.408*	*3.761*

Table. S2: % Positive expression (SED) *Sema3d* in *Wt1*$^{+}$*Tcf21*$^{+}$ epicardial population

	E11.5	E13.5	E15.5	E17.5
Number of values	3	3	2	3
Minimum	98.20	99.50	99.20	96.20
25% Percentile	98.20	99.50	99.20	96.20
Median	98.90	99.70	99.30	96.70
75% Percentile	99.40	99.80	99.40	99.50
Maximum	99.40	99.80	99.40	99.50
**Mean**	**98.83**	**99.67**	**99.30**	**97.47**
Std. Deviation	0.6028	0.1528	0.1414	1.779
Std. Error	*0.3480*	*0.08819*	*0.1000*	*1.027*

Table. S3: % Positive expression (SED) *Scx* in *Tbx18*$^{+}$*Sema3d*$^{+}$ epicardial population

	E11.5	E13.5	E15.5	E17.5
Number of values	3	3	2	3
Minimum	82.80	88.70	97.10	87.90
25% Percentile	82.80	88.70	97.10	87.90
Median	93.60	98.80	97.30	93.20
75% Percentile	94.00	99.00	97.50	96.30
Maximum	94.00	99.00	97.50	96.30
**Mean**	**90.13**	**95.50**	**97.30**	**92.47**
Std. Deviation	6.354	5.890	0.2828	4.248
Std. Error	*3.668*	*3.400*	*0.2000*	*2.452*

**SED, Super-Enhanced Dmax Subtraction algorithm calculates percent positives when comparing histograms

Supplemental Experimental Procedures

Mouse strains. Males homozygous for the *Gt(ROSA)26Sor*$^{tm14(CAG\text{-}tdTomato)Hze}$ allele (Madisen et al., 2010) and heterozygous for *Wt1*$^{tm2(cre/ERT2)Wtp}$ (Zhou et al., 2008) were crossed with C57BL/6 females to generate embryos. The mice used were on a C57BL/6 genetic background. Pregnant females were oral gavaged with 80mg/kg tamoxifen (Sigma, #T5648) 9 days or 11 days (as specified) after the mating plug was detected. Tamoxifen was dissolved in peanut oil with 10% ethanol at a final concentration of 10mg/ml. The animals were kept in a controlled environment and all procedures used were approved by the University of Oxford Animal Welfare and Ethical Review Board in accordance with the Animals (Scientific Procedures) Act 1986 (Home Office, United Kingdom).

RNAscope & Immunofluorescence. RNAscope is a commercially available in-situ hybridization assay provided by Advanced Cell Diagnostics (ACD) (Wang et al., 2012). RNAscope was performed on 8-12 μm thick embryonic cryosections that had been fixed for 2h in 4%PFA and embedded in OCT (Tissue-Tek). The following catalogue probes were used: Wt1 #432711), Sema3d (#488111), Tcf21 (#508661), Tbx18 (#515221), Scx (#439981), tdTomato (#317041), Rgs5 (#430181), Dpt (#561511), Pecam1 (#316721), Aplnr (#436171), Postn (#418581), Upk3b (#823881), Lhx2 (#485791). The 3-plex negative probe against *dapB* was used as a negative control (#320878). The RNAscope Multiplex Fluorescent Reagent Kit v2 assay was performed according to manufacturer's instructions, except for the target retrieval step which was reduced to 10min and the Protease Plus digestion which was performed at room temperature (RT). The TSA plus fluorophores (PerkinElmer, NEL753001KT, NEL745E001KT) were used at 1:500 final concentration for Fluorescein, 1:1000 for Cy3 and 1:1500 for Cy5.

For immunostaining the cryosections were rehydrated for 15 min in PBS, then permeabilised in PBS + 0.5% Triton X-100 for 15 min at RT, followed by blocking in PBS + 10% goat serum +1% bovine serum albumin (BSA) for 1h at RT and incubation with primary antibody diluted in blocking buffer overnight at 4°C. The sections were rinsed three times, then washed three times in PBS + 0.1% Triton X-100, then the secondary antibodies diluted in blocking buffer were added for 1 hour, RT. The sections were rinsed three times in PBS containing 0.1% Triton X-100, then washed twice for 5 min/each wash, followed by 5 min incubation with DAPI nuclear stain. The slides were mounted in Vectashield (Vector Laboratories, H-1000). Antibodies used: WT1 1:100 dilution (Abcam, ab89901), CD31/Pecam1 1:100 dilution (Abcam, ab119341), SEMA3D 1:50 dilution (Abcam, ab198849), no triton/permeabilization, LYVE1 1:100 dilution (eBiosciences, 50-0443-82), sarcomeric a-actinin 1:100 dilution (Abcam; ab9465), Estrogene receptor alpha (ERa) 1:100 dilution (Abcam, ab16660).

Sections were imaged using a Leica DM6000 fluorescence microscope and Olympus Fluoview-1000 confocal microscope and processed using Fiji.

PrimeFlow RNA Assay & Flow Cytometry. Hearts were enzymatically dissociated using the Neonatal Heart Dissociation Kit (#130-098-373) and the gentleMACS Octo Dissociator with heaters (#130-096-427) from Miltenyi Biotec according to manufacturer's instructions. The dissociated hearts were further processed using the PrimeFlow RNA Assay Kit from ThermoFisher Scientific (#88-18005; (Lai et al., 2018)). Briefly, cells were surface stained with the Zombie Aqua Fixable Viability Kit (Biolegend, #423101) for 0.5hr at RT, 1:1000 dilution, followed by BV421 rat anti-CD31 antibody (Biolegend, #102423) for 0.5hr on ice, 1:50 dilution. Samples were further fixed and permeabilised using reagents provided in the kit. Intracellular staining was performed using BV421 mouse anti-cardiac troponin T (cTNT) antibody (BD Biosciences, #565618; 1:200 dilution), and AlexaFluor 488 anti-Wilms Tumor Protein 1 (WT1) antibody (Abcam, ab202635; 1:100 dilution) for 0.5hr on ice, followed by further fixation and hybridization as specified in the instructions. PrimeFlow catalogue probes were used against Scx (#VB1-3028071-PF), Tbx18 (#VB4-3113124-PF), Tcf21 (#VB6-3197774-PF), Sema3d (#VB1-3044712-PF), Wt1 (#VB4-13886-PF), Npr3 (#VB1-3031000-PF), Rgs5 (#VB4-3226783-PF), Dpt (#VB1-3034260-PF). Probes against dapb (#VF1-11712-PF, # VF6-10407-PF, # VF4-10408-PF) were used as negative controls and probes against Actb (#VB6-12823-PF, # VB1-10350-PF, # VB4-10432-PF) as positive controls.

For analysing the contribution of the epicardial lineage to endothelium, hearts were dissociated and surface stained with viability dye as described above, then pre-treated for 5 min on ice with TruStain FcX (anti-mouse CD16/32) antibody (Biolegend, #101319), 1:50 to block Fcγ receptors. Cells were subsequently stained with BV605 rat anti-CD31 antibody, (Biolegend, #102427) on ice for 0.5hr, 1:50 dilution. Cells were fixed and permeabilised using the PrimeFlow kit reagents, then intracellularly stained with cTNT (as described above). Cells were stored in IC fixation buffer (ThermoFisher, #00-8222-49) until acquisition.

All samples were analysed using BD LSRFORTESSA X-20 cytometer and processed using FlowJo software. Percent positive was calculated using Super-Enhanced Dmax Subtraction algorithm (%SED; FlowJo) when comparing histograms (Supplementary Information Table S1-S3). Geometric mean fluorescence intensity (gMFI) was calculated by subtracting fluorescence minus one (FMO) gMFI from sample gMFI.

Single-cell RNA-sequencing & Analysis. E9.25 single cell microdissected embryo data was downloaded as raw UMI matrix from UCSC Cell Browser (de Soysa et al., 2019). Cells with more than 10% mitochondrial reads were filtered out. E10.5 SMART-Seq2 heart data were downloaded as raw counts from Gene Expression Omnibus (GSE76118) (Li et al., 2016). Cells with more than 2% mitochondrial reads and more than 10000 genes per cell were filtered out. E10.5 single cell heart transcriptome data was downloaded as TPM from Gene Expression Omnibus (GSM3027035) (Dong et al., 2018). The TPM

values were log10 transformed and all cells were kept for downstream analysis. All datasets were analysed using Seurat in R (http://www.R-project.org/ (Hafemeister and Satija, 2019; Stuart et al., 2018). In brief, the significant principal components were used to classify the cells into clusters and the tSNE and UMAP methods were used to visualise the clusters (Becht et al., 2019; Butler et al., 2018; Platzer, 2013).

For the E15.5 single-cell RNA-Sequencing data, the ventricles from 6 embryonic hearts were dissociated (as described above) and BD FACSAria III was used to sort cells positive for tdTomato fluorescence into 96-well plates. The cells were processed according to the Smart-Seq2 protocol (Picelli et al., 2014). Tagmentation and library preparation was done using the Nextera XT DNA Library Prep kit and sequenced using the NextSeq 500/550 High Output Kit v2 (75 cycles) 400 million reads (Illumina, #FC-404-2005) on Illumina NextSeq 500 platform. BCL files from the sequencer were converted to FastQ with bcl2fastq (Illumina, v.2.19.1.403) using default settings. Samples with fewer reads in total than the empty wells were discarded. Reads were trimmed using default settings and "--nextera" for adapter clipping (TrimGalore v.0.4.4). Random samples were checked with FastQC (v.0.11.3) to control for the quality of the sequence. Reads were aligned to the mouse genome (GRCm38/Mm10) with STAR (v.2.3.3a) in gene counting mode with Gencode (v.M16) annotations minus the megatranscript Gm20388 (Dobin et al., 2013). Splice junctions from all samples were combined after the 1st pass alignment. Non-canonical junctions and junctions covered with < 10 reads were removed prior to running the 2nd pass alignment. The samples were further processed with Seurat. Genes with > 200 reads in at least 3 cells were retained for downstream processing. Cells were filtered to retain those with < 5% mitochondrial genes and at least 500 expressed genes. 276 cells passed this threshold. Reads were normalized to sequencing depth and scaled. Cell cycle scores (G2M and S) was predicted using G2M and S phase genes (Kowalczyk et al., 2015). The difference between the G2M and S phase score was regressed out using the cell cycle regression Seurat vignette. Clustering was performed as described above. The normalised data from Seurat were imported into Monocle2 for analysis of pseudotime (Qiu et al., 2017; Trapnell et al., 2014).

Supplemental References

Becht, E., McInnes, L., Healy, J., Dutertre, C.-A., Kwok, I.W.H., Ng, L.G., Ginhoux, F., and Newell, E.W. (2019). Dimensionality reduction for visualizing single-cell data using UMAP. Nature Biotechnology *37*, 38-44.

Butler, A., Hoffman, P., Smibert, P., Papalexi, E., and Satija, R. (2018). Integrating single-cell transcriptomic data across different conditions, technologies, and species. Nat Biotechnol *36*, 411-420.

de Soysa, T.Y., Ranade, S.S., Okawa, S., Ravichandran, S., Huang, Y., Salunga, H.T., Schricker, A., del Sol, A., Gifford, C.A., and Srivastava, D. (2019). Single-cell analysis of cardiogenesis reveals basis for organ-level developmental defects. Nature *572*, 120-124.

Dobin, A., Davis, C.A., Schlesinger, F., Drenkow, J., Zaleski, C., Jha, S., Batut, P., Chaisson, M., and Gingeras, T.R. (2013). STAR: ultrafast universal RNA-seq aligner. Bioinformatics (Oxford, England) *29*, 15-21.

Dong, J., Hu, Y., Fan, X., Wu, X., Mao, Y., Hu, B., Guo, H., Wen, L., and Tang, F. (2018). Single-cell RNA-seq analysis unveils a prevalent epithelial/mesenchymal hybrid state during mouse organogenesis. Genome Biol *19*, 31.

Hafemeister, C., and Satija, R. (2019). Normalization and variance stabilization of single-cell RNA-seq data using regularized negative binomial regression. Genome Biology *20*, 296.

Kowalczyk, M.S., Tirosh, I., Heckl, D., Rao, T.N., Dixit, A., Haas, B.J., Schneider, R.K., Wagers, A.J., Ebert, B.L., and Regev, A. (2015). Single-cell RNA-seq reveals changes in cell cycle and differentiation programs upon aging of hematopoietic stem cells. Genome research *25*, 1860-1872.

Lai, C., Stepniak, D., Sias, L., and Funatake, C. (2018). A sensitive flow cytometric method for multi-parametric analysis of microRNA, messenger RNA and protein in single cells. Methods *134-135*, 136-148.

Li, G., Xu, A., Sim, S., Priest, J.R., Tian, X., Khan, T., Quertermous, T., Zhou, B., Tsao, P.S., Quake, S.R.*, et al.* (2016). Transcriptomic Profiling Maps Anatomically Patterned Subpopulations among Single Embryonic Cardiac Cells. Developmental cell *39*, 491-507.

Madisen, L., Zwingman, T.A., Sunkin, S.M., Oh, S.W., Zariwala, H.A., Gu, H., Ng, L.L., Palmiter, R.D., Hawrylycz, M.J., Jones, A.R.*, et al.* (2010). A robust and high-throughput Cre reporting and characterization system for the whole mouse brain. Nature neuroscience *13*, 133-140.

Picelli, S., Faridani, O.R., Björklund, Å.K., Winberg, G., Sagasser, S., and Sandberg, R. (2014). Full-length RNA-seq from single cells using Smart-seq2. Nature Protocols *9*, 171.

Platzer, A. (2013). Visualization of SNPs with t-SNE. PloS one *8*, e56883-e56883.

Qiu, X., Mao, Q., Tang, Y., Wang, L., Chawla, R., Pliner, H.A., and Trapnell, C. (2017). Reversed graph embedding resolves complex single-cell trajectories. Nat Methods *14*, 979-982.

Stuart, T., Butler, A., Hoffman, P., Hafemeister, C., Papalexi, E., Mauck, W.M., Stoeckius, M., Smibert, P., and Satija, R. (2018). Comprehensive integration of single cell data. bioRxiv, 460147.

Trapnell, C., Cacchiarelli, D., Grimsby, J., Pokharel, P., Li, S., Morse, M., Lennon, N.J., Livak, K.J., Mikkelsen, T.S., and Rinn, J.L. (2014). The dynamics and regulators of cell fate decisions are revealed by pseudotemporal ordering of single cells. Nat Biotechnol *32*, 381-386.

Wang, F., Flanagan, J., Su, N., Wang, L.C., Bui, S., Nielson, A., Wu, X., Vo, H.T., Ma, X.J., and Luo, Y. (2012). RNAscope: a novel in situ RNA analysis platform for formalin-fixed, paraffin-embedded tissues. J Mol Diagn *14*, 22-29.

Zhou, B., Ma, Q., Rajagopal, S., Wu, S.M., Domian, I., Rivera-Feliciano, J., Jiang, D., von Gise, A., Ikeda, S., Chien, K.R.*, et al.* (2008). Epicardial progenitors contribute to the cardiomyocyte lineage in the developing heart. Nature *454*, 109-113.
