# Supplementary material for: Spatiotemporal Analysis Reveals Overlap of Key Proepicardial Markers in the Developing Murine Heart
Source: Stem Cell Reports. 2020 Apr 30;14(5):770–87. doi: 10.1016/j.stemcr.2020.04.002 (PMC7221110; doi:10.1016/j.stemcr.2020.04.002)
Supplement: Document S2. Article plus Supplemental Information [file mmc2.pdf]

Stem Cell Reports
Article
OPEN ACCESS

Spatiotemporal Analysis Reveals Overlap of Key Proepicardial Markers in the Developing Murine Heart

Irina-Elena Lupu,[1,2] Andia N. Redpath,[1,2] and Nicola Smart[1,*]
[1]Department of Physiology, Anatomy & Genetics, University of Oxford, Oxford OX1 3PT, UK
[2]Co-first author
*Correspondence: nicola.smart@dpag.ox.ac.uk
https://doi.org/10.1016/j.stemcr.2020.04.002

SUMMARY

The embryonic epicardium, originating from the proepicardial organ (PEO), provides a source of multipotent progenitors for cardiac lineages, including pericytes, fibroblasts, and vascular smooth muscle cells. Maximizing the regenerative capacity of the adult epicardium depends on recapitulating embryonic cell fates. The potential of the epicardium to contribute coronary endothelium is unclear, due to conflicting Cre-based lineage trace data. Controversy also surrounds when epicardial cell fate becomes restricted. Here, we systematically investigate expression of five widely used epicardial markers, *Wt1*, *Tcf21*, *Tbx18*, *Sema3d*, and *Scx*, over the course of development. We show overlap of markers in all PEO and epicardial cells until E13.5, and find no evidence for discrete proepicardial sub-compartments that might contribute coronary endothelium via the epicardial layer. Our findings clarify a number of prevailing discrepancies and support the notion that epicardium-derived cell fate, to form fibroblasts or mural cells, is specified after epithelial-mesenchymal transition, not pre-determined within the PEO.

INTRODUCTION

Recent research has led to a detailed understanding of how the coronary vasculature forms during development, with Cre-*loxP* genetic lineage tracing identifying the sinus venosus and ventricular endocardium as the main contributors of coronary endothelial cells (CECs) (Chen et al., 2014; Red-Horse et al., 2010; Tian et al., 2013, 2014; Zhang et al., 2016). However, a distinct sub-compartment of the proepicardium (PEO) expressing *Sema3d* and *Scx*, largely non-overlapping with the previously described PEO markers *Wt1* and *Tbx18*, was also proposed to give rise to CECs (Katz et al., 2012), raising questions about the vasculogenic potential of epicardial cells. The PEO is a transient embryonic structure that contains epicardial progenitor cells and, in mammals, arises near the septum transversum (STM) from posterior second heart field progenitors (Kruithof et al., 2006; Lie-Venema et al., 2007). PEO cells migrate onto the murine heart surface from embryonic day 9.5 (E9.5) to form the epicardium. Intriguingly, several studies reported ubiquitous expression of *Wt1*, *Tcf21*, and *Tbx18* in the early epicardium (Acharya et al., 2012; Wei et al., 2015), suggesting that these non-overlapping PEO sub-populations may not translate to the epicardium proper. Cre-based lineage tracing, driven by promoters of epicardial genes *Wt1*, *Tbx18*, *Tcf21*, or an enhancer of *Gata5*, reported minimal CEC contribution (Acharya et al., 2012; Cai et al., 2008; Merki et al., 2005; Zhou and Pu, 2012), whereas the discrete PEO sub-compartments, expressing *Sema3d* and/or *Scx*, were reported to contribute: 7% of CECs at E16.5 from the *Sema3d* lineage and 25% of CECs postnatally from the *Scx* lineage (Katz et al., 2012).

Another matter under scrutiny is whether epicardial fate is pre-specified within the PEO or if these cells are multipotent. Epicardial cells undergo epithelial-mesenchymal transition (EMT) from E12.5, giving rise to epicardium-derived cells (EPDCs). Although EPDCs are accepted to differentiate into pericytes, progenitors for coronary vascular smooth muscle cells (vSMCs) (Volz et al., 2015), and cardiac fibroblasts (CFs), it remains unclear what guides their cell fate choice, but *Tcf21* is thought to be a pre-determinant of CF fate (Acharya et al., 2012; Braitsch et al., 2012).

Here, we reveal co-expression of all previously reported markers in the PEO and the entire epicardial layer early in development, finding no support for the putative sub-compartments that might contribute coronary endothelium via the epicardial layer. We also provide evidence to suggest that epicardium-derived cell fate is specified only after EMT, seemingly in response to environmental cues, and importantly marker expression profile—in the PEO or epicardium—does not restrict cell fate choice. Thus, our findings challenge previous concepts around the existence of discrete epicardial sub-populations with pre-determined cell fates.

RESULTS

Wt1, Sema3d, Tbx18, Scx, and Tcf21 Overlap in the PEO, but Their Expression Domains Are Not Confined to This Tissue

First, we used multiplexed single-molecule RNA *in situ* hybridization (RNAscope) on E9.5 sagittal mouse sections to simultaneously detect expression of the PEO markers:

 © 2020 The Author(s).
This is an open access article under the CC BY license (http://creativecommons.org/licenses/by/4.0/).

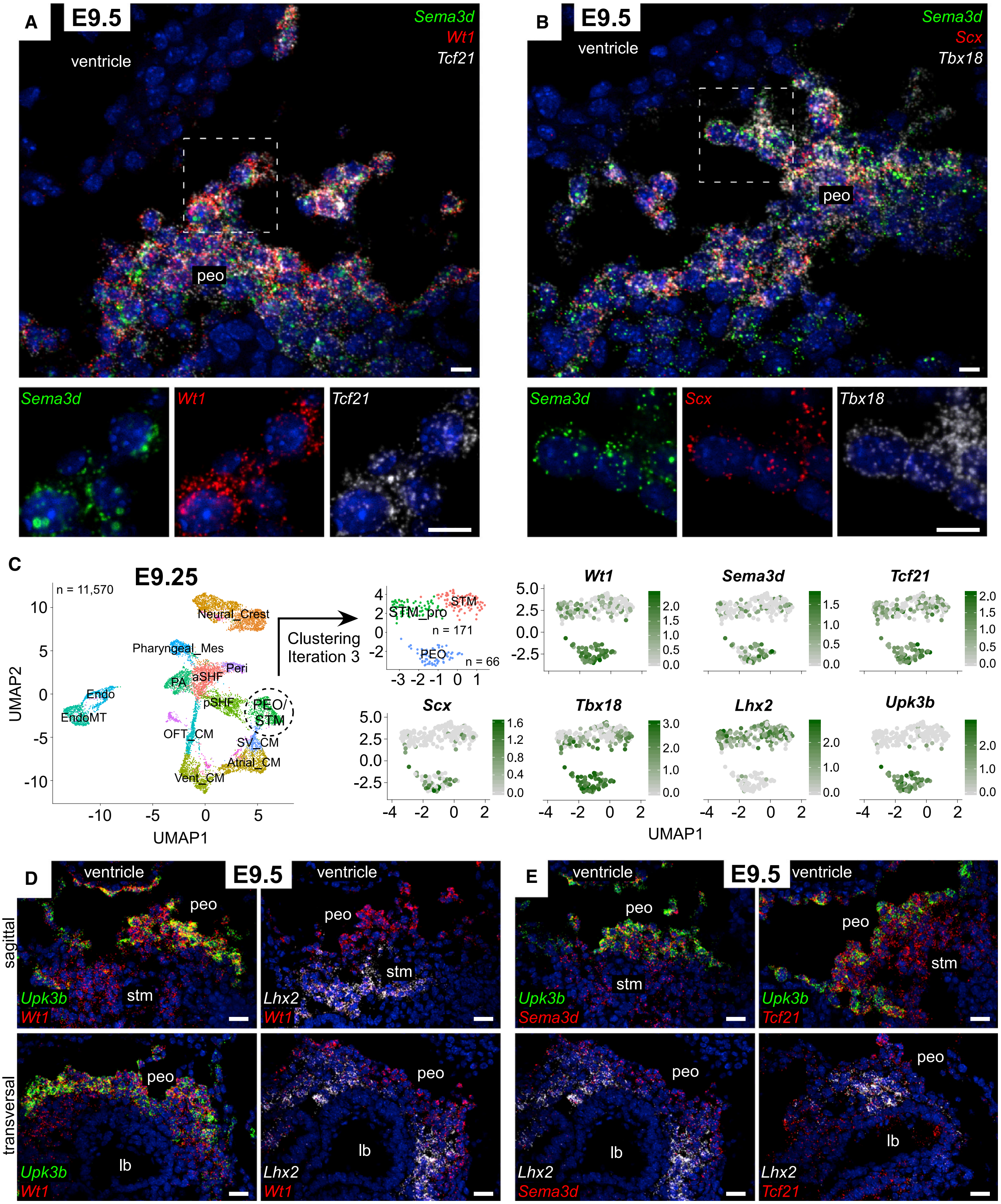

(legend on next page)

Wt1, *Sema3d*, *Tcf21*, *Scx*, and *Tbx18*, having established that signals were highly reproducible, regardless of whether probes were used individually or in combination. We detected complete overlap of marker expression in both the PEO and in cells actively migrating out and onto the heart (Figures 1A and 1B). Morphologically, proepicardial cells are defined as protrusions/villi that extend from the STM region (Maya-Ramos et al., 2013). Our data show that the bona fide PEO ubiquitously co-expresses all tested markers. In contrast, partial overlap was clearly evident below the PEO, in the STM, where distinct expression domains were present (Figure S1A).

To further investigate marker expression in PEO cells, we analyzed published single-cell RNA sequencing (scRNA-seq) data from whole heart and surrounding tissue at E9.25 (de Soysa et al., 2019). Principal component analysis revealed 14 clusters, largely corresponding to neural crest cells, endothelial cells, cardiac progenitor cells, and cardiomyocyte (CM) subsets (Figures 1C and S1B). Initially, PEO and STM cells clustered together due to their similar transcriptomic profiles. Three subsequent clustering iterations separated PEO cells from STM cells, which expressed markers, such as *Lhx2* and *Foxf1* (Kalinichenko et al., 2002; Kolterud et al., 2004; Ren et al., 2014), and included cardiac progenitors positive for *Hand1* (Barnes et al., 2011). The PEO cluster identity was confirmed based on known markers, such as *Lhx9* (Tandon et al., 2016) and mesothelial gene *Upk3b* (Rudat et al., 2014) (Figures 1C and S1C). *Wt1* and *Tbx18* expression was found in 100% of PEO cells, and *Tcf21* was present in 97%. Detection of *Sema3d* and *Scx* was lower, at 70% and 55%, respectively, likely due to limited sensitivity of 10× Chromium technology which only detects highly expressed genes (Baran-Gale et al., 2017). RNAscope, which offers the sensitivity to detect single-molecule RNA, demonstrated expression of all markers throughout the PEO, as shown in our data. The canonical proepicardial genes *Wt1*, *Tcf21*, *Tbx18*, *Sema3d*, and *Scx* were also detected in some cells of the STM cluster, indicating that these genes are not restricted to the PEO (Figure 1C); however, all were enriched in PEO relative to STM. To validate the scRNA-seq data, RNAscope probes against *Upk3b* (PEO marker) and *Lhx2* (STM marker) were used to refine the expression domains of *Wt1*, *Sema3d*, *Tcf21* (Figures 1D, 1E, S1D, and S1E), *Tbx18*, and *Scx* (Figures S1D and S1E) in both sagittal and transversal sections throughout the PEO. The markers completely overlapped with one another in *Upk3b*-expressing cells, demarcating the PEO, but their expression was heterogeneous within the underlying STM, delineated by *Lhx2* expression, consistent with known expression of *Wt1* and *Tcf21* in the hepatic primordium (Lu et al., 2000; Perez-Pomares et al., 2004) and *Tbx18* in CM precursors, located in the STM region (Christoffels et al., 2009).

Epicardial Founder Cells Co-express *Wt1*, *Sema3d*, *Tbx18*, *Scx*, and *Tcf21*

To profile PEO cells that reach the heart and form the definitive epicardium, we multiplexed RNAscope probes on E10.5 sagittal sections. Cells in contact with the heart surface co-expressed all markers, albeit *Scx* was decreased in all cells compared with E9.5 (Figures 2A, 2B, and S2A), as reported previously (Katz et al., 2012). No cells with partially overlapping expression, such as from the underlying STM, were visualized to transition onto the heart. To independently confirm RNAscope findings, we analyzed two published E10.5 mouse heart scRNA-seq datasets (Dong et al., 2018; Li et al., 2016). E10.5 dataset 1 (Li et al., 2016) comprised ten populations, largely corresponding to discrete CM types, endocardial, mesenchymal, and epicardial cells (Figures 2C and S2B). Doublet abundance was evident during our analysis of this dataset, and reported in the original study (Li et al., 2016). Two-thirds of the Epi cluster constituted doublets that were successfully removed after three subsequent clustering iterations (Figures S2C and S2D). E10.5 dataset 2 (Dong et al., 2018) consisted of three populations: epicardium, endocardium/atrioventricular cushion (AVCu), and CMs (Figures 2D and S2E). Across both independent datasets, *Wt1*, *Sema3d*, *Tcf21*, and *Tbx18* were detected in 97%–100% of the epicardial cluster, alongside mesothelial genes, such as *Upk3b* and

Figure 1. *Sema3d*, *Wt1*, *Tcf21*, *Tbx18*, and *Scx* Are Co-expressed in Proepicardial Cells
(A and B) *In situ* hybridization (ISH) of E9.5 embryos for *Sema3d*, *Wt1*, *Tcf21* (A) and *Sema3d*, *Scx*, *Tbx18* (B) mRNA (n = 5).
(C) UMAP plot showing the major clusters in E9.25 scRNA-seq data (total 11,570 cells, n = 2 embryos). UMAP plot showing the PEO and STM cells after three clustering iterations of the PEO/STM cluster. Feature plots representing range of expression of *Wt1*, *Sema3d*, *Tcf21*, *Scx*, *Tbx18*, *Lhx2*, and *Upk3b* in individual cells of the PEO and STM subclusters.
(D) ISH of E9.5 embryos, sagittal and transversal sections, for *Upk3b*, *Wt1*, and *Lhx2*, showing expression of *Wt1* in the PEO and STM (n = 3).
(E) ISH of E9.5 sagittal sections for *Upk3b*, *Sema3d*, and *Tcf21*, and transversal sections for *Lhx2*, *Sema3d*, and *Tcf21*, demonstrating expression of *Sema3d* and *Tcf21* in the PEO and STM, respectively (n = 3).
EndoMT, endocardial-to-mesenchymal transition; Endo, endocardial cells; Mes, mesenchyme; PA, pharyngeal arch; aSHF, anterior second heart field; Peri, pericardium; pSHF, posterior second heart field; OFT_CM, outflow tract cardiomyocytes; Vent_CM, ventricular cardiomyocytes; SV_CM, sinus venosus cardiomyocytes; PEO/STM, proepicardium/septum transversum; lb, liver bud. Scale bars, 10 μm (A and B) and 20 μm (D and E). See also Figure S1.

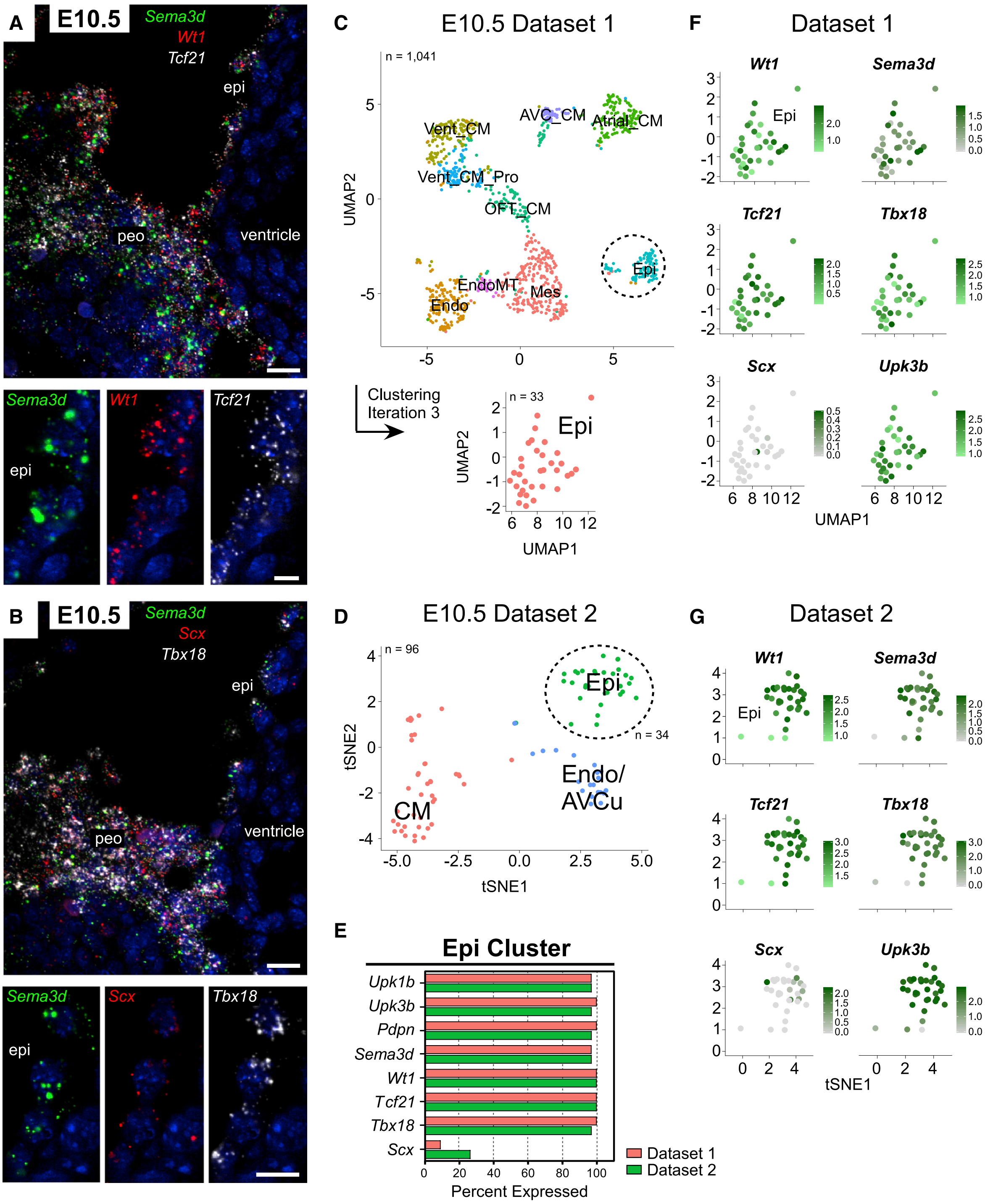

Figure 2. Complete Marker Overlap in Founder Epicardial Cells

(A and B) ISH of E10.5 embryos for *Sema3d*, *Wt1*, *Tcf21* (A) and *Sema3d*, *Scx*, *Tbx18* (B) mRNA (n = 2 embryos).

(C) UMAP plot demonstrating the different major clusters in E10.5 heart scRNA-seq dataset 1 (total 1,041 cells, n = 2 batches). UMAP plot showing the epicardial (Epi) cluster after three clustering iterations to remove doublets.

(D) tSNE plot clustering of cardiomyocytes (CM), endocardium/atrioventricular cushion (Endo/AVCu), and epicardial cells (Epi) in E10.5 heart scRNA-seq dataset 2 (total 96 cells; n = 2 hearts).

(E) Percentage of epicardial cluster expressing selected gene.

(legend continued on next page)

Upk1b (Figure 2E). *Scx* was only detected in a small percentage of cells (Figure 2E), but this likely reflects the limited capture rate of scRNA-seq technology, since *Scx* is expressed at very low levels, with few transcripts per cell evident by RNAscope (Figure 2B). The level of gene expression within individual cells of the epicardial clusters is shown as feature plots (Figures 2F and 2G).

Canonical Epicardial Markers Remain Co-expressed in the E11.5 Epicardium

A key question is how epicardial marker expression changes throughout development, both within the epicardial layer and in the derivative EPDCs as they differentiate. We used the inducible Wt1CreERT2, crossed with R26R-tdTomato (tdTom) reporter, to enable tracing of the epicardial lineage even after epicardial genes are downregulated. As maximal Cre recombination occurs 24–48 h after tamoxifen delivery (Hayashi and McMahon, 2002; Zhou and Pu, 2012), we administered tamoxifen at E9.5 to label the epicardium at E10.5–E11.5 before EMT is initiated (Hayashi and McMahon, 2002; Zhou and Pu, 2012). RNAscope was performed at E11.5, when epicardial formation is complete, multiplexing probes against epicardial markers and *tdTom*. TdTom efficiently labeled the epicardial layer, and inspection of high-power images indicated overlap with all tested markers throughout the epicardial layer (high/low power, Figures 3A–3F; medium power, Figure S3A). As the epicardial layer is non-contiguous in places, even at E11.5, *TdTom* probe was additionally multiplexed with *Upk3b*, an accepted ubiquitous epicardial marker (Rudat et al., 2014; Xiao et al., 2018), to demonstrate efficient labeling of cells in the epicardial layer (Figure S3B). The only domain that did not present overlapping expression of *Upk3b* and *tdTom* was a distinct population of *tdTom*+ cells in the atrioventricular groove (AVG), which were highly positive for *Tcf21* (Figures S3B inset and 3A inset). These cells derived from the *Wt1* lineage, as indicated by *tdTom* expression, but downregulated *Wt1*, *Tbx18*, *Sema3d*, *Scx*, and *Upk3b*, and upregulated *Postn*, indicating mesenchymal state (Figure S3B inset), consistent with the earliest transition to EPDCs in this region (Krainock et al., 2016). Some markers were detected in non-epicardial domains, *Tbx18* in CMs (Figure 3E), *Scx* and *Tcf21* in AVCu (Figures 3D–3F), confirming previous reports (Acharya et al., 2011; Barnette et al., 2014; Christoffels et al., 2009).

A caveat of our study, and others, is the over-reliance on mRNA and reporter readouts due to the inadequacies of available antibodies. To confirm our findings at the protein level, antibodies against WT1 and SEMA3D were used (Figure 3G), while all TBX18, TCF21, and SCX antibodies tested produced non-specific or no staining (not shown). WT1 and SEMA3D proteins were detected uniformly throughout the epicardial layer (Figures 3G and S3C). To confirm correlation between *Wt1* mRNA and WT1 protein levels, and to quantify overlap at the single (whole)-cell level, a flow cytometry-based RNA *in situ* hybridization method was utilized (PrimeFlow RNA Assay) on enzymatically dissociated E11.5 hearts (Figure 3H). Of all cells labeled by *Wt1* probe and/or WT1 antibody, 94% were strongly positive for both (Figure 3I). To assess tdTom labeling efficiency and to exclude the possibility that slight variations in *Wt1* expression might influence recombination, we analyzed overlap of both *Wt1* and WT1 expression with tdTom, which revealed 88.5% of *Wt1*+ cells (Figure S3D) and 89.1% of WT1+ cells to be tdTom labeled (Figure 3I); considered highly efficient for an inducible Cre line (Hayashi and McMahon, 2002). It should be noted that even the non-labeled cells expressed WT1 and other epicardial markers at similar levels to the tdTom-labeled cells, indicating that recombination is stochastic and not correlated with expression level of the endogenous driver (e.g., 97% and 92% of TdTom- *Wt1*+ cells expressed *Sema3d* and *Tcf21*, respectively; Figure S3D). Co-expression of *Wt1*, *Tcf21*, *Sema3d*, and *Tbx18* was detected in >95% of tdTom+ cells (Figure 3J), confirming the RNAscope results. Scx expression was very low and detected only in 88.7% (Figure 3J).

Markers Are Sequentially Downregulated in the Epicardium as Development Progresses

RNAscope and PrimeFlow were used to assess expression of selected markers in the epicardial layer throughout development (Figures 4A and S4A–S4G). In addition to E11.5, three other key developmental stages were chosen for analysis; E13.5, when most EPDCs emerge; E15.5, when EMT is complete; and E17.5, when EPDCs have differentiated. Within the epicardial layer, expression of *Sema3d*, *Wt1*,

(F) Feature plots representing range of expression of *Wt1*, *Sema3d*, *Tcf21*, *Tbx18*, *Scx*, and *Upk3b* in individual cells of dataset 1 epicardial cluster. For genes expressed in 100% of the cluster, expression ranges from light green to dark green, for genes expressed in less than 100%, expression ranges from light gray to dark green.

(G) Feature plots representing range of expression of *Wt1*, *Sema3d*, *Tcf21*, *Tbx18*, *Scx*, and *Upk3b* in individual cells of dataset 2 epicardial cluster.

peo, proepicardial organ; Vent_CM, ventricular cardiomyocytes, Pro, proliferating; OFT_CM, outflow tract cardiomyocytes; Mes, mesenchyme; EndoMT, endocardial-to-mesenchymal transition; Endo, endocardium; AVC_CM, atrioventricular canal cardiomyocytes; Epi, epicardium. Scale bars, 20 μm (A and B low power) and 10 μm (A and B high power). See also Figure S2.

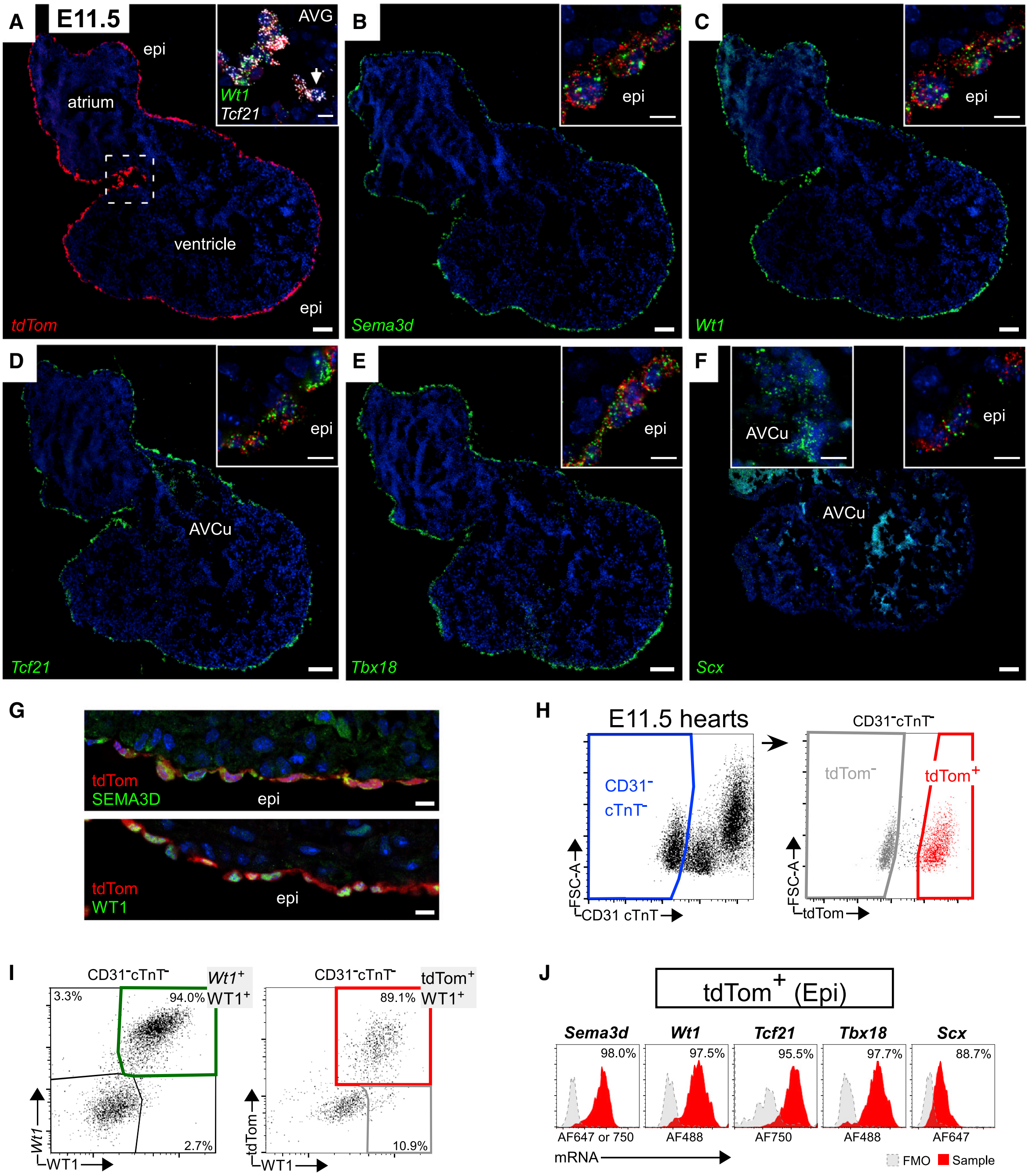

Figure 3. *Sema3d, Wt1, Tcf21, Tbx18,* and *Scx* Are Co-expressed Throughout the Epicardial Layer

(A) ISH of E11.5 hearts shows tdTom labeling of the epicardium (epi), induced at E9.5 in Wt1*CreERT2* embryos (n = 4 hearts). Inset shows cells in atrioventricular groove (AVG) co-expressing *Tcf21* and *tdTom*, and downregulated *Wt1* (arrow).

(B–F) ISH of E11.5 hearts for (B) *Sema3d*, (C) *Wt1*, (D) *Tcf21*, (E) *Tbx18*, and (F) *Scx* with *tdTom*-labeled epicardium (insets) (n = 3 hearts).

(G) Immunostaining of E11.5 heart cryosections for SEMA3D and WT1 reveals expression of SEMA3D and WT1 throughout the epicardial layer, marked by tdTom (n = 3).

(H) Strategy used to gate tdTom+ cells in flow cytometric analysis of E11.5 hearts. Cardiomyocytes and endothelial cells were excluded based on expression of cardiac troponin T (cTnT) and CD31, respectively.

(I) Correlation between *Wt1* transcript and WT1 protein levels (94% overlap) (n = 21 hearts; 3 independent experiments). Correlation between WT1 expression and tdTomato labeling efficiency (89%) (n = 8 hearts; 2 independent experiments).

(legend continued on next page)

Tcf21, and *Tbx18* peaked at E11.5 (Figures 4A and 4B). *Tcf21* expression was dramatically reduced beyond E13.5, followed by a reduction in *Tbx18* expression, to coincide with completion of epicardial EMT by E15.5 and onset of epicardial quiescence (Liu et al., 2016). *Wt1* and *Sema3d* continued to be co-expressed in the entire epicardium throughout development, albeit their levels decreased (Figures 4A, 4B, S4A, and S4B). Although markers were downregulated, their expression remained discernibly homogeneous between individual epicardial cells (Figure 4A) and quantitatively shown to be within a limited dynamic range (Figure 4B).

The specificity of selected markers to the tdTom-labeled epicardial lineage was assessed across the developmental time course (Figures 4C–4F and S4A–S4E). Marker expression increasingly extended to non-epicardial domains by later stages. Surprisingly, WT1 expression was detected in the coronary endothelium as early as E11.5, as revealed by co-staining of WT1 and PECAM1 on sagittal sections (Figure 4C). This was further accentuated by E12.5 (Figure 4C), as previously reported (Duim et al., 2015). *Tbx18* was expressed in vSMCs, both of non-epicardial (in aorta) and epicardial origin (in coronary vessels) (Figure 4D), validating published reports of *de novo Tbx18* expression in most mural cells in the mouse (Guimaraes-Camboa et al., 2017). We also confirmed expression of *Tbx18* in CMs of the left ventricle and IVS (Figure S4E) (Christoffels et al., 2009). *Tcf21* was found in AVG (Acharya et al., 2011; Zhou and Pu, 2012) and in a substantial number of non-epicardium-derived fibroblasts near the endocardial surface (Figures 4E and S4C). Intriguingly, from E16.5, *Sema3d* was expressed in a subset of endothelial cells within the subepicardial space (Figure 4F). Co-staining with LYVE1 showed that SEMA3D is expressed in cardiac lymphatics (not epicardial derived), but not in coronary blood vessel endothelium (Figure 4F). *Sema3d* and *Scx* were also expressed in the AVCu (Figures S4A and S4D), as reported (Katz et al., 2012).

Epicardial Cells Lose Their Marker Signature upon EMT

Expression of certain epicardial markers, such as *Tcf21*, has been linked to cell fate restriction in EPDCs (Acharya et al., 2012), represented by the tdTom+ cells invading the heart (Figure 5A). To demonstrate the transition to mesenchymal EPDCs, *Upk3b* (epithelial), *Postn* (mesenchymal), and *tdTom* RNAscope probes were multiplexed. At E11.5, *Postn* was largely absent from *tdTom+* cells, with the exception of the previously described region in the AVG. At E13.5, the appearance of subepicardial mesenchyme and EPDCs was highlighted by the loss of *Upk3b* and acquisition of *Postn* in these cells (Figure 5B). By E15.5, the number of EPDCs present within the heart had significantly increased (Figure 5C).

To determine the marker expression profile of EPDCs, PrimeFlow was performed on tdTom+ hearts at the selected developmental stages. Two different probe combinations were used: *Sema3d/Wt1/Tcf21* and *Sema3d/Scx/Tbx18*, with *Sema3d* serving to ensure comparability between probe sets (Figures 5D and S5A, gating only on tdTom+ cells), and the proportion of tdTom+ cells expressing each marker quantified at every stage (Figure 5E). At E11.5, tdTom+ cells almost exclusively (>95%) represented the epicardium and continued to co-express all marker genes (Figures 5D and 5E). We found no evidence of discrete epicardial sub-populations, based on the degree of overlap or on the absolute levels of marker expression. Any variability in the level of marker expression was similar to that observed for the housekeeping gene *Actb* (Figure S5B), and may reflect fluctuations associated with cell cycle or transcriptional bursts (Corrigan et al., 2016; Padovan-Merhar et al., 2015; Weinreb et al., 2018).

From E13.5, we detected the emergence of EPDCs, as a distinct, additional tdTom+ population in the flow cytometry scatterplots, with decreased expression of *Sema3d*, *Wt1*, *Tbx18*, and *Scx* (Figures 5D and S5A). EPDC number increased as development progressed, to exceed the number of cells within the epicardial layer (Figure 5E); however, the combined number of epicardial cells and EPDCs, as a proportion of total heart cells, remained around 11% (Figure 5F). Although expression of most epicardial markers was downregulated in EPDCs, *Tcf21* expression conversely increased, as revealed by both PrimeFlow and RNAscope (Figures 5D and S5C–S5E). *Tcf21* was maintained in all EPDCs at E13.5 and, from E15.5, was downregulated in a subpopulation, to coincide with the emergence of mural cells (Figures 5D and 5E) (Volz et al., 2015).

Expression of *Wt1*, *Sema3d*, *Tbx18*, *Scx*, and *Tcf21* Does Not Restrict EPDC Fate

To further assess marker expression during EPDC differentiation, and to investigate a link between certain markers and cell fate choice, scRNA-seq was performed on the tdTom+ epicardial lineage FACS sorted from E15.5 hearts,

(J) Flow cytometric analysis of E11.5 hearts. Epicardial cells, selected by gating CD31-cTnT-tdTom+, show co-expression of *Sema3d*, *Wt1*, *Tcf21*, *Tbx18*, and *Scx* (n = 31 hearts; 3 independent experiments). Fluorescence minus one (FMO) control and percentage positive shown. AVG, atrioventricular groove; AVCu, atrioventricular cushion. Scale bars, 100 μm (A–F); 10 μm (inset A); and 10 μm (inset B–F and G). See also Figure S3.

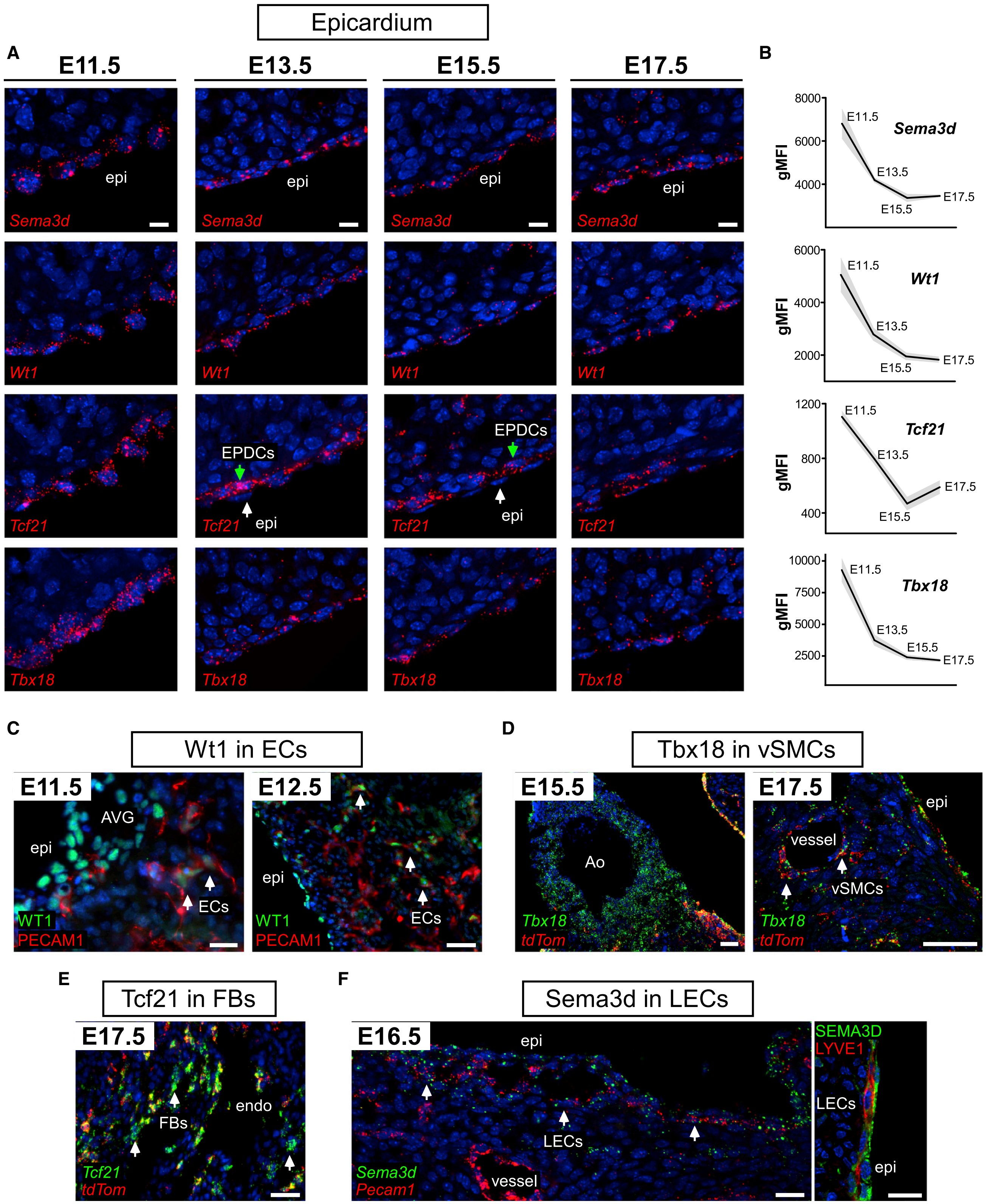

(legend on next page)

after labeling from the PEO stage, using the SMART-seq2 protocol (Picelli et al., 2014). E15.5 is the embryonic stage when epicardial EMT ceases and EPDCs start differentiating (Liu et al., 2016; Volz et al., 2015). Five transcriptionally distinct populations were identified (Figures 6A and S6A). The first cluster represented the epicardium (Epi), expressing mesothelial genes, such as *Upk3b*. Two mesenchymal clusters were identified: Mes1 possessed a transcriptional profile consistent with the subepicardial mesenchyme, as reported (Xiao et al., 2018), whereas Mes2 was more mature, expressing genes such as *Postn* at higher levels. A proliferating cluster was also identified (Pro), bearing a transcriptional signature similar to Mes2. The fifth cluster represented epicardium-derived mural cells and expressed pericyte-associated genes, such as *Kcnj8* and *Rgs5* (Figure S6A). A dot plot was used to visualize expression of the canonical markers in the different cell populations. *Upk3b* was used to define the epicardial cluster, *Postn* to indicate EPDC state, and *Rgs5* to indicate mural state (Figure 6B). *Sema3d* and *Wt1* were expressed in the entire epicardial cluster (100%; Figures 6B and S6B), whereas their expression was lower in mesenchymal populations and almost undetectable in the mural cluster. *Tcf21* levels were very low in the epicardium at this stage, but high in both mesenchymal clusters. *Tbx18* was mainly localized to the epicardium and the mural cell cluster, while *Scx* was largely confined to the epicardial cluster (Figures 6B and S6B). These data accurately replicate the findings of the RNAscope and PrimeFlow experiments above.

We used Monocle2 to reconstruct the differentiation trajectory of the epicardial lineage in pseudotime. Similar to the epicardial trajectory described previously (Xiao et al., 2018), branch A was associated with expression of mural genes, such as *Pdgfrβ* and *Rgs5*, whereas branch B had higher expression of fibroblast-related genes, such as *Pdgfrα* and *Dpt* (Figures 6C, 6D, and S6C). The branchpoint occurred after mesenchymal cells first emerged, suggesting that EPDCs are initially multipotent. To confirm the scRNA-seq data, PrimeFlow and RNAscope probes were used against *Rgs5* and *Dpt* to investigate mural versus fibroblast fate. At E13.5 these markers were largely absent from the heart (data not shown), suggesting that EPDCs first undergo EMT, then differentiate. At E15.5, *Rgs5* was found in 12.95% of tdTom+ cells, whereas *Dpt* was present in 32.55% of tdTom+ cells (Figure 6E). By E17.5, both *Rgs5* and *Dpt* were much more abundant, showing that EPDCs continue to differentiate as development progresses (Figures S6D and S6E). Intriguingly, whereas *Dpt* was present throughout the myocardial wall, both near the epicardium and deeper inside the heart (Figures 6F and S6E), *Rgs5*+ cells were only found surrounding vessels, suggesting that mural fate may only be specified upon inductive signaling from endothelium and not pre-determined (Figures 6F and S6E). Of significance, none of the epicardial signature markers showed branch-dependent expression, suggesting their expression is insufficient to restrict cell fate (Figure 6G).

Wt1CreERT2 Labels Coronary Endothelium by E11.5

Although scRNA-seq on the epicardial lineage at E15.5 detected no emerging CECs (Figure 6), we sought to more comprehensively evaluate the putative epicardial contribution to the endothelial lineage by assessing later embryonic stages. We performed flow cytometric analysis of the tdTom+ lineage at E17.5, excluding endocardial cells by *Npr3* expression (Zhang et al., 2016). Of the CD31+ *Npr3*- population, tdTom+ cells averaged 3.39% (Figure 7A). However, our previous observation of WT1 expression in CECs as early as E11.5 led us to check for Cre activity 48 h after E9.5 tamoxifen administration. Estrogen receptor α staining revealed nuclear localization of this protein at E11.5 (Figure S7A), indicating that Cre recombinase is still active 48 h after tamoxifen administration. To determine if the 3.39% CEC labeling might result from direct labeling of coronary endothelium, we performed RNAscope against *Aplnr* and *tdTom* at E17.5, since only SV-derived CECs express *Aplnr* (Su et al., 2018). Indeed, the tdTom+ CECs were also positive for *Aplnr*, indicating that they are

Figure 4. Epicardial Genes Are Downregulated in the Epicardium as Development Progresses and Become Expressed in Non-epicardial Domains

(A) ISH of E11.5, E13.5, E15.5, and E17.5 hearts show expression of *Sema3d*, *Wt1*, *Tcf21*, *Tbx18*, and *Scx* in the epicardium (n = 3).

(B) Flow cytometric analysis of *Sema3d*, *Wt1*, *Tcf21*, *Tbx18*, and *Scx* in the epicardial population (mean gMFI ± SEM; four independent experiments with exception of Tbx18, n = 2). gMFI, geometric mean fluorescence intensity.

(C) Immunostaining for WT1 and PECAM1 on E11.5 and E12.5 heart sagittal sections reveals expression of WT1 in endothelial cells (ECs).

(D) ISH for *Tbx18* and *tdTom* on E15.5 and E17.5 heart sections reveals expression of *Tbx18* in vSMCs; both in non-epicardium-derived cells in the aorta (Ao) and in epicardium-derived cells in coronary vessels.

(E) ISH for *Tcf21* and *tdTomo* on E17.5 heart sections reveals high *Tcf21* expression in fibroblasts of non-epicardial origin, near the endocardial surface.

(F) ISH for *Sema3d* and *Pecam1* and immunostaining for SEMA3D and LYVE1 reveals SEMA3D expression in cardiac lymphatic endothelial cells (LECs) in E16.5 heart sections.

Scale bars, 10 μm (A and C) E11.5; 50 μm (C) E12.5 and (D); and 20 μm (E and F). See also Figure S4.

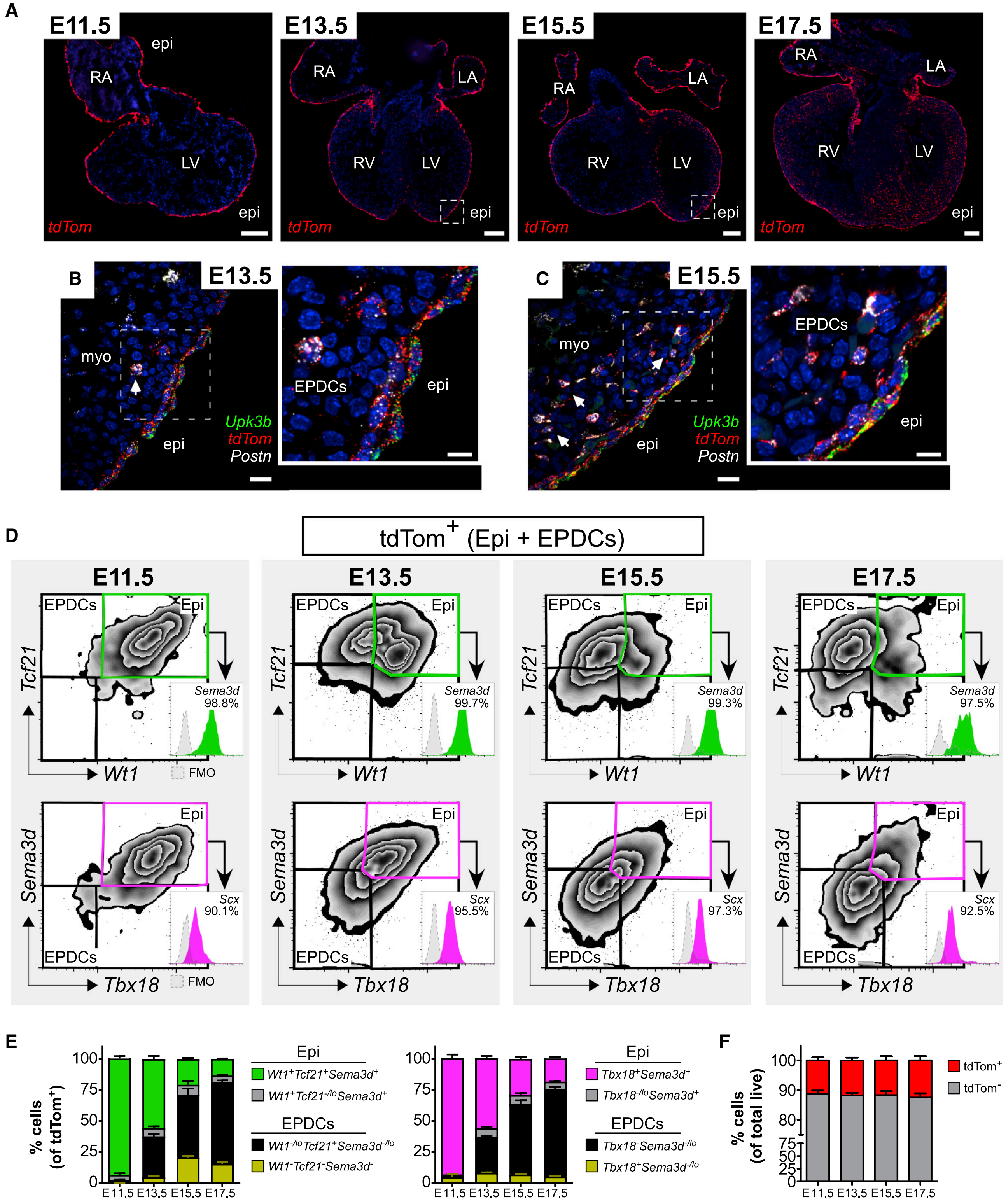

Figure 5. Epicardium-Derived Progenitors Lose Epicardial Signature

(A) ISH of E11.5, E13.5, E15.5, and E17.5 hearts shows tdTom labeling of the epicardium and lineage-traced EPDCs (n = 3 hearts/stage). Boxed regions indicate magnified regions in (B and C).

(legend continued on next page)

not epicardial derived (Figures 7A inset and S7B), further supported by our early detection of *Wt1* endogenously in *Aplnr* positive cells at E11.5 (Figure S7C). Since most epicardial studies administer tamoxifen at E11.5 to activate Wt1CreERT2 in the epicardium, we compared the percentage of CECs labeled using this strategy. We found 9.43% of CECs to be labeled with an E11.5 induction, significantly more than administration at E9.5 (Figures 7B and S7D). These percentages may even underestimate the extent of direct CEC labeling since our gating would also include some endothelium of the great vessels and might exclude arterial endothelial cells, which are reported to express *Npr3* (Su et al., 2018). Based on the high level of direct CEC labeling that results from endogenous *Wt1* expression, we conclude that *Wt1*-driven Cre lines cannot be used to assess epicardial contribution to this lineage. However, the complete overlap of five epicardial markers that we demonstrate suggests that the findings of the Tbx18Cre (Cai et al., 2008) and Tcf21CreERT2 (Acharya et al., 2011) studies, indicating no endothelial contribution, may apply to the entire (pro)epicardium.

DISCUSSION

Collectively, our findings show that PEO cells that transition onto the heart to form the epicardium co-express *Wt1*, *Sema3d*, *Tcf21*, *Scx*, and *Tbx18*, contrasting with a previous report (Figure 7C) (Katz et al., 2012). The disparity may reflect a failure to distinguish STM mesenchyme from PEO and the use of constitutive Cre lines, driven by genes that are not entirely PEO specific, as we and others have highlighted. STM mesenchyme is highly heterogeneous, containing precursors for a multitude of lineages, ranging from CMs (Christoffels et al., 2006) to hematopoietic cells (Cañete et al., 2017). Separating the PEO from the rest of the STM has been challenging, given the lack of molecular boundaries, with markers, such as *Wt1*, *Tbx18*, and *Tcf21* also expressed heterogeneously in some cells of the STM mesenchyme (Cai et al., 2008; Carmona et al., 2016). We found *Upk3b* to be a reliable PEO marker that could be used to define the border between PEO and other progenitors in the STM region. Our findings appear to explain the differential potential of PEO/STM versus epicardial explants, with the former able to give rise to CMs and CECs, which are not present in epicardial outgrowths (Greulich and Kispert, 2013; Red-Horse et al., 2010; Ruiz-Villalba et al., 2013). We hypothesize that endothelial precursors exist in the STM region, and contribute to the heart, but transition via a non-epicardial route, such as circulating endothelial progenitors expressing the STM Cre Tg(G2-*Gata4*), which were recently proposed to contribute via the endocardium (Carmona et al., 2020). Indeed, the PEO/STM markers used to infer CEC contribution, namely *Scx*, *Sema3d*, and *Gata4*, also lineage-label cells in the SV/endocardium (Cano et al., 2016; Katz et al., 2012), suggesting that these precursors may contribute via the main CEC sources.

Assessment of marker expression throughout the heart revealed widespread presence in non-epicardial domains. Of significance, *Tbx18* expression was found in mural cells of both epicardial and non-epicardial origin (aorta and coronary), showing *de novo* expression of this gene in mural cells and supporting results obtained with an inducible Tbx18Cre (Guimaraes-Camboa et al., 2017). We detected SEMA3D expression in cardiac lymphatics, in agreement with reports of SEMA3D expression in lymphatic endothelial cells of the mouse intestine (Jurisic et al., 2012). We were surprised to find expression of WT1 in coronary endothelium as early as E11.5, which means that endothelial labeling cannot be avoided when using Wt1CreERT2, but it can be minimized by delivery of tamoxifen at E9.5, as opposed to E11.5, the latter resulting in significantly greater CEC labeling. Direct endothelial labeling prevents the use of *Wt1*-based lineage tracing to determine epicardial CEC contribution, just as partial overlap of *Scx*, *Sema3d*, and *Gata4* with STM-derived endothelial precursors that traverse via the SV and

(B) ISH of E13.5 heart sections for *Upk3b*, *tdTom*, and *Postn* reveal the appearance of *Upk3b*-*Postn*+ subepicardial mesenchyme and EPDCs (white arrow).

(C) ISH of E15.5 heart sections for *Upk3b*, *tdTom*, and *Postn* reveal the expansion of *Postn*+ EPDCs (white arrow).

(D) Flow cytometric analysis of E11.5 (n = 31 hearts), E13.5 (n = 25 hearts; 3 independent experiments), E15.5 (n = 10 hearts), and E17.5 hearts (n = 3 hearts; $\geq$2 independent experiments). The epicardial lineage selected by gating CD31-cTnT-tdTom+, and subsequent analysis of *Sema3d*, *Wt1*, *Tcf21*, *Tbx18*, and *Scx*. TdTom+ cells co-expressing these markers (green and purple) represent the epicardium. Fluorescence minus one (FMO) control and percentage positive shown.

(E) Populations expressing *Sema3d*, *Wt1*, *Tcf21*, *Tbx18*, and *Scx*, as a proportion of tdTom+ cells, demonstrate gradual expansion of the EPDC fraction (mean $\pm$ SEM; $\geq$2 independent experiments).

(F) Flow cytometric analysis of the proportion of tdTom+ cells during development (E11.5 n = 31; E13.5 n = 25; E15.5 n = 19; E17.5 n = 9; mean $\pm$ SEM; 3 independent experiments).

RA/LA, right/left atrium; RV/LV, right/left ventricle; myo, myocardium. Scale bars, 200 μm (A); 20 μm (B and C); and 10 μm (inset B and C). See also Figure S5.

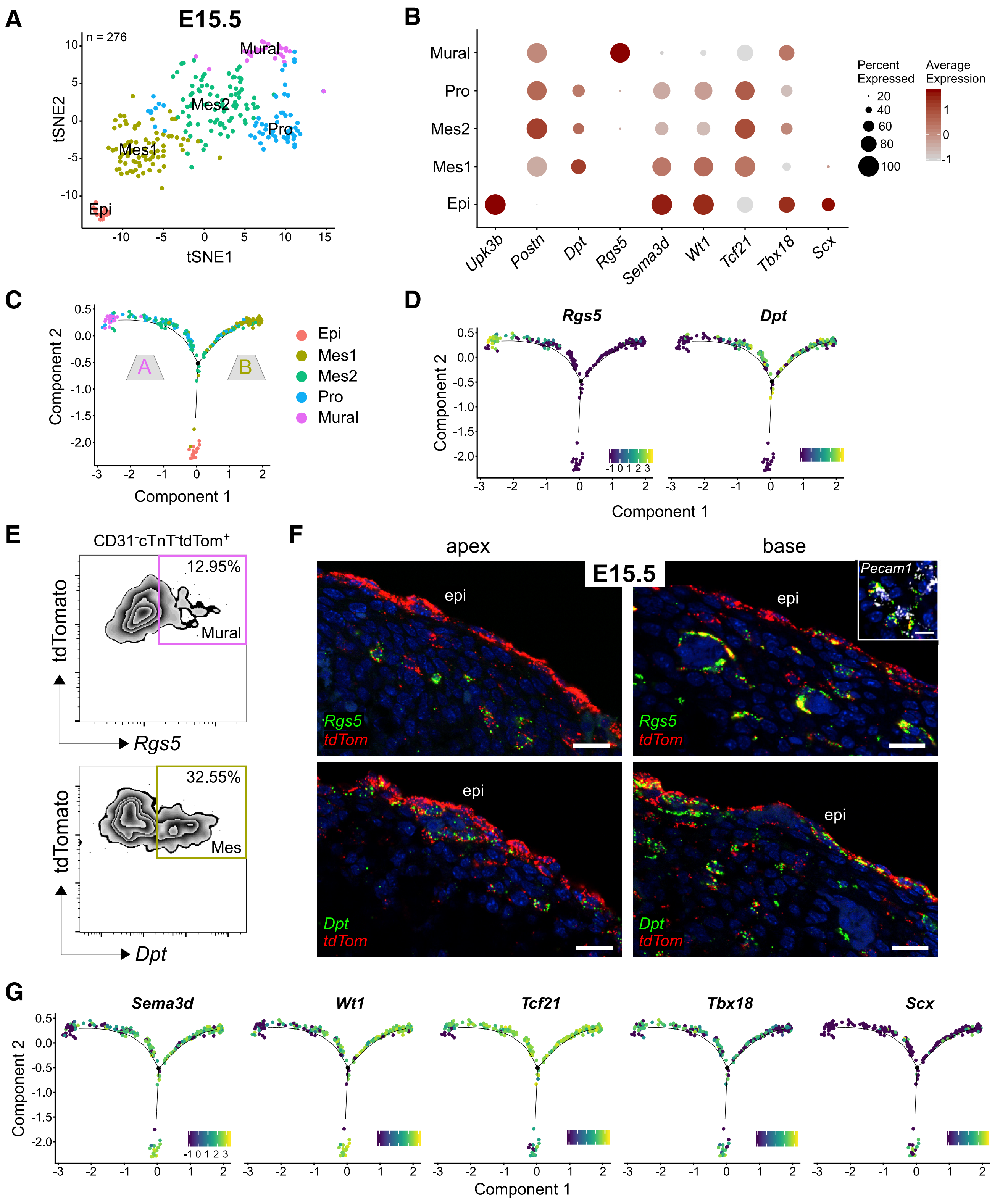

(legend on next page)

endocardium precludes their use for this purpose. More selective tools, based, on markers such as *Upk3b,* or another approach, such as intersectional genetics (Pu et al., 2018), will be required to definitively determine epicardial contribution. However, given the ubiquitous expression of *Tbx18* and *Tcf21* throughout the PEO and E10.5 epicardium, and the lack of CEC contribution concluded using Tbx18Cre (Cai et al., 2008) and Tcf21CreERT2 (Acharya et al., 2011), the (pro)epicardium is an unlikely source.

Our scRNA-seq analyses suggest that divergence of epicardial fate occurs after mesenchymal cells first emerge, and that fate may, therefore, be specified in EPDCs after EMT. This model contradicts a previous proposal that fate determination occurred within the epicardial layer (Acharya et al., 2012). Tcf21 was concluded to be required only for EMT of cells destined to become fibroblasts, as Tcf21 null embryos lacked PDGFRα+, but not PDGFRβ+, cells. However, another study reported that Tcf21 null mice fail to form a mature epicardium, preventing EMT altogether (Tandon et al., 2013). The endocardium, an alternative source of coronary mural cells (Chen et al., 2016), may account for the PDGFRβ+ cells present in Tcf21 mutants, and this discrepancy could be resolved by conditional deletion of Tcf21 with Wt1CreERT2 lineage tracing to assess mural cell origin.

Our observation that *Rgs5*+ EPDCs are found only near endothelial cells suggests that mural cell fate may be proximally induced by signaling from the coronary endothelium, with factors, such as PDGF-B, shown to promote mural cell fate of EPDCs (Volz et al., 2015). CECs are the only cell type in the heart that express PDGF-B (Dubé et al., 2017); and, as such, close apposition of EPDCs to CECs may be required for paracrine induction of mural cell maturation.

In conclusion, our data show overlap in expression of canonical markers in the PEO and epicardium early in development, with minimal variation in expression levels between cells. We find no evidence for the existence of distinct sub-compartments in the PEO or epicardial layer. In contrast, other studies have reported heterogeneous Tcf21/Wt1 expression in epicardial cells (Cao et al., 2016; Gambardella et al., 2019; Weinberger et al., 2020). The disparity likely reflects the developmental stages studied and the need to distinguish bona fide epicardial cells, located on the surface of the heart, from EPDCs, which are found below the surface (subepicardial mesenchyme) or within the myocardium (differentiated cells). The studies reporting heterogeneity did not investigate the PEO or the earliest forming epicardium, rather marker expression was evaluated only after initiation of EMT through combined dissociation of cells in both their epithelial and mesenchymal state. With a systematic evaluation over a time course, we demonstrated the conversion of early epicardial Wt1 high/Tcf21 high cells to Wt1 low/Tcf21 high mesenchymal cells (EPDCs) upon EMT and conclude that these distinct cell states reflect a developmental transition, rather than heterogeneity of the starting population. Further expression changes accompany the progression toward quiescence, and downregulation of certain markers, such as Tcf21, appears to reflect quiescent versus active epicardial cell state, rather than pre-determined heterogeneity (Acharya et al., 2012; Braitsch et al., 2012). Studies of epicardial cell cultures are further confounded, since epithelial-mesenchymal status and extent of differentiation are strongly influenced by culture conditions, and spatial information is lacking to corroborate EPDC state. Future studies should incorporate the use of specific epicardial markers, such as *Upk3b*, to distinguish between epicardial state versus epicardium-derived populations, in which the transcriptional signature is altered. We acknowledge that genes outside the tested "epicardial signature" may be heterogeneously expressed; however, we did not observe sub-populations in scRNA-seq datasets. Moreover, based on pseudotime inferred developmental trajectories, we suggest that the fate of EPDCs is specified

Figure 6. scRNA-Seq Demonstrates Epicardial Contribution of Fibroblasts and Mural Cells but Suggests that Cell Fate Is Not Predetermined by a Single Epicardial Marker

(A) tSNE clustering of epicardial (Epi), mesenchymal (Mes1 and Mes2), mural and proliferating (Pro) cells derived from scRNA-seq tdTom+ cells at E15.5 (total 276 cells; n = 6 pooled hearts).

(B) Dot plot showing proportion of cells in each cluster expressing selected genes. Dot size represents percentage of cells expressing, and color scale indicates average expression level.

(C) Pseudotime trajectory of tdTom+ cells at E15.5, showing bifurcation to branch A (mural) or branch B (mesenchymal 1). Cells colored by cluster identity.

(D) Pseudotime trajectory colored by the expression level of *Rgs5*, representing mural fate, and *Dpt*, indicating mature mesenchymal state.

(E) Flow cytometric analysis of E15.5 hearts showing percentage of tdTom+ cells expressing either *Rgs5* or *Dpt*.

(F) ISH for *Rgs5*, *Pecam1*, *tdTomato*, and *Dpt* on E15.5 reveals tdTom+ cells expressing *Rgs5*+ neighboring vessels labeled with *Pecam1*. *TdTom*+ *Dpt*+ cells are present near the epicardium, and deeper within the heart.

(G) Pseudotime trajectory colored by expression level of selected epicardial genes.

Scale bars, 20 μm (F).

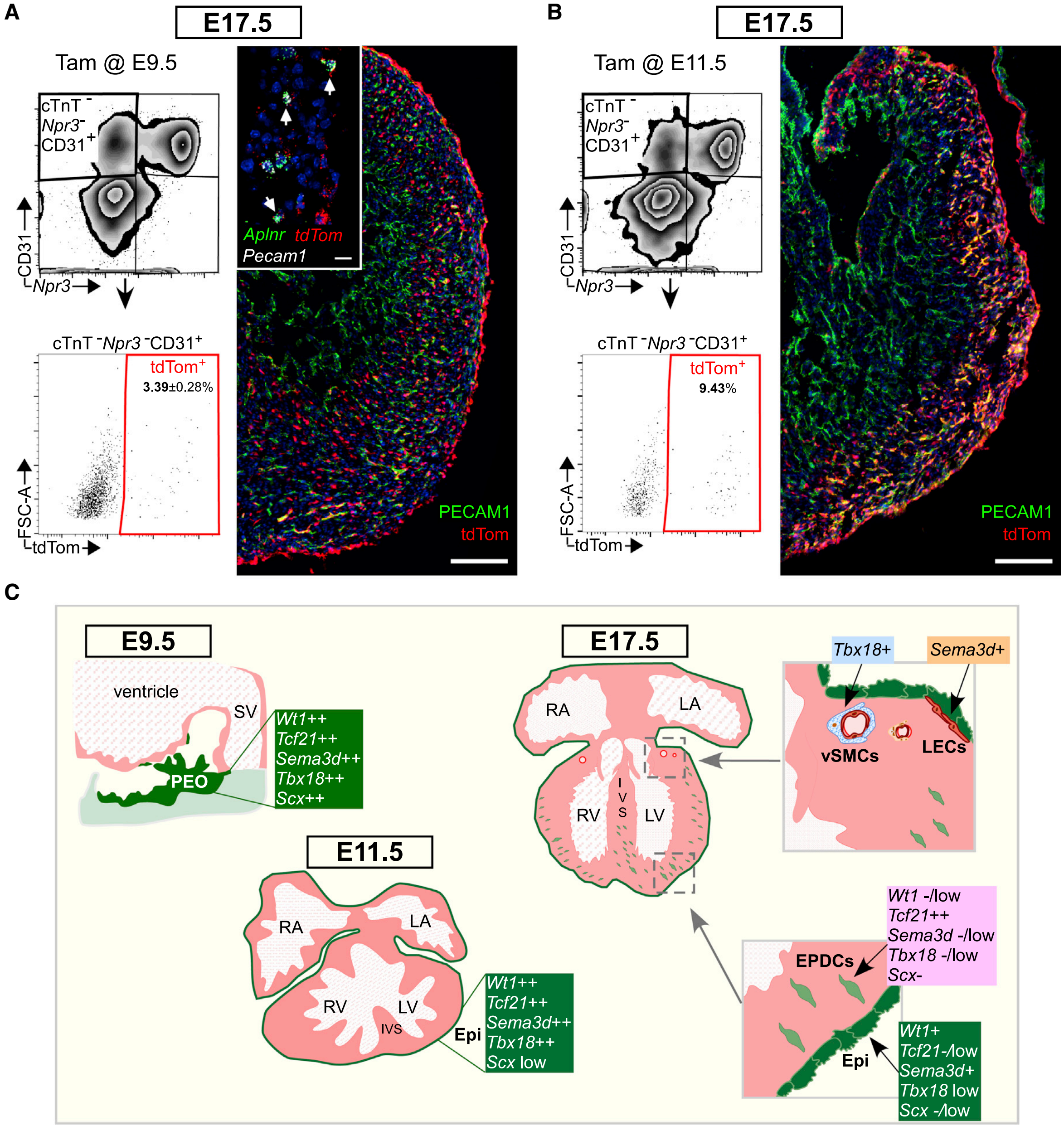

Figure 7. Wt1CreERT2 Targets Coronary Endothelial Cells

(A) Flow cytometric analysis of E17.5 hearts induced with tamoxifen (80 mg/kg) at E9.5. Endothelial cells were selected by gating cTnT-Npr3-CD31+ and downstream tdTom+ gating to determine epicardial contribution. Percentage tdTom+ endothelial cells expressed as mean ± SEM (n = 6 hearts). Immunostaining for PECAM1 on E17.5 heart sections reveals tdTom+ PECAM1+ endothelial cells. (inset) ISH for *Aplnr*, *tdTomato*, and *Pecam1* reveals that *tdTom+ Pecam1+* cells are *Aplnr+*, indicating sinus venosus origin.

(B) Flow cytometric analysis of E17.5 hearts induced with tamoxifen (80 mg/kg) at E11.5. Endothelial cells were selected by gating cTnT-Npr3-CD31+ and downstream tdTom+ gating to determine epicardial contribution (n = 2 hearts). Immunostaining for PECAM1 on E17.5 heart sections reveals tdTom+ PECAM1+ endothelial cells.

(C) Schematic summarizing marker profile of epicardium and its derivatives across the time course of development.

Scale bars, 200 μm (A and B) and 20 μm (A inset).

after EMT, potentially in response to extrinsic cues. Understanding the mechanisms of embryonic fate determination will inform therapeutic strategies to exploit the regenerative potential of epicardial cells.

EXPERIMENTAL PROCEDURES

Mouse Strains

Males homozygous for *Rosa26tdTom* (Madisen et al., 2010) and heterozygous for *Wt1CreERT2* (Zhou et al., 2008) were crossed with C57BL/6 females. Pregnant females were oral gavaged with 80 mg/kg tamoxifen at E9.5 or E11.5 (where stated otherwise). All procedures were approved by the University of Oxford Animal Welfare and Ethical Review Board, in accordance with Animals (Scientific Procedures) Act 1986 (Home Office, UK).

RNAscope

RNAscope Multiplex Fluorescent v.2 assay (ACD) was performed on cryosections according to the manufacturer's instructions, with minor modifications stated in the supplemental material . Probes, including negative control, are detailed in the supplemental material. Probes were optimized for hybridization at 40°C, which permits multiplexing without compromising signal. TSA plus fluorophores was used: fluorescein (1:500), Cy3 (1:1,000), Cy5 (1:1,500).

PrimeFlow RNA Assay

Enzymatically dissociated hearts were processed using a PrimeFlow RNA Assay Kit (Thermo Fisher Scientific), with staining for cell viability, CD31, WT1, and cardiac troponin T. Probes, detailed in the supplemental material , were used against epicardial markers.

scRNA-Seq and Analysis

E7.75_E8.25_E9.25 10× Chromium data (GSE126128) was downloaded from UCSC Cell Browser as raw UMI count matrix (de Soysa et al., 2019). Only E9.25 were selected for further analysis. E10.5 heart STRT-seq data were downloaded as TPM from GEO (GSM3027035) (Dong et al., 2018) and E10.5 heart SMART-seq2 data were downloaded as raw counts from GSE76118 (Li et al., 2016). All scRNA-seq datasets were analyzed using Seurat (Butler et al., 2018; Stuart et al., 2018) in R as follows: principal component analysis was used to cluster cells, which were visualized with the tSNE and UMAP method (Becht et al., 2019; Butler et al., 2018). Up to three rounds of clustering iterations were required for larger datasets—E9.25 and E10.5 dataset 1—to separate epicardial cells from: cell types showing similar transcriptomic profile (CM progenitors and STM) and doublets (Epi-CM; Epi-Mes), respectively. For E15.5 scRNA-seq, tamoxifen was administered at E9.5 and E11.5 (40 mg/kg at each stage), ventricles were dissociated and tdTom+ cells sorted for SMART-seq2 on Illumina NextSeq 500 platform. Reads were processed as described in the Supplemental Information and clustering performed as above. Accession Numbers: The accession number for the E15.5 sequencing data reported in this paper (FASTQ files and scaled count matrix available) is GEO: GSE145832. Normalized Seurat data were imported into Monocle2 for pseudotime analysis (Qiu et al., 2017).

Detailed protocols provided in Supplemental Experimental Procedures.

ACCESSION NUMBERS

E15.5 epicardial lineage scRNA-seq data have been deposited in the GEO data repository under accession number GSE145832.

SUPPLEMENTAL INFORMATION

Supplemental Information can be found online at https://doi.org/10.1016/j.stemcr.2020.04.002.

AUTHOR CONTRIBUTIONS

Conceptualization, I.-E.L. and N.S.; Experimental Design, I.-E.L., A.N.R., and N.S.; Data Acquisition and Analysis, I.-E.L. and A.N.R.; Writing – Original Draft, I.-E.L.; Writing – Review & Editing, A.N.R. and N.S.; Writing – Figure Preparation, A.N.R.; Bioinformatic Analysis, I.E.-L. and A.N.R.; Supervision, A.N.R. and N.S.; Funding Acquisition, N.S.

ACKNOWLEDGMENTS

We thank Prof William Pu, Harvard, for the *Wt1*CreERT2 line; Dr Madeleine Lemieux (Bioinfo) for processing SMART-seq2 data; Dr Neil Ashley, WIMM Single cell facility, for sequencing, the Dunn School Flow Cytometry facility for advice, Micron for microscopy facilities, and Biomedical Services staff for animal husbandry. This work was funded by the British Heart Foundation (BHF): DPhil Studentship (FS/15/68/32042); Project grant (PG/16/27/32114); and BHF Ian Fleming Senior Basic Science Research Fellowship (FS/13/4/30045); Oxbridge Centre of Regenerative Medicine (RM/13/3/30159).

Received: June 28, 2019
Revised: April 2, 2020
Accepted: April 2, 2020
Published: April 30, 2020

REFERENCES

Acharya, A., Baek, S.T., Banfi, S., Eskiocak, B., and Tallquist, M.D. (2011). Efficient inducible Cre-mediated recombination in Tcf21cell lineages in the heart and kidney. Genesis *49*, 870–877.

Acharya, A., Baek, S.T., Huang, G., Eskiocak, B., Goetsch, S., Sung, C.Y., Banfi, S., Sauer, M.F., Olsen, G.S., Duffield, J.S., et al. (2012). The bHLH transcription factor Tcf21 is required for lineage-specific EMT of cardiac fibroblast progenitors. Development *139*, 2139–2149.

Baran-Gale, J., Chandra, T., and Kirschner, K. (2017). Experimental design for single-cell RNA sequencing. Brief. Funct. Genomics *17*, 233–239.

Barnes, R.M., Firulli, B.A., VanDusen, N.J., Morikawa, Y., Conway, S.J., Cserjesi, P., Vincentz, J.W., and Firulli, A.B. (2011). Hand2 loss-of-function in Hand1-expressing cells reveals distinct roles in epicardial and coronary vessel development. Circ. Res. *108*, 940–949.

Barnette, D.N., VandeKopple, M., Wu, Y., Willoughby, D.A., and Lincoln, J. (2014). RNA-seq analysis to identify novel roles of scleraxis during embryonic mouse heart valve remodeling. PLoS One *9*, e101425.

Becht, E., McInnes, L., Healy, J., Dutertre, C.-A., Kwok, I.W.H., Ng, L.G., Ginhoux, F., and Newell, E.W. (2019). Dimensionality reduction for visualizing single-cell data using UMAP. Nat. Biotechnol. *37*, 38–44.

Braitsch, C.M., Combs, M.D., Quaggin, S.E., and Yutzey, K.E. (2012). Pod1/Tcf21 is regulated by retinoic acid signaling and inhibits differentiation of epicardium-derived cells into smooth muscle in the developing heart. Dev. Biol. *368*, 345–357.

Butler, A., Hoffman, P., Smibert, P., Papalexi, E., and Satija, R. (2018). Integrating single-cell transcriptomic data across different conditions, technologies, and species. Nat. Biotechnol. *36*, 411.

Cai, C.L., Martin, J.C., Sun, Y., Cui, L., Wang, L., Ouyang, K., Yang, L., Bu, L., Liang, X., Zhang, X., et al. (2008). A myocardial lineage derives from Tbx18 epicardial cells. Nature *454*, 104–108.

Cañete, A., Carmona, R., Ariza, L., Sánchez, M.J., Rojas, A., and Muñoz-Chápuli, R. (2017). A population of hematopoietic stem cells derives from GATA4-expressing progenitors located in the placenta and lateral mesoderm of mice. Haematologica *102*, 647.

Cano, E., Carmona, R., Ruiz-Villalba, A., Rojas, A., Chau, Y.-Y., Wagner, K.D., Wagner, N., Hastie, N.D., Muñoz-Chápuli, R., and Pérez-Pomares, J.M. (2016). Extracardiac septum transversum/proepicardial endothelial cells pattern embryonic coronary arterio–venous connections. Proc. Natl. Acad. Sci. U S A *113*, 656–661.

Cao, J., Navis, A., Cox, B.D., Dickson, A.L., Gemberling, M., Karra, R., Bagnat, M., and Poss, K.D. (2016). Single epicardial cell transcriptome sequencing identifies Caveolin 1 as an essential factor in zebrafish heart regeneration. Development *143*, 232–243.

Carmona, R., Canete, A., Cano, E., Ariza, L., Rojas, A., and Munoz-Chapuli, R. (2016). Conditional deletion of WT1 in the septum transversum mesenchyme causes congenital diaphragmatic hernia in mice. eLife *5*. https://doi.org/10.7554/eLife.16009.

Carmona, R., Barrena, S., López Gambero, A.J., Rojas, A., and Muñoz-Chápuli, R. (2020). Epicardial cell lineages and the origin of the coronary endothelium. FASEB J. *34*, 5223–5239.

Chen, H.I., Sharma, B., Akerberg, B.N., Numi, H.J., Kivelä, R., Saharinen, P., Aghajanian, H., McKay, A.S., Bogard, P.E., Chang, A.H., et al. (2014). The sinus venosus contributes to coronary vasculature through VEGFC-stimulated angiogenesis. Development *141*, 4500–4512.

Chen, Q., Zhang, H., Liu, Y., Adams, S., Eilken, H., Stehling, M., Corada, M., Dejana, E., Zhou, B., and Adams, R.H. (2016). Endothelial cells are progenitors of cardiac pericytes and vascular smooth muscle cells. Nat. Commun. *7*, 12422.

Christoffels, V.M., Mommersteeg, M.T., Trowe, M.O., Prall, O.W., de Gier-de Vries, C., Soufan, A.T., Bussen, M., Schuster-Gossler, K., Harvey, R.P., Moorman, A.F., et al. (2006). Formation of the venous pole of the heart from an Nkx2-5-negative precursor population requires Tbx18. Circ. Res. *98*, 1555–1563.

Christoffels, V.M., Grieskamp, T., Norden, J., Mommersteeg, M.T., Rudat, C., and Kispert, A. (2009). Tbx18 and the fate of epicardial progenitors. Nature *458*, E8–E9, discussion E9-10.

Corrigan, A.M., Tunnacliffe, E., Cannon, D., and Chubb, J.R. (2016). A continuum model of transcriptional bursting. eLife *5*. https://doi.org/10.7554/eLife.13051.

Dong, J., Hu, Y., Fan, X., Wu, X., Mao, Y., Hu, B., Guo, H., Wen, L., and Tang, F. (2018). Single-cell RNA-seq analysis unveils a prevalent epithelial/mesenchymal hybrid state during mouse organogenesis. Genome Biol. *19*, 31.

Dubé, K.N., Thomas, T.M., Munshaw, S., Rohling, M., Riley, P.R., and Smart, N. (2017). Recapitulation of developmental mechanisms to revascularize the ischemic heart. JCI Insight *2*, e96800.

Duim, S.N., Kurakula, K., Goumans, M.J., and Kruithof, B.P. (2015). Cardiac endothelial cells express Wilms' tumor-1: Wt1 expression in the developing, adult and infarcted heart. J. Mol. Cell. Cardiol. *81*, 127–135.

Gambardella, L., McManus, S.A., Moignard, V., Sebukhan, D., Delaune, A., Andrews, S., Bernard, W.G., Morrison, M.A., Riley, P.R., Göttgens, B., et al. (2019). BNC1 regulates cell heterogeneity in human pluripotent stem cell-derived epicardium. Development *146*, dev174441.

Greulich, F., and Kispert, A. (2013). Epicardial lineages. J. Dev. Biol. *1*, 32.

Guimaraes-Camboa, N., Cattaneo, P., Sun, Y., Moore-Morris, T., Gu, Y., Dalton, N.D., Rockenstein, E., Masliah, E., Peterson, K.L., Stallcup, W.B., et al. (2017). Pericytes of multiple organs do not behave as mesenchymal stem cells in vivo. Cell Stem Cell *20*, 345–359.e5.

Hayashi, S., and McMahon, A.P. (2002). Efficient recombination in diverse tissues by a tamoxifen-inducible form of Cre: a tool for temporally regulated gene activation/inactivation in the mouse. Dev. Biol. *244*, 305–318.

Jurisic, G., Maby-El Hajjami, H., Karaman, S., Ochsenbein, A.M., Alitalo, A., Siddiqui, S.S., Ochoa Pereira, C., Petrova, T.V., and Detmar, M. (2012). An unexpected role of semaphorin3a-neuropilin-1 signaling in lymphatic vessel maturation and valve formation. Circ. Res. *111*, 426–436.

Kalinichenko, V.V., Zhou, Y., Bhattacharyya, D., Kim, W., Shin, B., Bambal, K., and Costa, R.H. (2002). Haploinsufficiency of the mouse Forkhead Box f1 gene causes defects in gall bladder development. J. Biol. Chem. *277*, 12369–12374.

Katz, T.C., Singh, M.K., Degenhardt, K., Rivera-Feliciano, J., Johnson, R.L., Epstein, J.A., and Tabin, C.J. (2012). Distinct compartments of the proepicardial organ give rise to coronary vascular endothelial cells. Dev. Cell *22*, 639–650.

Kolterud, A., Wandzioch, E., and Carlsson, L. (2004). Lhx2 is expressed in the septum transversum mesenchyme that becomes an integral part of the liver and the formation of these cells is independent of functional Lhx2. Gene Expr. Patterns *4*, 521–528.

Krainock, M., Toubat, O., Danopoulos, S., Beckham, A., Warburton, D., and Kim, R. (2016). Epicardial epithelial-to-mesenchymal transition in heart development and disease. J. Clin. Med. *5*, 27.

Kruithof, B.P., van Wijk, B., Somi, S., Kruithof-de Julio, M., Perez Pomares, J.M., Weesie, F., Wessels, A., Moorman, A.F., and van den Hoff, M.J. (2006). BMP and FGF regulate the differentiation of multipotential pericardial mesoderm into the myocardial or epicardial lineage. Dev. Biol. *295*, 507–522.

Li, G., Xu, A., Sim, S., Priest, J.R., Tian, X., Khan, T., Quertermous, T., Zhou, B., Tsao, P.S., Quake, S.R., et al. (2016). Transcriptomic profiling maps anatomically patterned subpopulations among single embryonic cardiac cells. Dev. Cell *39*, 491–507.

Lie-Venema, H., van den Akker, N.M., Bax, N.A., Winter, E.M., Maas, S., Kekarainen, T., Hoeben, R.C., deRuiter, M.C., Poelmann, R.E., and Gittenberger-de Groot, A.C. (2007). Origin, fate, and function of epicardium-derived cells (EPDCs) in normal and abnormal cardiac development. ScientificWorldJournal *7*, 1777–1798.

Liu, Q., Zhang, H., Tian, X., He, L., Huang, X., Tan, Z., Yan, Y., Evans, S.M., Wythe, J.D., and Zhou, B. (2016). Smooth muscle origin of postnatal 2nd CVP is pre-determined in early embryo. Biochem. Biophys. Res. Commun. *471*, 430–436.

Lu, J., Chang, P., Richardson, J.A., Gan, L., Weiler, H., and Olson, E.N. (2000). The basic helix-loop-helix transcription factor capsulin controls spleen organogenesis. Proc. Natl. Acad. Sci. U S A *97*, 9525–9530.

Madisen, L., Zwingman, T.A., Sunkin, S.M., Oh, S.W., Zariwala, H.A., Gu, H., Ng, L.L., Palmiter, R.D., Hawrylycz, M.J., Jones, A.R., et al. (2010). A robust and high-throughput Cre reporting and characterization system for the whole mouse brain. Nat. Neurosci. *13*, 133–140.

Maya-Ramos, L., Cleland, J., Bressan, M., and Mikawa, T. (2013). Induction of the proepicardium. J. Dev. Biol. *1*, 82–91.

Merki, E., Zamora, M., Raya, A., Kawakami, Y., Wang, J., Zhang, X., Burch, J., Kubalak, S.W., Kaliman, P., Belmonte, J.C.I., et al. (2005). Epicardial retinoid X receptor α is required for myocardial growth and coronary artery formation. Proc. Natl. Acad. Sci. U S A *102*, 18455–18460.

Padovan-Merhar, O., Nair, G.P., Biaesch, A.G., Mayer, A., Scarfone, S., Foley, S.W., Wu, A.R., Churchman, L.S., Singh, A., and Raj, A. (2015). Single mammalian cells compensate for differences in cellular volume and DNA copy number through independent global transcriptional mechanisms. Mol. Cell *58*, 339–352.

Perez-Pomares, J.M., Carmona, R., Gonzalez-Iriarte, M., Macias, D., Guadix, J.A., and Munoz-Chapuli, R. (2004). Contribution of mesothelium-derived cells to liver sinusoids in avian embryos. Dev. Dyn. *229*, 465–474.

Picelli, S., Faridani, O.R., Björklund, Å.K., Winberg, G., Sagasser, S., and Sandberg, R. (2014). Full-length RNA-seq from single cells using Smart-seq2. Nat. Protoc. *9*, 171.

Pu, W., He, L., Han, X., Tian, X., Li, Y., Zhang, H., Liu, Q., Huang, X., Zhang, L., Wang, Q.D., et al. (2018). Genetic targeting of organ-specific blood vessels. Circ. Res. *123*, 86–99.

Qiu, X., Mao, Q., Tang, Y., Wang, L., Chawla, R., Pliner, H.A., and Trapnell, C. (2017). Reversed graph embedding resolves complex single-cell trajectories. Nat. Methods *14*, 979–982.

Red-Horse, K., Ueno, H., Weissman, I.L., and Krasnow, M.A. (2010). Coronary arteries form by developmental reprogramming of venous cells. Nature *464*, 549.

Ren, X., Ustiyan, V., Pradhan, A., Cai, Y., Havrilak, J.A., Bolte, C.S., Shannon, J.M., Kalin, T.V., and Kalinichenko, V.V. (2014). FOXF1 transcription factor is required for formation of embryonic vasculature by regulating VEGF signaling in endothelial cells. Circ. Res. *115*, 709–720.

Rudat, C., Grieskamp, T., Röhr, C., Airik, R., Wrede, C., Hegermann, J., Herrmann, B.G., Schuster-Gossler, K., and Kispert, A. (2014). Upk3b is dispensable for development and integrity of urothelium and mesothelium. PLoS One *9*, e112112.

Ruiz-Villalba, A., Ziogas, A., Ehrbar, M., and Pérez-Pomares, J.M. (2013). Characterization of epicardial-derived cardiac interstitial cells: differentiation and mobilization of heart fibroblast progenitors. PLoS One *8*, e53694.

de Soysa, T.Y., Ranade, S.S., Okawa, S., Ravichandran, S., Huang, Y., Salunga, H.T., Schricker, A., del Sol, A., Gifford, C.A., and Srivastava, D. (2019). Single-cell analysis of cardiogenesis reveals basis for organ-level developmental defects. Nature *572*, 120–124.

Stuart, T., Butler, A., Hoffman, P., Hafemeister, C., Papalexi, E., Mauck, W.M., Stoeckius, M., Smibert, P., and Satija, R. (2018). Comprehensive integration of single cell data. Cell *177*, 1888–1902.e21.

Su, T., Stanley, G., Sinha, R., D'Amato, G., Das, S., Rhee, S., Chang, A.H., Poduri, A., Raftrey, B., Dinh, T.T., et al. (2018). Single-cell analysis of early progenitor cells that build coronary arteries. Nature *559*, 356–362.

Tandon, P., Wilczewski, C.M., Williams, C.E., and Conlon, F.L. (2016). The Lhx9-integrin pathway is essential for positioning of the proepicardial organ. Development *143*, 831–840.

Tandon, P., Miteva, Y.V., Kuchenbrod, L.M., Cristea, I.M., and Conlon, F.L. (2013). Tcf21 regulates the specification and maturation of proepicardial cells. Development *140*, 2409–2421.

Tian, X., Hu, T., Zhang, H., He, L., Huang, X., Liu, Q., Yu, W., He, L., Yang, Z., Zhang, Z., et al. (2013). Subepicardial endothelial cells invade the embryonic ventricle wall to form coronary arteries. Cell Res. *23*, 1075.

Tian, X., Hu, T., Zhang, H., He, L., Huang, X., Liu, Q., Yu, W., He, L., Yang, Z., Yan, Y., et al. (2014). De novo formation of a distinct coronary vascular population in neonatal heart. Science *345*, 90–94.

Volz, K.S., Jacobs, A.H., Chen, H.I., Poduri, A., McKay, A.S., Riordan, D.P., Kofler, N., Kitajewski, J., Weissman, I., and Red-Horse, K. (2015). Pericytes are progenitors for coronary artery smooth muscle. eLife *4*, e10036.

Wei, K., Díaz-Trelles, R., Liu, Q., Diez-Cuñado, M., Scimia, M.-C., Cai, W., Sawada, J., Komatsu, M., Boyle, J.J., Zhou, B., et al. (2015). Developmental origin of age-related coronary artery disease. Cardiovasc. Res. *107*, 287–294.

Weinberger, M., Simões, F.C., Patient, R., Sauka-Spengler, T., and Riley, P.R. (2020). Functional heterogeneity within the developing zebrafish epicardium. Dev. Cell *52*, 574–590.e6.

Weinreb, C., Wolock, S., Tusi, B.K., Socolovsky, M., and Klein, A.M. (2018). Fundamental limits on dynamic inference from single-cell snapshots. Proc. Natl. Acad. Sci. U S A *115*, E2467–E2476.

Xiao, Y., Hill, M.C., Zhang, M., Martin, T.J., Morikawa, Y., Wang, S., Moise, A.R., Wythe, J.D., and Martin, J.F. (2018). Hippo signaling plays an essential role in cell state transitions during cardiac fibroblast development. Dev. Cell *45*, 153–169.e6.

Zhang, H., Pu, W., Li, G., Huang, X., He, L., Tian, X., Liu, Q., Zhang, L., Wu, S.M., Sucov, H.M., et al. (2016). Endocardium minimally contributes to coronary endothelium in the embryonic ventricular free walls. Circ. Res. *118*, 1880–1893.

Zhou, B., and Pu, W.T. (2012). Genetic Cre-loxP assessment of epicardial cell fate using Wt1-driven Cre alleles. Circ. Res. *111*, e276–e280.

Zhou, B., Ma, Q., Rajagopal, S., Wu, S.M., Domian, I., Rivera-Feliciano, J., Jiang, D., von Gise, A., Ikeda, S., Chien, K.R., et al. (2008). Epicardial progenitors contribute to the cardiomyocyte lineage in the developing heart. Nature *454*, 109–113.

Stem Cell Reports, Volume *14*

Supplemental Information

Spatiotemporal Analysis Reveals Overlap of Key Proepicardial Markers in the Developing Murine Heart

Irina-Elena Lupu, Andia N. Redpath, and Nicola Smart

Supplemental Information

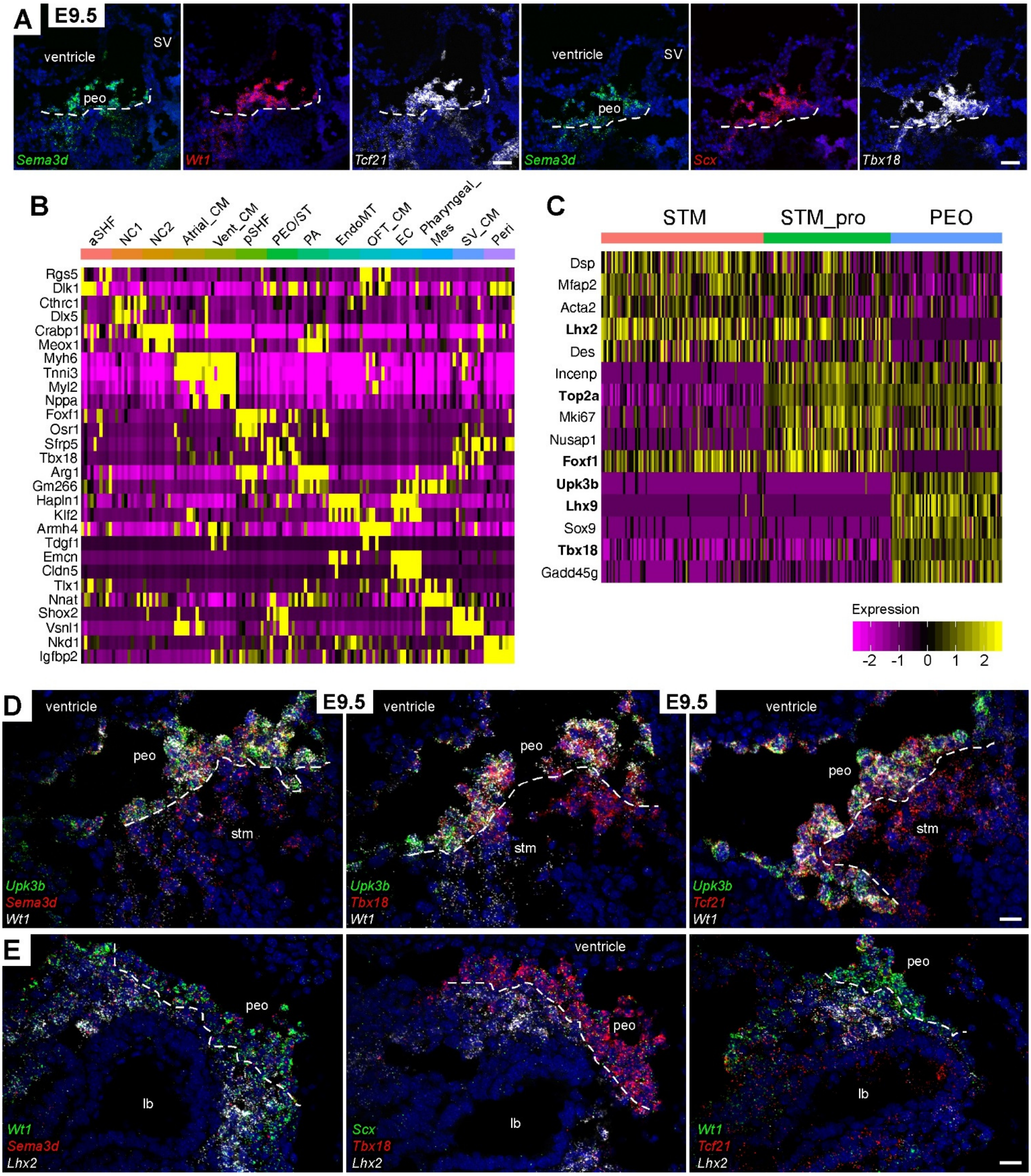

Figure S1. Gene expression of epicardial markers in the septum transversum region and confirmation of E9.25 scRNA-seq cluster identity. Related to Figure 1.

(A) RNA ISH of E9.5 embryo cryosections shows overlapping expression of *Sema3d*, *Wt1*, *Tcf21*, *Scx*, and *Tbx18* mRNA only in the proepicardial organ (peo; dashed line) (n=5 embryos). (B) Heatmap of top 2 differentially expressed genes for each cluster in the E9.25 scRNA-Seq data. High expression is indicated in yellow. (C) Heatmap of top 5 differentially expressed genes for each cluster in the PEO/STM subset. (D) RNA ISH of *Sema3d, Wt1, Tbx18, Tcf21* and *Upk3b* on E9.5 sagittal sections reveals expression of these genes in the proepicardium, demarcated by *Upk3b* (n=3 embryos). (E) RNA ISH of *Sema3d, Wt1, Tbx18, Tcf21, Scx* and *Lhx2*, shows expression of these genes in the septum transversum, demarcated by *Lhx2* (n=3 embryos). *EndoMT, endocardial-to-mesenchymal transition; Endo, endocardial cell; Mes, mesenchyme; PA-pharyngeal arch; aSHF, anterior second heart field; Peri, pericardium; pSHF, posterior second heart field; OFT_CM, outflow tract cardiomyocyte; Vent_CM, ventricular cardiomyocytes; SV_CM, sinus venosus cardiomyocytes; PEO/STM, proepicardium/septum transversum; lb, liver bud.* Scale bars: 50µm A; 20µm D-E.

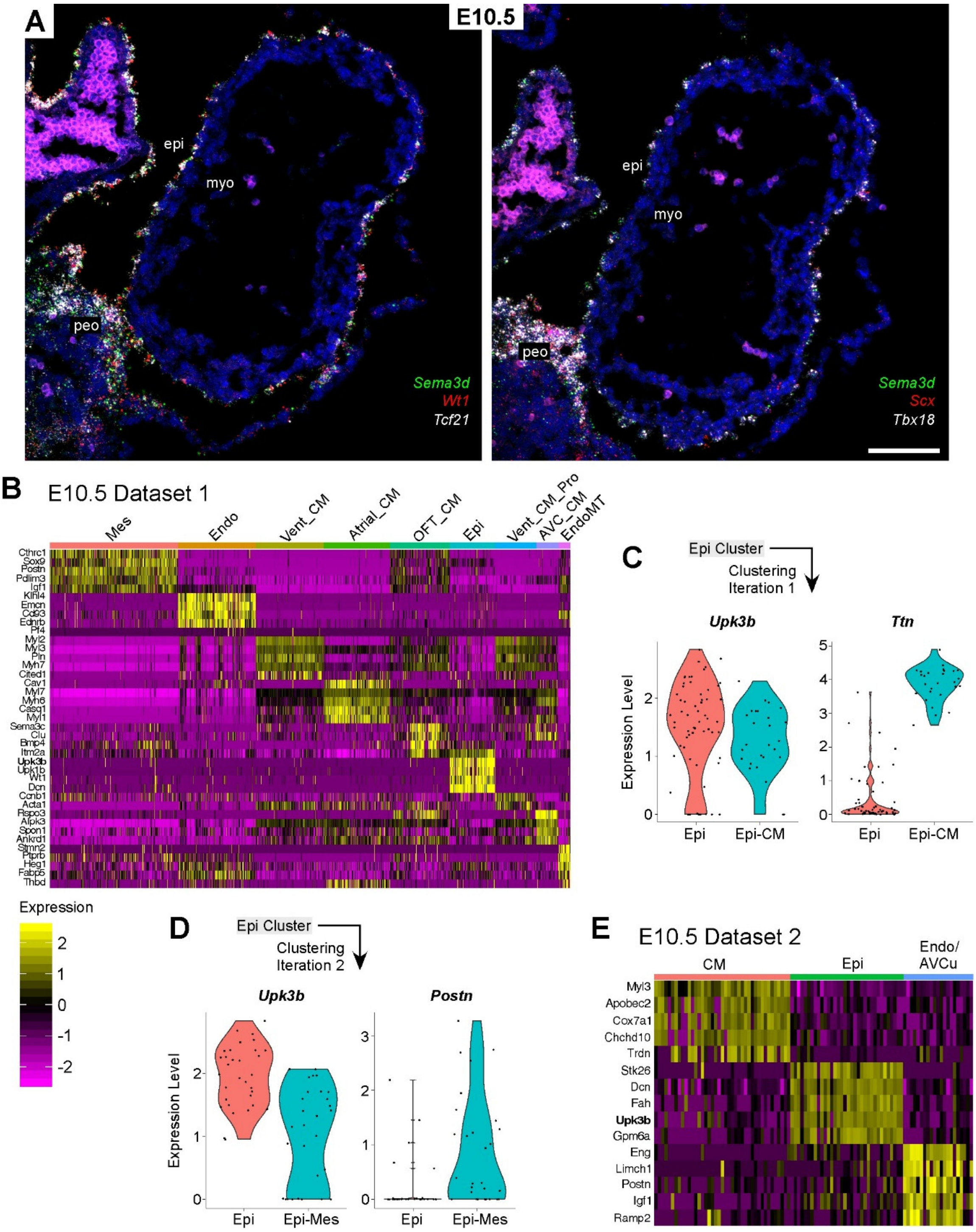

Figure S2. Expression of epicardial markers at E10.5 and confirmation of E10.5 heart scRNA-seq cluster identity. Related to Figure 2.
(A) RNA ISH of E10.5 embryo cryosections shows overlapping expression of *Sema3d*, *Wt1*, *Tcf21*, *Scx*, and *Tbx18* mRNA in the proepicardial organ (peo) and in the epicardium (epi) (n=2 embryos). (B) Heatmap of the top 5 differentially expressed genes for each cluster in E10.5 scRNA-Seq dataset 1. High expression is indicated in yellow. (C) Violin plots showing epicardial-cardiomyocyte (Epi-CM) doublets expressing *Upk3b* and *Ttn* in the first clustering iteration of Epi cluster in B. (D) Violin plots showing epicardial-mesenchymal (Epi-Mes) doublets expressing *Upk3b* and *Postn* in the next clustering iteration of Epi cluster in C. (E) Heatmap of top 5 differentially expressed genes for each cluster in E10.5 scRNA-Seq dataset 2. *peo, proepicardial organ; Vent_CM, ventricular cardiomyocytes, Pro, proliferating; OFT_CM, outflow tract cardiomyocytes; Mes, mesenchyme; EndoMT, endocardial-to-mesenchymal transition; Endo, endocardium; AVC_CM, atrioventricular canal cardiomyocytes; Epi, epicardium, AVCu-atrioventricular cushion.* Scale bars: 100µm A.

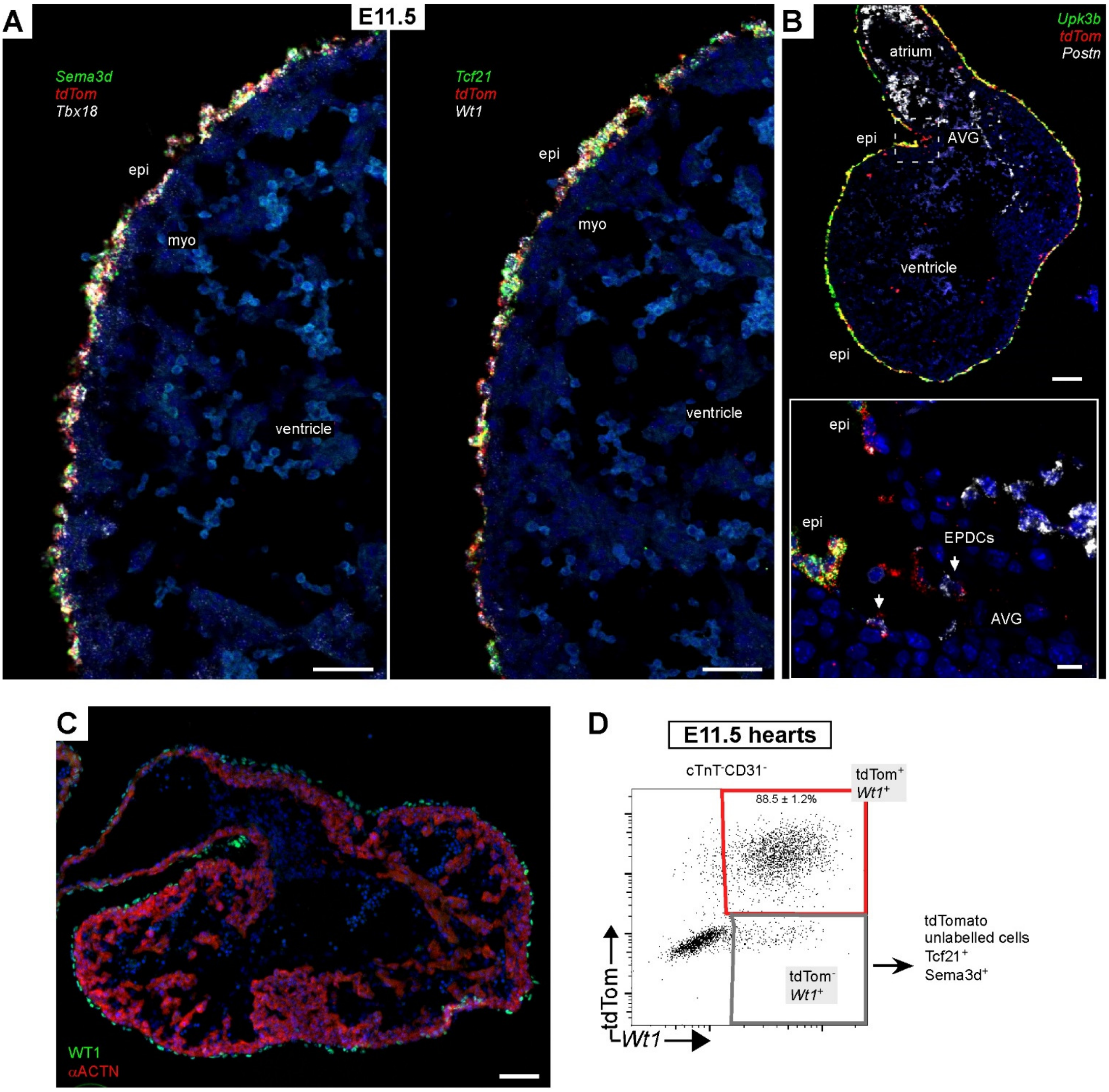

Figure S3. ***Wt1, Sema3d, Tcf21, Tbx18, Scx*** **and** ***Upk3b*** **are expressed throughout the epicardial layer at E11.5. Related to Figure 3.**

(A) RNA ISH on E11.5 heart sections for *Wt1, Sema3d, Tcf21, Tbx18* and *tdTom* reveals overlap of marker genes throughout the epicardial layer (n=4 hearts). (B) RNA ISH for *Upk3b, tdTom* and *Postn* reveals overlap of *Upk3b* and *tdTom* throughout the epicardial layer, with the exception of cells in the atrioventricular groove (AVG) that express *Postn*, indicating mesenchymal state (EPDCs, white arrow; inset) (n=2 hearts). (C) Immunostaining for WT1 and α-sarcomeric-actinin on E11.5 heart sections reveals WT1 expression in all epicardial cells (n=5 hearts). (D) Flow cytometric analysis of *Wt1* transcript and tdTom at E11.5 reveals efficient labelling of epicardial cells by tdTom (mean ± s.e.m; n=29 hearts; ≥3 independent experiments). Scale bars: 50μm A; 100μm B-C; 10μm inset B.

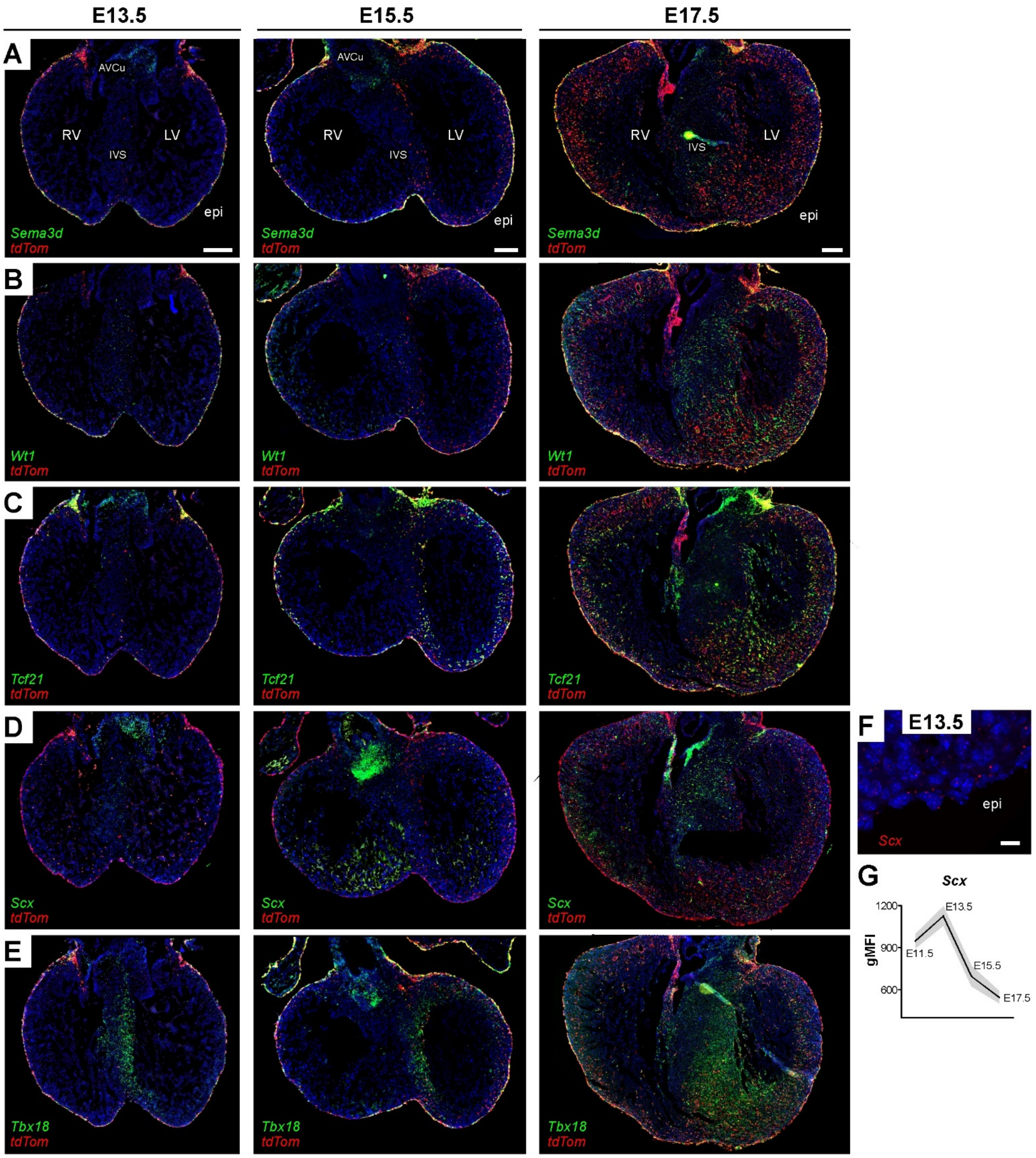

Figure S4. Non-epicardial expression of *Sema3d*, *Wt1*, *Tcf21*, *Scx*, and *Tbx18* at later stages of development. Related to Figure 4.
(A) RNA ISH co-staining of *Sema3d* (green) and *tdTom* (red) at E13.5, E15.5, E17.5 shows overlap in the epicardium. *Sema3d* is also expressed in the atrioventricular cushion (AVCu) and in lymphatic endothelium. (B) RNA ISH co-staining of *Wt1* (green) and *tdTom* (red) at E13.5, E15.5, E17.5 shows overlap in the epicardium. *Wt1* is also expressed in coronary endothelial cells before E13.5. (C) RNA ISH co-staining of *Tcf21* (green) and *tdTom* (red) at E13.5, E15.5, E17.5 shows overlap in the epicardium and in EPDCs. *Tcf21* is also expressed in AVCu and in interstitial fibroblasts. (D) RNA ISH co-staining of *Scx* (green) and *tdTom* (red) at E13.5, E15.5, and E17.5 shows very low expression of Scx in the epicardium. *Scx* is highly expressed in the atrioventricular cushion (AVCu). (E) RNA ISH co-staining of Tbx18 (green) and tdTomato (red) at E13.5, E15.5, E17.5 shows overlap in the epicardium. *Tbx18* is also expressed in cardiomyocytes in the septum and left ventricle and in vascular smooth muscle cells in the aorta (n=3 hearts). (F) RNA ISH of *Scx* at E13.5, showing very low detection of transcripts in the epicardium. (G) Flow cytometric analysis of *Scx* in the epicardial population, demonstrating downregulated expression over development (mean gMFI ± s.e.m; 4 independent experiments). gMFI, geometric Mean Fluorescence

Intensity. AVCu, atrioventricular cushion; RV, right ventricle; IVS, intraventricular septum; LV, left ventricle; epi, epicardium. Scale bars: 200µm A; 10µm F.

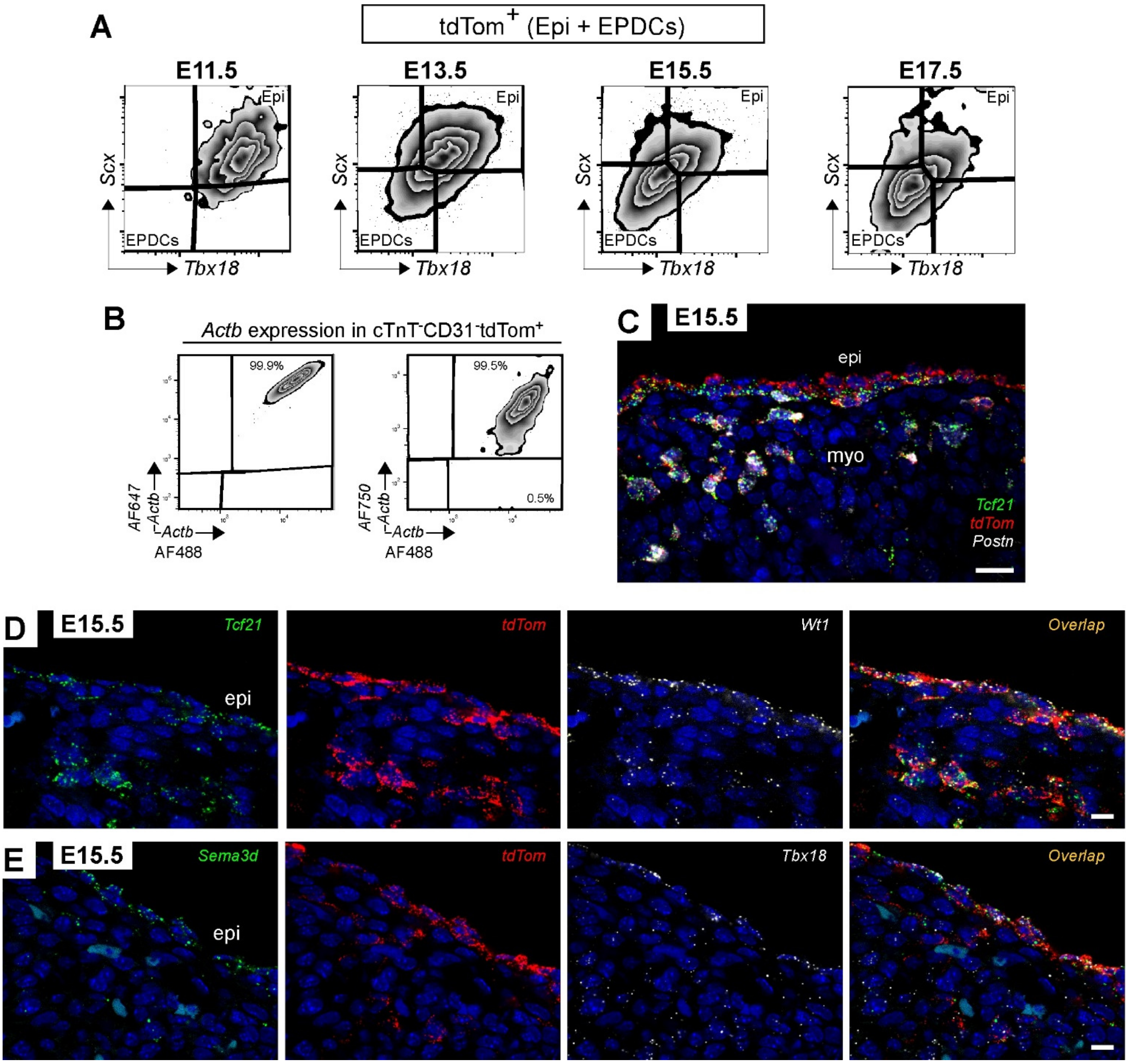

Figure S5. Epicardial marker expression decreases as epicardial cells transition to EPDCs. Related to Figure 5.
(A) Flow cytometric analysis of *Scx* and *Tbx18* expression in tdTom$^+$ cells, representing the epicardial lineage, shows downregulation of these markers in epicardial cells over development (n= as indicated in Figure 5D). (B) Multiplex analysis of *Actb* expression in tdTom+ cells reveals range of expression. (C) RNA ISH for *Tcf21, tdTom* and *Postn* on E15.5 sections reveals increased *Tcf21* expression in mesenchymal cells, demarcated by *Postn* (n=2 hearts). (D) RNA ISH for *Tcf21, tdTom, Wt1,* (E) *Sema3d* and *Tbx18* shows marker expression in the epicardium-derived cells (EPDCs) and epicardium at E15.5. Expression is downregulated in epicardium and EPDCs, with the exception of *Tcf21*, which is downregulated in epicardium but upregulated in EPDCs (n= 3 hearts). Scale bars: 20µm C; 10µm D-E.

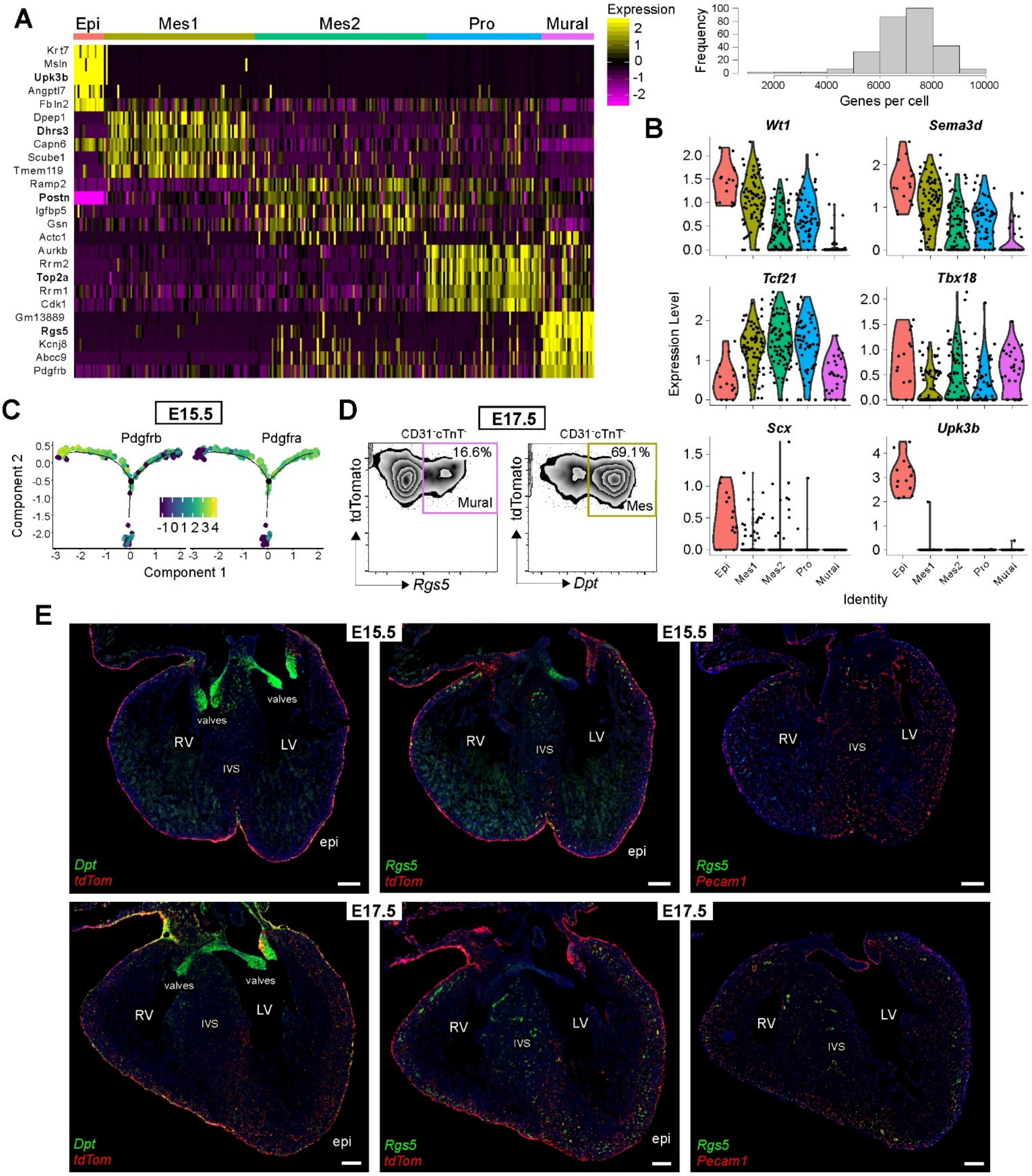

Figure S6. E15.5 scRNA-seq cluster identity, pseudotime representation of cell fate-associated genes and validation of EPDC differentiation. Related to Figure 6.

(A) Heatmap of top 5 differentially expressed genes per cluster in the E15.5 scRNA-Seq. High expression is indicated in yellow. The number of genes per cell captured is shown in the histogram. (B) Violin plots demonstrating expression levels in individuals cells for *Wt1, Sema3d, Tcf21, Tbx18, Scx, and Upk3b,* per cluster in the E15.5 scRNA-Seq. (C) Visualisation of Pdgfr genes in pseudotime reveals two potential cell fates, one associated with mural fate (*Pdgfrβ*) and one associated with mesenchymal/fibroblast fate (*Pdgfrα*). (D) Flow cytometry analysis of E17.5 dissociated hearts reveals percentage of tdTom+ cells, representing EPDCs, positive for *Rgs5* or *Dpt* (mean ± s.e.m; n=6 hearts). (E) RNA ISH for *Dpt, tdTom, Rgs5* and *Pecam1* on E15.5 and E17.5 heart sections reveals appearance of differentiated cells at E15.5 and widespread expression at E17.5 (n=2-3 hearts). Scale bars: 200μm.

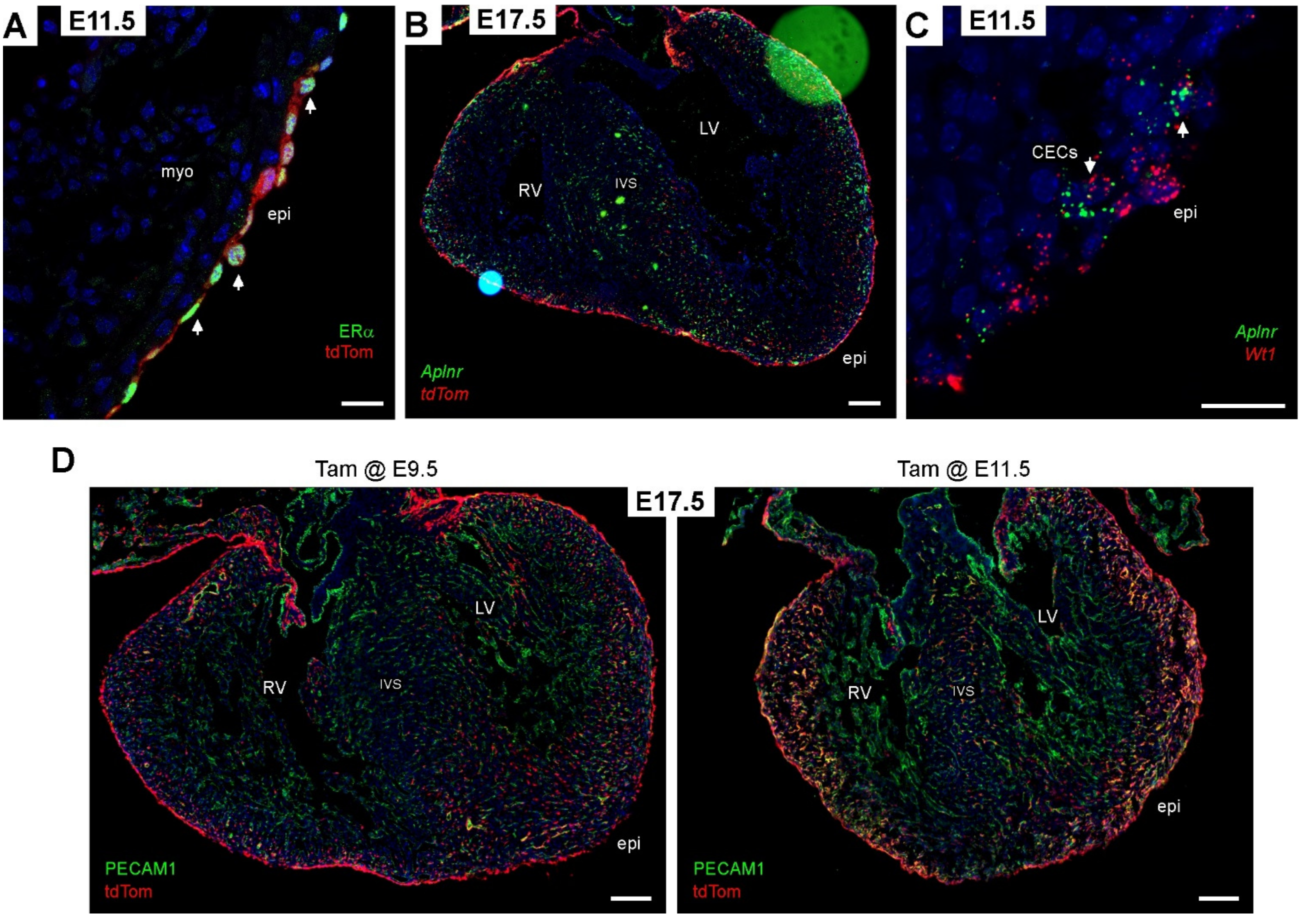

Figure S7. Wt1CreERT2 labels sinus-venosus derived endothelial cells. Related to Figure 7.
(A) Immunostaining for estrogen receptor α (ERα) reveals nuclear localisation at E11.5, indicating that Cre recombinase is active 48h post-tamoxifen administration at E9.5 (white arrows; n=2 hearts). (B) RNA ISH of *Aplnr* and *tdTom* at E17.5, showing expansion of tdTom-labelled SV-derived endothelial cells (n=2 hearts). (C) RNA ISH of *Aplnr* and *Wt1* at E11.5, showing expression of *Wt1* in SV-derived endothelial cells, demarcated by *Aplnr* (n= 1 heart). (D) Immunostaining for PECAM1 on E17.5 heart sections reveals overlap with tdTom+ cells after E9.5 and E11.5 tamoxifen administration (n= 2 hearts). Scale bars: 20µm A,C; 200µm B,D.

Table. S1: % Positive expression (SED) in tdTom+ epicardial population at E11.5

	Sema3d	*Wt1*	*Tcf21*	*Tbx18*	*Scx*
Number of values	3	3	3	3	3
Minimum	97.30	96.00	94.20	94.90	81.60
25% Percentile	97.30	96.00	94.20	94.90	81.60
Median	97.70	98.20	94.90	98.60	90.10
75% Percentile	98.90	98.20	97.40	99.50	94.40
Maximum	98.90	98.20	97.40	99.50	94.40
**Mean**	**97.97**	**97.47**	**95.50**	**97.67**	**88.70**
Std. Deviation	0.8327	1.270	1.682	2.438	6.514
Std. Error	*0.4807*	*0.7333*	*0.9713*	*1.408*	*3.761*

Table. S2: % Positive expression (SED) *Sema3d* in *Wt1*$^{+}$*Tcf21*$^{+}$ epicardial population

	E11.5	E13.5	E15.5	E17.5
Number of values	3	3	2	3
Minimum	98.20	99.50	99.20	96.20
25% Percentile	98.20	99.50	99.20	96.20
Median	98.90	99.70	99.30	96.70
75% Percentile	99.40	99.80	99.40	99.50
Maximum	99.40	99.80	99.40	99.50
**Mean**	**98.83**	**99.67**	**99.30**	**97.47**
Std. Deviation	0.6028	0.1528	0.1414	1.779
Std. Error	*0.3480*	*0.08819*	*0.1000*	*1.027*

Table. S3: % Positive expression (SED) *Scx* in *Tbx18*$^{+}$*Sema3d*$^{+}$ epicardial population

	E11.5	E13.5	E15.5	E17.5
Number of values	3	3	2	3
Minimum	82.80	88.70	97.10	87.90
25% Percentile	82.80	88.70	97.10	87.90
Median	93.60	98.80	97.30	93.20
75% Percentile	94.00	99.00	97.50	96.30
Maximum	94.00	99.00	97.50	96.30
**Mean**	**90.13**	**95.50**	**97.30**	**92.47**
Std. Deviation	6.354	5.890	0.2828	4.248
Std. Error	*3.668*	*3.400*	*0.2000*	*2.452*

**SED, Super-Enhanced Dmax Subtraction algorithm calculates percent positives when comparing histograms

Supplemental Experimental Procedures

Mouse strains. Males homozygous for the *Gt(ROSA)26Sor*$^{tm14(CAG\text{-}tdTomato)Hze}$ allele (Madisen et al., 2010) and heterozygous for *Wt1*$^{tm2(cre/ERT2)Wtp}$ (Zhou et al., 2008) were crossed with C57BL/6 females to generate embryos. The mice used were on a C57BL/6 genetic background. Pregnant females were oral gavaged with 80mg/kg tamoxifen (Sigma, #T5648) 9 days or 11 days (as specified) after the mating plug was detected. Tamoxifen was dissolved in peanut oil with 10% ethanol at a final concentration of 10mg/ml. The animals were kept in a controlled environment and all procedures used were approved by the University of Oxford Animal Welfare and Ethical Review Board in accordance with the Animals (Scientific Procedures) Act 1986 (Home Office, United Kingdom).

RNAscope & Immunofluorescence. RNAscope is a commercially available in-situ hybridization assay provided by Advanced Cell Diagnostics (ACD) (Wang et al., 2012). RNAscope was performed on 8-12 μm thick embryonic cryosections that had been fixed for 2h in 4%PFA and embedded in OCT (Tissue-Tek). The following catalogue probes were used: Wt1 #432711), Sema3d (#488111), Tcf21 (#508661), Tbx18 (#515221), Scx (#439981), tdTomato (#317041), Rgs5 (#430181), Dpt (#561511), Pecam1 (#316721), Aplnr (#436171), Postn (#418581), Upk3b (#823881), Lhx2 (#485791). The 3-plex negative probe against *dapB* was used as a negative control (#320878). The RNAscope Multiplex Fluorescent Reagent Kit v2 assay was performed according to manufacturer's instructions, except for the target retrieval step which was reduced to 10min and the Protease Plus digestion which was performed at room temperature (RT). The TSA plus fluorophores (PerkinElmer, NEL753001KT, NEL745E001KT) were used at 1:500 final concentration for Fluorescein, 1:1000 for Cy3 and 1:1500 for Cy5.

For immunostaining the cryosections were rehydrated for 15 min in PBS, then permeabilised in PBS + 0.5% Triton X-100 for 15 min at RT, followed by blocking in PBS + 10% goat serum +1% bovine serum albumin (BSA) for 1h at RT and incubation with primary antibody diluted in blocking buffer overnight at 4°C. The sections were rinsed three times, then washed three times in PBS + 0.1% Triton X-100, then the secondary antibodies diluted in blocking buffer were added for 1 hour, RT. The sections were rinsed three times in PBS containing 0.1% Triton X-100, then washed twice for 5 min/each wash, followed by 5 min incubation with DAPI nuclear stain. The slides were mounted in Vectashield (Vector Laboratories, H-1000). Antibodies used: WT1 1:100 dilution (Abcam, ab89901), CD31/Pecam1 1:100 dilution (Abcam, ab119341), SEMA3D 1:50 dilution (Abcam, ab198849), no triton/permeabilization, LYVE1 1:100 dilution (eBiosciences, 50-0443-82), sarcomeric a-actinin 1:100 dilution (Abcam; ab9465), Estrogene receptor alpha (ERa) 1:100 dilution (Abcam, ab16660).

Sections were imaged using a Leica DM6000 fluorescence microscope and Olympus Fluoview-1000 confocal microscope and processed using Fiji.

PrimeFlow RNA Assay & Flow Cytometry. Hearts were enzymatically dissociated using the Neonatal Heart Dissociation Kit (#130-098-373) and the gentleMACS Octo Dissociator with heaters (#130-096-427) from Miltenyi Biotec according to manufacturer's instructions. The dissociated hearts were further processed using the PrimeFlow RNA Assay Kit from ThermoFisher Scientific (#88-18005; (Lai et al., 2018)). Briefly, cells were surface stained with the Zombie Aqua Fixable Viability Kit (Biolegend, #423101) for 0.5hr at RT, 1:1000 dilution, followed by BV421 rat anti-CD31 antibody (Biolegend, #102423) for 0.5hr on ice, 1:50 dilution. Samples were further fixed and permeabilised using reagents provided in the kit. Intracellular staining was performed using BV421 mouse anti-cardiac troponin T (cTNT) antibody (BD Biosciences, #565618; 1:200 dilution), and AlexaFluor 488 anti-Wilms Tumor Protein 1 (WT1) antibody (Abcam, ab202635; 1:100 dilution) for 0.5hr on ice, followed by further fixation and hybridization as specified in the instructions. PrimeFlow catalogue probes were used against Scx (#VB1-3028071-PF), Tbx18 (#VB4-3113124-PF), Tcf21 (#VB6-3197774-PF), Sema3d (#VB1-3044712-PF), Wt1 (#VB4-13886-PF), Npr3 (#VB1-3031000-PF), Rgs5 (#VB4-3226783-PF), Dpt (#VB1-3034260-PF). Probes against dapb (#VF1-11712-PF, # VF6-10407-PF, # VF4-10408-PF) were used as negative controls and probes against Actb (#VB6-12823-PF, # VB1-10350-PF, # VB4-10432-PF) as positive controls.

For analysing the contribution of the epicardial lineage to endothelium, hearts were dissociated and surface stained with viability dye as described above, then pre-treated for 5 min on ice with TruStain FcX (anti-mouse CD16/32) antibody (Biolegend, #101319), 1:50 to block Fcγ receptors. Cells were subsequently stained with BV605 rat anti-CD31 antibody, (Biolegend, #102427) on ice for 0.5hr, 1:50 dilution. Cells were fixed and permeabilised using the PrimeFlow kit reagents, then intracellularly stained with cTNT (as described above). Cells were stored in IC fixation buffer (ThermoFisher, #00-8222-49) until acquisition.

All samples were analysed using BD LSRFORTESSA X-20 cytometer and processed using FlowJo software. Percent positive was calculated using Super-Enhanced Dmax Subtraction algorithm (%SED; FlowJo) when comparing histograms (Supplementary Information Table S1-S3). Geometric mean fluorescence intensity (gMFI) was calculated by subtracting fluorescence minus one (FMO) gMFI from sample gMFI.

Single-cell RNA-sequencing & Analysis. E9.25 single cell microdissected embryo data was downloaded as raw UMI matrix from UCSC Cell Browser (de Soysa et al., 2019). Cells with more than 10% mitochondrial reads were filtered out. E10.5 SMART-Seq2 heart data were downloaded as raw counts from Gene Expression Omnibus (GSE76118) (Li et al., 2016). Cells with more than 2% mitochondrial reads and more than 10000 genes per cell were filtered out. E10.5 single cell heart transcriptome data was downloaded as TPM from Gene Expression Omnibus (GSM3027035) (Dong et al., 2018). The TPM

values were log10 transformed and all cells were kept for downstream analysis. All datasets were analysed using Seurat in R (http://www.R-project.org/ (Hafemeister and Satija, 2019; Stuart et al., 2018). In brief, the significant principal components were used to classify the cells into clusters and the tSNE and UMAP methods were used to visualise the clusters (Becht et al., 2019; Butler et al., 2018; Platzer, 2013).

For the E15.5 single-cell RNA-Sequencing data, the ventricles from 6 embryonic hearts were dissociated (as described above) and BD FACSAria III was used to sort cells positive for tdTomato fluorescence into 96-well plates. The cells were processed according to the Smart-Seq2 protocol (Picelli et al., 2014). Tagmentation and library preparation was done using the Nextera XT DNA Library Prep kit and sequenced using the NextSeq 500/550 High Output Kit v2 (75 cycles) 400 million reads (Illumina, #FC-404-2005) on Illumina NextSeq 500 platform. BCL files from the sequencer were converted to FastQ with bcl2fastq (Illumina, v.2.19.1.403) using default settings. Samples with fewer reads in total than the empty wells were discarded. Reads were trimmed using default settings and "--nextera" for adapter clipping (TrimGalore v.0.4.4). Random samples were checked with FastQC (v.0.11.3) to control for the quality of the sequence. Reads were aligned to the mouse genome (GRCm38/Mm10) with STAR (v.2.3.3a) in gene counting mode with Gencode (v.M16) annotations minus the megatranscript Gm20388 (Dobin et al., 2013). Splice junctions from all samples were combined after the 1st pass alignment. Non-canonical junctions and junctions covered with < 10 reads were removed prior to running the 2nd pass alignment. The samples were further processed with Seurat. Genes with > 200 reads in at least 3 cells were retained for downstream processing. Cells were filtered to retain those with $< 5\%$ mitochondrial genes and at least 500 expressed genes. 276 cells passed this threshold. Reads were normalized to sequencing depth and scaled. Cell cycle scores (G2M and S) was predicted using G2M and S phase genes (Kowalczyk et al., 2015). The difference between the G2M and S phase score was regressed out using the cell cycle regression Seurat vignette. Clustering was performed as described above. The normalised data from Seurat were imported into Monocle2 for analysis of pseudotime (Qiu et al., 2017; Trapnell et al., 2014).

Supplemental References

Becht, E., McInnes, L., Healy, J., Dutertre, C.-A., Kwok, I.W.H., Ng, L.G., Ginhoux, F., and Newell, E.W. (2019). Dimensionality reduction for visualizing single-cell data using UMAP. Nature Biotechnology *37*, 38-44.

Butler, A., Hoffman, P., Smibert, P., Papalexi, E., and Satija, R. (2018). Integrating single-cell transcriptomic data across different conditions, technologies, and species. Nat Biotechnol *36*, 411-420.

de Soysa, T.Y., Ranade, S.S., Okawa, S., Ravichandran, S., Huang, Y., Salunga, H.T., Schricker, A., del Sol, A., Gifford, C.A., and Srivastava, D. (2019). Single-cell analysis of cardiogenesis reveals basis for organ-level developmental defects. Nature *572*, 120-124.

Dobin, A., Davis, C.A., Schlesinger, F., Drenkow, J., Zaleski, C., Jha, S., Batut, P., Chaisson, M., and Gingeras, T.R. (2013). STAR: ultrafast universal RNA-seq aligner. Bioinformatics (Oxford, England) *29*, 15-21.

Dong, J., Hu, Y., Fan, X., Wu, X., Mao, Y., Hu, B., Guo, H., Wen, L., and Tang, F. (2018). Single-cell RNA-seq analysis unveils a prevalent epithelial/mesenchymal hybrid state during mouse organogenesis. Genome Biol *19*, 31.

Hafemeister, C., and Satija, R. (2019). Normalization and variance stabilization of single-cell RNA-seq data using regularized negative binomial regression. Genome Biology *20*, 296.

Kowalczyk, M.S., Tirosh, I., Heckl, D., Rao, T.N., Dixit, A., Haas, B.J., Schneider, R.K., Wagers, A.J., Ebert, B.L., and Regev, A. (2015). Single-cell RNA-seq reveals changes in cell cycle and differentiation programs upon aging of hematopoietic stem cells. Genome research *25*, 1860-1872.

Lai, C., Stepniak, D., Sias, L., and Funatake, C. (2018). A sensitive flow cytometric method for multi-parametric analysis of microRNA, messenger RNA and protein in single cells. Methods *134-135*, 136-148.

Li, G., Xu, A., Sim, S., Priest, J.R., Tian, X., Khan, T., Quertermous, T., Zhou, B., Tsao, P.S., Quake, S.R., *et al.* (2016). Transcriptomic Profiling Maps Anatomically Patterned Subpopulations among Single Embryonic Cardiac Cells. Developmental cell *39*, 491-507.

Madisen, L., Zwingman, T.A., Sunkin, S.M., Oh, S.W., Zariwala, H.A., Gu, H., Ng, L.L., Palmiter, R.D., Hawrylycz, M.J., Jones, A.R., *et al.* (2010). A robust and high-throughput Cre reporting and characterization system for the whole mouse brain. Nature neuroscience *13*, 133-140.

Picelli, S., Faridani, O.R., Björklund, Å.K., Winberg, G., Sagasser, S., and Sandberg, R. (2014). Full-length RNA-seq from single cells using Smart-seq2. Nature Protocols *9*, 171.

Platzer, A. (2013). Visualization of SNPs with t-SNE. PloS one *8*, e56883-e56883.

Qiu, X., Mao, Q., Tang, Y., Wang, L., Chawla, R., Pliner, H.A., and Trapnell, C. (2017). Reversed graph embedding resolves complex single-cell trajectories. Nat Methods *14*, 979-982.

Stuart, T., Butler, A., Hoffman, P., Hafemeister, C., Papalexi, E., Mauck, W.M., Stoeckius, M., Smibert, P., and Satija, R. (2018). Comprehensive integration of single cell data. bioRxiv, 460147.

Trapnell, C., Cacchiarelli, D., Grimsby, J., Pokharel, P., Li, S., Morse, M., Lennon, N.J., Livak, K.J., Mikkelsen, T.S., and Rinn, J.L. (2014). The dynamics and regulators of cell fate decisions are revealed by pseudotemporal ordering of single cells. Nat Biotechnol *32*, 381-386.

Wang, F., Flanagan, J., Su, N., Wang, L.C., Bui, S., Nielson, A., Wu, X., Vo, H.T., Ma, X.J., and Luo, Y. (2012). RNAscope: a novel in situ RNA analysis platform for formalin-fixed, paraffin-embedded tissues. J Mol Diagn *14*, 22-29.

Zhou, B., Ma, Q., Rajagopal, S., Wu, S.M., Domian, I., Rivera-Feliciano, J., Jiang, D., von Gise, A., Ikeda, S., Chien, K.R., *et al.* (2008). Epicardial progenitors contribute to the cardiomyocyte lineage in the developing heart. Nature *454*, 109-113.
